# Supplementary material for: The immunoglobulin G antibody response to malaria merozoite antigens in asymptomatic children co-infected with malaria and intestinal parasites
Source: PLoS One. 2020 Nov 10;15(11):e0242012. doi: 10.1371/journal.pone.0242012 (PMC7654760; doi:10.1371/journal.pone.0242012)
Supplement: S1 Table — (DOCX) [file pone.0242012.s001.docx]

| S1 Table: Influence of co-infections on anemia and eosinophilia | | | | | |
| --- | --- | --- | --- | --- | --- |
|  | MAL-IP- | MAL-IP+ | MAL+IP- | MAL+IP+ |  |
| number | 69 | 7 | 190 | 54 |  |
| Mean age +/- SD (years) | 6.4 ± 3.8 | 8.6 ±2.7 | 7.9 ± 3.6 | 9.3 ± 2.8 | 0.0001* |
| Percentage with anaemia  (number anemia/total) | 30.4%  (21/69) | 28.6  (2/7) | 46.3  (88/190) | 35.2  (19/54) | 0.0588** |
| Percentage with eosinophilia | 5.8%  (4/69) | 57.1  (4/7) | 13.7  (26/190) | 24.1  (13/54) | 0.0004*** |
| *Age: ANOVA comparison of the 4 groups (p=0.0001). Pairwise comparisons: MAL-,IP- vs MAL+,IP- (p=0.0041); MAL-IP- vs MAL+,IP+ (p <0.0001); MAL-,PI+ vs MAL+,IP+ (p = 0.5359).  **Anaemia: among the 4 groups (Chi Square p = 0.0588); Pairwise comparisons using Fisher’s Exact Test: MAL-,IP-, vs MAL+,IP- p=0.0234; MAL-,IP- vs MAL-,IP+ p>0.999; MAL+,IP- vs MAL+,IP+ p=0.1368; MAL-,IP- vs MAL+,IP+ (p = 0.6984)  ***Eosinophilia: Among the 4 groups, (Chi Square p=0.0004). Pairwise comparisons: MAL-,IP- vs MAL-,IP+ (Fisher p =0.0017); MAL-,IP+ vs MAL+,IP+ (Fisher p = 0.0870); MAL+,IP- vs MAL+,IP+ (Fisher p =0.0904) | | | | | |

Summary:

Age: A significant difference was found in pairwise comparisons between each of the age groups, except between MAL- and MAL+ who were IP+. Overall, children with malaria were younger and those with IP were older.

Anaemia: A significant difference was found only between MAL-,IP- and MAL+,IP- (p = 0.024); One might have expected a difference in prevalence of anaemia between MAL-,IP- and MAL+,IP+ (p=0.6987), but a difference was not found. Reduced anaemia in coinfected children MAL+,IP+ compared to MAL+ alone, may be due to children with IP+ being older, who may have acquired adequate immunity to partially control their parasitemia, resulting in lower prevalence of anaemia.

Eosinophilia: A significant difference was found between MAL-,IP- and MAL-,IP+ (p=0.0017), with near borderline significant between MAL+ children with single infections and co-infections (p=0.0904).
