# Supplementary material for: The immunoglobulin G antibody response to malaria merozoite antigens in asymptomatic children co-infected with malaria and intestinal parasites
Source: PLoS One. 2020 Nov 10;15(11):e0242012. doi: 10.1371/journal.pone.0242012 (PMC7654760; doi:10.1371/journal.pone.0242012)
Supplement: Attachment — Submitted filename: PONE-D-20-12369_R1_reviewer2.pdf [file pone.0242012.s005.pdf]

# PLOS ONE

## Full-title: The immunoglobulin G antibody response to malaria merozoite antigens in asymptomatic children co-infected with malaria and intestinal parasites

--Manuscript Draft--

| | |
|---|---|
| **Manuscript Number:** | PONE-D-20-12369R1 |
| **Article Type:** | Research Article |
| **Full Title:** | Full-title: The immunoglobulin G antibody response to malaria merozoite antigens in asymptomatic children co-infected with malaria and intestinal parasites |
| **Short Title:** | Malaria antibodies in children with intestinal parasites |
| **Corresponding Author:** | crespo'o mbe-cho ndiabamoh<br>Biotechnology Center Nkolbisson Cameroon<br>Yaounde, CAMEROON |
| **Keywords:** | malaria, intestinal-parasites, antibody, Giardia lamblia, Entamoeba histolytica |
| **Abstract:** | BackgroundCo-infection with malaria and intestinal parasites is common in children in Africa and may affect their immune response to a malaria parasite infection. Prior studies suggest that co-infections may lead to increased susceptibility to malaria infection and disease severity; however, other studies have shown the reverse. Knowledge on how co-morbidities specifically affect the immune response to malaria antigens is limited. Therefore, this study sought to determine the prevalence of co-infection of malaria and intestinal parasites and its association with antibody levels to malaria merozoite antigens.Methods A cross sectional study was carried out in two villages with high transmission of malaria in Cameroon (Ngali II and Mfou). Children aged 1-15 years were enrolled after obtaining parental consent. A malaria rapid diagnostic test was used on site. Four (4) ml of peripheral blood was collected from each participant to determine Plasmodium falciparum infections by microscopy, haemoglobin levels and serology. Fresh stool samples were collected and examined by wet mount, Kato-Katz method and modified Ritchie concentration techniques. A Multiplex Analyte Platform assay was used to measure antibody levels. ResultsA total of 320 children were enrolled. The prevalence of malaria by blood smear was 76.3% (244/230 and prevalence of malaria and intestinal parasites was 16.9% (54/320). Malaria prevalence was highest in young children; whereas, intestinal parasites (IP+) were not present until after 3 years of age. All children positive for malaria had antibodies to MSP142, MSP2, MSP3 and EBA175. No difference in antibody levels in children with malaria-co infections compared to malaria alone were found, except for antibody levels to EBA-175 were higher in children co-infected with intestinal protozoa (p = 0.018), especially those with Entamoeba histolytica infections (p=0.0026).Conclusion Antibody levels to EBA175 were significantly higher in children co-infected with malaria and E. histolytica compared to children infected with malaria alone. It is important to further investigate why and how the presence of these protozoans can modulate the immune response to malaria antigens. |
| **Order of Authors:** | Crespo'o Mbe-cho Ndiabamoh |
| | Gabriel L. Ekali |
| | Yukie M. Lloyd |
| | Livo Esemu |
| | Wilfred Mbacham |
| | Jude D. Bigoga |
| | Diane W. Taylor |
| | Rose G.F. Leke |
| **Response to Reviewers:** | Reviewer #1: Dr. Mbe-cho and colleagues sought to determine the prevalence of co-infection of malaria and intestinal parasites and its association with antibody levels to malaria merozoite antigens. The authors report that there was no difference in antibody prevalence or levels in malaria-infected and co-infected children, except antibody |

levels to EBA-175 were significantly higher in children co-infected with malaria and E. histolytica. Overall, the study is well-designed but these results do not significantly alter or impact our understanding of the association of malaria and helminths on antibody to malaria merozoite antigens.

1. The limitation of the study is that the parasite testing in children was not followed by sensitive diagnostic techniques like PCR, and light infections may have been missed which may have resulted in misclassification of the groups. Light infections may boost the antibody responses while children remain asymptomatic.
Reply: We understand the concern. When the study was conducted (2017) in the rural villages, the prevalence of slide-positive malaria was 75.6%. In a prior study conducted in the village (Leke et al 2010), an equivalent prevalence was found of P. falciparum (50-85%) in children aged 5-15 years over a 5-year period. The estimated entomological inoculation rate (EIR) was 0.7 infectious bites/person/ nightly thought out the year (~257 IB/P/Y). Based on the more recent malaria prevalence, it appears that the current EIR is similar. Thus, children were most likely being bitten approximately every-other night by an infectious mosquito, since bednets were not routinely used. With this high level of transmission, most of the slide-negative children would be PCR-positive for malaria, i.e., have enough immunity to reduce malaria to submicroscopic levels. Unfortunately, in very high transmission areas like the one reported herein, everyone will have some circulating P. falciparum parasites. So, classifying subjects as slide-positive vs slide-negative may not reflect presence/absence of parasites, but provide information on the immune status of the person. In revising the MS, information from the study by Leke et al. was included as well as a discussion of submicroscopic infections in the revised Discussion.

2. In this study, only 3.4% children were infected with helminths alone to get any meaningful data for antibody response to malaria in this group.
Reply: We agree, the sample size of children with helminth infections is too small to provide meaningful information. Accordingly, Ab levels in children with helminth infections were not analyzed. To explain the low prevalence of helminths, information on the Ministry of Heatlth's policy for biannual treatment of children for worms was provided.

3. Very few children are positive for E. histolytica.
Reply: True, the prevalence of Entamoeba in our study was only 5.9%, which is lower than that reported in studies in these areas of ~23% (T. E. Kwenti et al., 2016). In our study, the prevalence was lower, probably due to rigorous mass drug administration (MDA) programs implemented by the Ministry of Health and other regular or seasonal health campaigns.

4. The data on the children's anthropomorphic measurements are not mentioned. Thus, there is not much point describing how they were collected.
Reply: This section was removed from the Methods section.

5. There is no data on hookworm infection in the results.
Reply: The prevalence of hookworm infections was considered in this study during stool exams and, surprisingly, we did not find hookworms in the samples collected, most likely due to regular deworming and improved hygiene in the area. No invasive methods were used for diagnosis of adult worms. From a paper published by E. Kwenti et al. (2016) the prevalence of hookworm was 7% in south west region Cameroon.

6. The number of eggs per gram of stool were estimated for the parasites listed. Did the authors look at the responses in children with high or low intensity of the parasites?
Reply: In this study, after obtaining the prevalence of parasites and comparing with antibody response, no significant difference was observed between the malaria antibodies levels and parasites eggs counts.

7. Table 2 is not necessary, it can be written as text.
Reply: Thanks for the comment, but we think Table 2 summarizes the data more clearly and allows readers to easily compare results from different groups than presenting them in the text. Table 2 has been revised.

8. Page 21, reference # 54, year of publication is missing.

| | |
|---|---|
| | Reply: Year of publication has been included.<br><br>Please check spelling and typographical errors scattered through the manuscript (page and lines are given from word document):<br>1. Page 2, line 3, change led to lead in the sentence.<br>Reply: The word “led” has been changed to “lead”.<br><br>2. Page 2, line 14, correct the spelling of Rietchi concentration method<br>Reply: Spelling has been corrected to “Ritchie”<br><br>3. Page 6, line 21: The bracket has to be closed here: (AB Leo Diagnostics, Helsingborg, Sweden.<br>Reply: The bracket has been closed.<br><br>4. Page 7, line 17 and 18: Correct 50ul to 50µl<br>Reply: The change has been made.<br><br>5. Page 9 and 10: In the text, the p value for anemia (MAL+,IP-) is p=0.034; p value for the same in Table 1 is p=0.032; it needs to be corrected.<br>Reply: P value has been corrected to P=0.032 (correct value) in the text.<br><br>6. Page 10: In Table 1, % sign is missing in column 5 for children with Hb.<br>Reply: The % symbol has been included in table 1, column 5.<br><br>7. Page 10, line 3: In the sentence, change major to majority.<br>Reply: The word “major” has been changed to “majority”.<br><br>8. Page 14, line 27: In the sentence, MSL- should be MAL-<br>Reply: In Line 27 of page 14, MSL- has been changed to MAL-<br><br>9. Page 17, line 15: change beats to beads<br>Reply: The spelling of beads has been corrected.<br><br>10. Re-write the following sentences, they are not very clear:<br><br>Page 4, line 8:<br>However, with most children getting infected with several episodes of infections in a short period, this renders them more prone to having clinical symptoms since the immune systems doesn’t fully recover.<br>Reply: The sentence has been deleted because the information is not directly relevant to the study.<br><br>Page 4, line 20:<br>Concomitant infections in humans have suggested that Ascaris lumbricoides infection may protect against cerebral malaria (11,12), while other studies, children infected by S. mansoni were more susceptible to P. falciparum infection and develop acute malaria episodes.<br>Reply: The sentence has been revised to read: “Studies on concomitant infections in humans suggest that A. lumbricoides infection may protect against cerebral malaria (11,12), while other studies suggest that children infected by S. mansoni may be more susceptible to P. falciparum infections and develop acute malaria episodes (13,14).”<br><br>Page 15, line 3:<br>In essence, the immune response in individuals who are repeatedly infection would be similar to that produce during chronic infections.<br>Reply: To clarify the statement, the text has been revised to read: “Because of high transmission, the children are becoming infected almost daily and are either in the process of eliminating the new infection or reducing it to a submicroscopic level. Because of constant re-exposure, the resulting immune response will be similar to that produced by a chronic infection.<br><br><br><br>Reviewer #2: The answer to the questions is divided into Major comments, Minor |

Powered by Editorial Manager® and ProduXion Manager® from Aries Systems Corporation

comments. Additionally, I wrote minor observations that, I hope, will help this manuscript to improve readability and consistency.

1. Is the manuscript technically sound, and do the data support the conclusions?
2. Has the statistical analysis been performed appropriately and rigorously?
3. Have the authors made all data underlying the findings in their manuscript fully available?
4. Is the manuscript presented in an intelligible fashion and written in standard English?

Major comments:

• Given that there were no differences in the IgG response between age groups, it would be interesting to join these data, evaluate all the coinfected individuals, and then split the data into Giardia, E. hystolitica.
Reply: We are confused by this comment, because Fig 1 shows an increase in both Ab prevalence (Fig. 1A) and Ab levels (Fig 1 B-E) with age in Ab-positive children (Kruskal-Wallis test p values were p<0.001 MSP2 and p=0.05-0.086 (borderline) for the other antigens).

We believe combining all MAL+,IP+ children into single a group is unwise, since they were infected with a conglomerate of intestinal helminths, cestodes and protozoa (see Table 2). Combining children with such heterogenous infections is unlikely to provide meaningful information.

• I strongly suggest dividing the age of individuals in 0-5, 5-10, 10-15 years-old to partially solve the "N" problem of the groups.
Reply: Thanks for the comment. Initially, children were groups into 5-year categories as suggested by the Reviewer, i.e., 0-5, 5-10, 10-15 years old. However, when the data set showed that children aged 1 to 2 did not have intestinal parasites, the results were grouped into 2-year intervals, that allowed us to more closely define the increase in Ab prevalence (Fig. 1A) and Ab levels (Fig 1 -B,C,D,E) with age. The purpose of Fig 1 was to determine if age was a variable that needed to be taken into consideration during data analysis.

• Because of the absence of molecular Diagnosis and considering that the authors mention the possibility of oh having low parasitemia infections in the MAL- group. It is important to include MAL- individuals in Figure 1.
Reply: We are sorry if we didn't make the point clear. ALL children who were Ab-positive are included in Fig 1, including those who are MAL+ and MAL-. Because malaria transmission is high in the area, all children in the study had been exposed to P. falciparum and many of the MAL- children were Ab-positive.

• It is necessary to compare parasite data with similar regions in Cameroon. Please compare and cite:
• (Malaria and Helminth Co-Infection in Children Living in a Malaria Endemic Setting of Mount Cameroon and Predictors of Anemia from Theresa K Nkuo-Akenji et al. 2006)
• Malaria, Helminths, Coinfection and Anaemia in a Cohort of Children From Mutengene, South Western Cameroon from Clarisse Njua-Yafi et al. 2016.
Reply: We thank the Reviewer for pointing out the omission of key references. Information from these studies have been included in the revised Discussion. The text now reads, "…..to those found in other highly [malaria] endemic regions of the country (32), and the prevalence of co-infections was 19.1%, which is similar to the prevalence of co-infections of 18 – 27% reported in other regions of Cameroon (9,44). The references have been added to the reference section.

• Do the authors have information about malaria and intestinal parasites last treatments? On page 17, it was commented that Albendazole treatment was frequent in these children. Deworming information will help the readers to understand why the prevalence of intestinal parasites was low compared with other studies in Cameroon. Additionally, reinforce in the discussion section that collecting/reporting that information is valuable for coinfection studies.
Reply: In response to the Reviewer's suggestion, the following information has been added to the Methods section. "Currently, mass drug administration with albendazole is being performed twice a year by the Ministry of Health, that is usually conduced in

Powered by Editorial Manager® and ProduXion Manager® from Aries Systems Corporation

schools and symptomatic cases are sent to the local clinic or hospital for follow up treatment."

• (Figure 1 B, C, D, E) use the same scale limits for all plots. This is also useful to understand differences in levels of antigenicity between proteins.
Reply: We understand the comment, but we do not wish to change the Y-axis on Fig 1, since it is risky to make a direct comparison of Ab levels between antigens in serological assays. A number of variables, including parasite strain, the system to produce recombinant proteins, protein purity, the amount of antigen used, number of exposed epitopes, dilution of plasma, etc., influence the overall results. Even when Luminex beads are covalently-coupled with saturating amounts of antigen, it is questionable if direct comparison of MFI can be made between antigens. Although our assays have been optimized and equivalence amounts of antigen used during bead-coupling, comparisons among the antigens may not provide accurate information about immunogenicity. In Figs1 B, C, D, E, the Y-Axis was selected to show the best distribution of the MFI results.

• (table 3) How could the authors explain increased eosinophilia with low levels of helminth infection? This mainly applies to the age group > 9 years-old.
Reply: After age 2, children start becoming infected with helminths, resulting in an increase in eosinophil counts. During the biannual drug treatment campaign, helminthic infections are eliminated, but eosinophilia persists for a period of time. With increasing age, more children in the area become i) infected and ii) re-infected, resulting in an increase in prevalence of eosinophilia.

• (Page 17) The authors argue, "First, children living in moist or wet environments where mosquitoes breed and E. histolytica are more abundant would have a high risk of acquiring both infections, that would result in frequent boosting of the Ab response." This explanation for intestinal parasite influence on antibody production alteration is not viable since Giardia's frequency is higher than E. histolytica in the studied population.
Reply: The sentence has been deleted from the Discussion.

• (Page 17) The affirmation "Secondly, since malaria and E. histolytica are both amoebae, they might share common antigens, for example, EBA-175 could share homology with an E. histolytica antigen." is false. Plasmodium falciparum is not an is a protozoan. This group belongs to Apicomplexa organisms. For that reason, the hypothesis about correlating Plasmodium and E. histolytic is wrong.
Sorry, "amoebae" was a typo. Both Plasmodium falciparum and E. histolytica are protozoans. The Discussion has been revised to read "parasitic protozoa."

• How different are the two Villages Ngali II and Mfou in the central region of Cameroon? Does it exist a difference in humidity and soil moist, once the authors claimed that this variable could explain differences of Entamoeba histolytica?
Reply: The two villages are very similar with no major differences in humidity or soil moisture. The estimated annual average rainfall measures 1600 mm3 with an annual average temperature of 23°C for Ngali II and for Mfou. According to the National Meteorology agency, the average humidity for the center regions is 83%. Ngali and Mfou are both in the center region of Cameroon about 60km apart. Note: as mentioned above, the words "humidity and soil moisture" have been deleted from the MS.

Minor comments:
• What criteria were used to divide the population into seven groups according to age?
Reply: The fact that Intestinal parasite (IP) infections was only observed in children >2 years, helped guide separation of the children into seven groups.

• Please specify how anthropometric parameters were used in the study, once they were described but not used in the study. If this information was not used, please remove these sentences.
Reply: The sentence has been removed.

• Has the studied region presence of Schistosoma haematobium? If the authors have register if this parasite in the area, Did they examined urine samples to discard infections with this parasite?
Reply: Detection of S. haematobium was not included in the study design because of

Powered by Editorial Manager® and ProduXion Manager® from Aries Systems Corporation

low prevalence in the study area. A study conducted in this area (and other regions of Cameroon) by Louis-Albert Tchuem Tchuenté et al., (2012) reported a prevalence of S. haematobium of only 1.72%. Since a large sample size would be required to assess the impact of this pathogen on the Ab response to malaria, S. haematobium was not included in the study.

• Were the individuals asymptomatic to intestinal parasites infection too? No diarrhea, abdominal pain, etc.? Please clarify.
Reply: Yes. To make the point clear, the Methods section has been revised and states that all children with clinical cases of malaria or intestinal parasites were not included in the study and referred to the local clinic/hospital by the attending physician for treatment. Thank you for the comment.

• (Page 6) It was mentioned that Plasmodium parasitemia was quantified. Did the authors observe any correlation between the Plasmodium parasite burden and the levels of IgG responses to the antigens?
Reply: As expected, there was no correlation between parasitemia and malaria antibody levels.

• (End of Page 7) Please specify: If the cut-off is MFI+3*SD, how the standard deviation was calculated if the negative controls were pooled? Was this experiment repeated or used replicates? Traditionally, the negative controls are tested simultaneously in different wells of the plate, and the cut-off is calculated from those values.
Reply: Pooled negative control plasma sample were run in triplicates on the same plates as the test samples in all experiments, as well as the positive controls. The cut-off was obtained by calculating MFI+3 SD of the triplicates on all plates in the experiment.

• Did the authors analyze the effect of helminth parasite burden (number of eggs/gram of stool) in those individuals with helminths? This valuable information was commented on but never included in the analysis. If not used,e I do not see the necessity of describing in the methods section
Reply: The information has been deleted from the Methods section.

• For data analysis:
• Before using ANOVA, did the authors checked for the normality of the variables? If yes, please specify, if not, calculate the normality of the variables and the other ANOVA assumptions.
Reply: Yes, ANOVA was used to compare difference in age across the 4 groups (Table 2). However, comparisons of Ab MFI, which are not normally distributed, with age (Fig. 1) were performed using the Kruskal-Wallis test. The Methods section (Data analysis) has been revised. Information in Fig. 1 legend was correct.

• If the authors have not-normal variables, they should use the Kruskal-Wallis non-parametric, and Dunn posthoc tests to verify differences between groups.
Reply: Sorry for the mistake in the Methods section. The Kruskal-Wallis nonparametric test was performed in Fig 1 and 2. A posthoc test was not performed, as the goal was not to determine when peak Ab levels were obtained, but to determine if age had an influence on Ab levels. Since age was a variable, data for all age groups could not be combined, but rather age was taken into consideration during data analysis.

• Please check frequencies described in table 1 (MAL+IP- 58.8%) vs. the values reported in the second line page 9. (59.4%).
Reply: 59.4% is the correct value. The text has been revised.

• Sum of 58.8%+16.9% = 75.7% not 75.6%.
Reply: Thank you for catching the error. The values in Table 1 and text have been revised and are now consistent.

• In table 1, please add a column with P-values to facilitate the interpretation of the differences between groups. Please report statistics of multiple comparisons between groups too.
Reply: The comparisons requested by the reviewer were originally provided in the

Powered by Editorial Manager® and ProduXion Manager® from Aries Systems Corporation

| | |
|---|---|
| | Table legend. To comply with the request, the p values have been moved to a column labeled "p values" and the method of analysis was retained in the Table legend.<br><br>• What is the potential hypothesis to explain the increased values of parasitemia in the coinfected group?<br>Reply: There is no significant difference in parasitemia between the two groups (p=0.1599). In fact, the higher parasitemia was found in young children who were intestinal parasite-negative (probably because very young children were in this group).<br><br>• Please comment in the text the presence of multi-parasitism in the studied individuals.<br>Reply: We thank the reviewer for the comment. The following sentence has been added to the Results section. "Interestingly, all of the children had single parasite infections, and polyparasitism was not found."<br><br>• (Page 11 table 3). Please include values of anemia and eosinophilia in individuals coinfected. In the current configuration is constructed is hard to determine the coinfection impact in anemia and eosinophilia values.<br>Reply: Table 3 was designed to evaluate the influence of age on malaria, IP, anemia and eosinophilia. The number of co-infections are too small to be divided by age. In an attempt to address the Reviewer's comment, a separate Table was designed that compares the influence of no infections, malaria-positive only, and co-infections on percent with anemia and eosinophilia. The Table will be up-loaded as supplemental Table 1. It essentially showed that same results as expected, anemia was associated with malaria and eosinophils were associated with co-infections.<br><br>• (Page 11). In the sentence, "Thus, as children living in these villages increased with age, they developed partial immunity to malaria and anemia declined; whereas, the prevalence of IP and eosinophilia increased." In this sentence, it is necessary to specify that "protection" is protection against malaria symptoms. The table clearly shows that the frequency of malaria does not decrease with age, only the anemia.<br>Reply: The sentence has been revised to read: "Thus, as children living in these villages increased with age, they began developing partial immunity to malaria symptoms and anemia declined; whereas, the prevalence of IP and eosinophilia increased.<br><br>• Please plot Age vs. Antibody levels for each protein to verify the correlation for each protein studied.<br>Reply: The figure on the right confirms that Ab levels increase with age. The figure shows a linear regression analysis of Ab levels for MSP1, MSP2, MSP3 and EBA-175 using data from all 320 children, and includes the equation for the regression line, the R2 value (all positive), and p value (all significant). Thus, the figure confirms that Ab levels increase with age. We do NOT wish to include this figure in the MS since it is essentially identical to the one shown in Fig 1 B, C, D and E. In fact, we feel that the information in Fig 1B-E is easier for the reader to understand.<br>Note: If the figure is not shown, it is provided in a separate document.<br><br>• As an exploratory analysis, I suggest joining all data and make a boxplot comparing MFI between MAl-PI-, MAL-PI+, MAL+PI-, and MAL+PI+. Mainly for MSP1, MPS2, and MSP3 group age 3-10 and 11-15 to check.<br>Reply: We thank the Reviewer Thanks for the suggestion concerning exploratory analysis. A comparison of Ab levels in two of the above groups (MAL-,IP-, and MAL+,IP-) is shown in Fig 2. Unfortunately, the number of children in the MAL-,PI+ group is too small to provide valuable information. As stated above, children in the MAL-,PI+ group (n=54) are infected with a variety of intestinal helminths, cestodes and protozoa (see Table 2). With such a diverse range of pathogens, plotting the data as a boxplot will not provide useful information. In Fig. 2, the distribution of Ab levels in children co-infected with malaria and single intestinal pathogens is provided. We feel this approach is more informative than "dumping all pathogens together."<br><br>• The sentence "E. histolytica is a gut amoeba that causes both intestinal and extraintestinal infections such as amebic colitis (dysentery) and liver or brain abscess. The protozoa cause a marked down-regulation of macrophage functions rendering the cells incapable of antigen presentation and unresponsive to cytokine stimulation (57)" |

does not explain the increase of antibody production in E. histolytica infected group. Why could a diminishing antigen presentation generate higher levels of anti-Plasmodium antigens?
Reply: Very true! Not sure why that statement wasn't caught. The Discussion has been changed significantly. It now reads, "The decrease in macrophage function does not explain the increase in Ab to EBA-175. One possible explanation is that since malaria and E. histolytica…"

Other observations/questions:
• In the title, add "IgG" to Antibody response. Reply: IgG has been added to title (although not all of the co-authors agree this is necessary).

• Check all scientific names of parasite species for correct formatting in italics. (Example Entamoeba histolytica in the Results section in the abstract)
Reply: The scientific name has been checked and are now in italics.

• Please, mention in the background the region where the study was performed.
Reply: This information was included in the background section of the Abstract. It is also included in the Materials section.

• It is necessary to describe and discuss the role of MSP1, MPS2, MSP3, and EBA-175 as markers in serological studies.
Reply: This information has been added to the Discussion.

• Considering that coinfection prevalence is relatively low, I consider that it is important to discriminate with colors or point shapes the individuals MAL-IP-, MAL+IP-, MAL-IP+, MAL+IP+ in Figure 1 B-C-D-E
Reply: We thank the Reviewer for the suggestion. However, information in Fig 1B-E is designed to address the question, are Ab prevalence and levels influence by age? Whereas, Fig 2 provides comparisons between individuals infected with malaria alone or co-infected with specific intestinal parasites. Thus, colored dots or symbols are not needed in Fig 1 (and could be confusing to the reader).

• In page 6 subtitle "Laboratory detection, quantification and speciation of malaria parasites.", I will not use speciation here. I suggest "Diagnosis and quantification of Plasmodium sp. parasites.
Reply: The header has been changed to read: "Laboratory detection of malaria parasites."

• (Page 14-15) What type of parasite is "Amoeba"? What is the difference between "Amoeba" and E. histolytica? Traditionally, E. histolytica is considered an amoeba too.
Reply: The figure has been revised to read Intestinal Protozoa. Thanks for pointing out the mis-classification.

• In table 1, to facilitate reading, please remove symbols % and /ul located in cells with data and add to the columns describing the variables.
Reply: The symbols in the data cells have been removed.

• For consistency, unify parasitemia vs. parasitemia, anemia vs. anemia in the text and plots.
Reply: The British spelling of parasitaemia, anaemia, and haemoglobin have been used through out the MS.

• (Page 10) change "The major of helminth parasites" to "The most frequent helminth species detected."
Reply: The change was made as suggested.

• (Table 2) Check all the total numbers for the "Total IP+" column. For example, for protozoans, the sum is 29+19+4 = 48, and it was reported 47
Reply: This has been verified and corrected to 48 in Table 2

• (Page 13) In plot titles Change Ab (Antibody) to IgG
Reply: We thank the Reviewer for the comment, but decide not to make the change. Our rationale is that by definition, IgG is a class of immunoglobulin found in the blood;

Powered by Editorial Manager® and ProduXion Manager® from Aries Systems Corporation

| | |
|---|---|
| | whereas, Ab are plasma proteins that bind specifically with an antigen. What was measured was IgG Ab. Since the serological assay measured IgG Ab that were recorded as MFI (median fluorescence intensity), we think the labels on the Y-Axis (Ab levels -MFI) reflect what was done. The Methods section makes it clear that the Ab were of the IgG class. [Note: Serum IgG levels (which implies mg/ml) were not measured.]<br><br>• (Figure 1E) Add, Change from EBA to EBA-175.<br>Reply: Change has been made.<br><br>• Please verify all references formatting (For example, reference 42 is all in capital letters)<br>Reply: References have been edited as requested by the reviewer.<br><br>Review #3: Comments were in the attachment.<br>Reply: In revising the MS, all requested changes were made and additional information provided in the text, including information on the BLAST search. The only request we would not fully address is the prevalence of bednet use in the villages. The only information available is that very few children use bednets. Since the slide-positivity rate of 75.6% for P. falciparum, it is unlikely the bednets are having a major influence on the current study. The following information has been added to the MS in the Results section. "To determine if higher Ab levels in children co-infected with P. falciparum and E. histolytica might be due to cross-reactive epitopes, a BLAST search for sequence homology between EBA-175 and E. histolytica proteins was made. No similarities were found using Metablast, and only one hit was found using discontinuous metablast which had a span of only 38 nucleotides (~12 amino acids). Thus, there does not appear to be shared epitopes between these two pathogens that would explain the increase in Ab to EBA-175 in children with co-infections."<br><br>Figure for Reviewer #2 confirming an increase in antibody levels with age. |
| **Additional Information:** | |
| **Question** | **Response** |
| **Financial Disclosure**<br><br>Enter a financial disclosure statement that describes the sources of funding for the work included in this submission. Review the submission guidelines for detailed requirements. View published research articles from *PLOS ONE* for specific examples.<br><br>This statement is required for submission and **will appear in the published article** if the submission is accepted. Please make sure it is accurate. | The author(s) received no specific funding for this work. Funding used in for this research was mentors (Prof Leke Rose) and a Gift of the magnetic beats from Dr Anna Babakhanyan. No other specific funding were received. |

*Powered by Editorial Manager® and ProduXion Manager® from Aries Systems Corporation*

| | |
|---|---|
| **Unfunded studies**<br>Enter: *The author(s) received no specific funding for this work.*<br><br>**Funded studies**<br>Enter a statement with the following details:<br>• Initials of the authors who received each award<br>• Grant numbers awarded to each author<br>• The full name of each funder<br>• URL of each funder website<br>• Did the sponsors or funders play any role in the study design, data collection and analysis, decision to publish, or preparation of the manuscript?<br>• **NO** - Include this sentence at the end of your statement: *The funders had no role in study design, data collection and analysis, decision to publish, or preparation of the manuscript.*<br>• **YES** - Specify the role(s) played.<br><br>* typeset | |
| **Competing Interests**<br><br>Use the instructions below to enter a competing interest statement for this submission. On behalf of all authors, disclose any competing interests that could be perceived to bias this work—acknowledging all financial support and any other relevant financial or non-financial competing interests.<br><br>This statement **will appear in the published article** if the submission is accepted. Please make sure it is accurate. View published research articles from *PLOS ONE* for specific examples. | The authors have declared that no competing interests exist. |

*Powered by Editorial Manager® and ProduXion Manager® from Aries Systems Corporation*

| | |
|---|---|
| **NO authors have competing interests**<br><br>Enter: *The authors have declared that no competing interests exist.*<br><br>**Authors with competing interests**<br><br>Enter competing interest details beginning with this statement:<br><br>*I have read the journal's policy and the authors of this manuscript have the following competing interests: [insert competing interests here]*<br><br>* typeset | |
| **Ethics Statement**<br><br>Enter an ethics statement for this submission. This statement is required if the study involved:<br><br>• Human participants<br>• Human specimens or tissue<br>• Vertebrate animals or cephalopods<br>• Vertebrate embryos or tissues<br>• Field research<br><br>Write "N/A" if the submission does not require an ethics statement.<br><br>General guidance is provided below. Consult the submission guidelines for detailed instructions. **Make sure that all information entered here is included in the Methods section of the manuscript.** | Ethical clearance used for the study was obtained from the Cameroon National Ethics Committee (IRB approval: No2016/12/845/CE/CNERSH/SP). Administrative authorizations were obtained from authorities of the Ngali II and Mfou health districts. Written Informed consents were obtained from parents of all participants. |

| | |
|---|---|
| **Format for specific study types**<br><br>**Human Subject Research (involving human participants and/or tissue)**<br>• Give the name of the institutional review board or ethics committee that approved the study<br>• Include the approval number and/or a statement indicating approval of this research<br>• Indicate the form of consent obtained (written/oral) or the reason that consent was not obtained (e.g. the data were analyzed anonymously)<br><br>**Animal Research (involving vertebrate animals, embryos or tissues)**<br>• Provide the name of the Institutional Animal Care and Use Committee (IACUC) or other relevant ethics board that reviewed the study protocol, and indicate whether they approved this research or granted a formal waiver of ethical approval<br>• Include an approval number if one was obtained<br>• If the study involved *non-human primates*, add *additional details* about animal welfare and steps taken to ameliorate suffering<br>• If anesthesia, euthanasia, or any kind of animal sacrifice is part of the study, include briefly which substances and/or methods were applied<br><br>**Field Research**<br><br>Include the following details if this study involves the collection of plant, animal, or other materials from a natural setting:<br>• Field permit number<br>• Name of the institution or relevant body that granted permission | |
| **Data Availability**<br><br>Authors are required to make all data underlying the findings described fully available, without restriction, and from the time of publication. PLOS allows rare exceptions to address legal and ethical concerns. See the PLOS Data Policy and FAQ for detailed information. | Yes - all data are fully available without restriction |

| | |
|---|---|
| A Data Availability Statement describing where the data can be found is required at submission. Your answers to this question constitute the Data Availability Statement and **will be published in the article**, if accepted.<br><br>**Important:** Stating 'data available on request from the author' is not sufficient. If your data are only available upon request, select 'No' for the first question and explain your exceptional situation in the text box.<br><br>Do the authors confirm that all data underlying the findings described in their manuscript are fully available without restriction? | |
| **Describe where the data may be found in full sentences. If you are copying our sample text, replace any instances of XXX with the appropriate details.**<br><br>• If the data are **held or will be held in a public repository**, include URLs, accession numbers or DOIs. If this information will only be available after acceptance, indicate this by ticking the box below. For example: *All XXX files are available from the XXX database (accession number(s) XXX, XXX.).*<br>• If the data are all contained **within the manuscript and/or Supporting Information files**, enter the following: *All relevant data are within the manuscript and its Supporting Information files.*<br>• If neither of these applies but you are able to provide **details of access elsewhere**, with or without limitations, please do so. For example:<br><br>*Data cannot be shared publicly because of [XXX]. Data are available from the XXX Institutional Data Access / Ethics Committee (contact via XXX) for researchers who meet the criteria for access to confidential data.*<br><br>*The data underlying the results presented in the study are available from (include the name of the third party* | All relevant data are within the manuscript and its Supporting Information files. |

Powered by Editorial Manager® and ProduXion Manager® from Aries Systems Corporation

| | |
|---|---|
| *and contact information or URL).*<br>• This text is appropriate if the data are owned by a third party and authors do not have permission to share the data.<br><br>* typeset | |
| Additional data availability information: | Tick here if your circumstances are not covered by the questions above and you need the journal's help to make your data available. |

Powered by Editorial Manager® and ProduXion Manager® from Aries Systems Corporation

Revised Manuscript Click here to access/download;Manuscript;Revised Manuscript.docx

# Full-title: The immunoglobulin G antibody response to malaria merozoite antigens in asymptomatic children co-infected with malaria and intestinal parasites

Running title: Malaria antibodies in children with intestinal parasites

Authors: Crespo'o M. Ndiabamoh[1,2*], Gabriel L. Ekali[2,5], Livo Esemu[2,3], Yukie M. Lloyd[4] Wilfred Mbacham[1,2,3], Jude D. Bigoga[2,3] Diane W. Taylor,[4] and Rose G.F. Leke[2*]

[1]Faculty of Medicine and Biomedical Sciences, University of Yaoundé I, Cameroon

[2]The Biotechnology Center, Nkolbisson, University of Yaoundé I, Cameroon

[3]Department of Biochemistry, University of Yaoundé 1, Cameroon

[4]John A. Burns School of Medicine, University of Hawai'i at Mānoa, USA

[5]National AIDS Control Committee, Ministry of Public Health, Cameroon

*Correspondence to Rose Gana Fomban Leke (roseleke@yahoo.com)(RGFL) and Crespo'o Mbe-cho Ndiabamoh (cndiabamoh@gmail.com) (CMN).

**Comments:**
**1. Check carefully all the italics**
**2. Line 342 remove italics**
**3. As I previously commented, the discussion about MSP1, MSP2, and MPS3 proteins is poor and is not comparing data of other studies using the same proteins.**
**4. Mention in results that hookworms were not detected in the study**
**5. Mention in results that no significant difference was observed between the malaria antibodies levels and parasites eggs counts.**
**6. Although the authors specify two references describing the bead assay, I still believe it is important to report each antigen's concentration in the bead assay. Once the authors explained that these concentrations are not comparable. Additionally, in the references given (old 23 and 24), there is no information about how EBA175 was produced.**
**7. Please add references showing that eosinophilia is maintained in individuals that had intestinal infections during a longer time even after MDA.**
**8. The author explains that "The fact that Intestinal parasite (IP) infections was only observed in children >2 years, helped guide separation of the children into seven groups." But that is not e real explanation; please explain what the rationale for that separation is? Why not use 3-4-5-6 groups?**
**9. Please comment in the abstract that the population received mass drug administration. I consider that that is a crucial point of the investigation and explains the lower prevalence of helminths and protozoans.**

# ABSTRACT

## Background

Co-infection with malaria and intestinal parasites is common in children in Africa and may affect their immune response to a malaria parasite infection. Prior studies suggest that co-infections may lead to increased susceptibility to malaria infection and disease severity; however, other studies have shown the reverse. Knowledge on how co-morbidities specifically affect the immune response to malaria antigens is limited. Therefore, this study sought to determine the prevalence of co-infection of malaria and intestinal parasites and its association with antibody levels to malaria merozoite antigens.

## Methods

A cross sectional study was carried out in two villages with high transmission of malaria in Cameroon (Ngali II and Mfou). Children aged 1-15 years were enrolled after obtaining parental consent. A malaria rapid diagnostic test was used on site. Four (4) ml of peripheral blood was collected from each participant to determine *Plasmodium falciparum* infections by microscopy, haemoglobin levels and serology. Fresh stool samples were collected and examined by wet mount, Kato-Katz method and modified Ritchie concentration techniques. A Multiplex Analyte Platform assay was used to measure antibody levels.

## Results

A total of 320 children were enrolled. The prevalence of malaria by blood smear was 76.3% (244/230 and prevalence of malaria and intestinal parasites was 16.9% (54/320). Malaria prevalence was highest in young children; whereas, intestinal parasites (IP+) were not present until after 3 years of age. All children positive for malaria had antibodies to $MSP1_{42}$, MSP2, MSP3 and EBA175. No difference in antibody levels in children with malaria-co infections compared to malaria alone were found, except for antibody levels to EBA-175

were higher in children co-infected with intestinal protozoa (p = 0.018), especially those with *Entamoeba histolytica* infections (p=0.0026).

## Conclusion

Antibody levels to EBA175 were significantly higher in children co-infected with malaria and *E. histolytica* compared to children infected with malaria alone. It is important to further investigate why and how the presence of these protozoans can modulate the immune response to malaria antigens.

**Key words**: malaria, intestinal-parasites, antibody, *Giardia lamblia, Entamoeba histolytica*

# Introduction:

In sub-Saharan Africa, malaria caused by *Plasmodium falciparum* (Pf) remains an important public health threat, killing over 271,000 children under the age of five each year (1). In malaria endemic areas, individuals exposed to malaria infections gradually develop clinical immunity (2) and commonly experience asymptomatic infections without fever or symptoms and do not require antimalarial treatment. Asymptomatic infection results from partial immunity that controls, but does not completely eliminate, malaria parasites, thus allowing for constant presence of circulating parasites (2).

The prevalence of intestinal parasitic infections in children is fairly constant across sub-Saharan Africa with an average prevalence of 26% (3,4). In Cameroon, the prevalence in children less than 18 years is 26.8% (5), while that for the general population is more than 28% The major intestinal parasites are Ascaris *lumbricoides, Trichuria trichuria and Entamoeba histolytica* (6–8), but many cases of intestinal parasites go undetected.

Co-infections with malaria and intestinal parasites (IP) are common in malaria endemic areas in sub-Saharan Africa (7,8) and infections with IP and Pf are both ranked among the major cause of mortality and morbidity in sub-Saharan Africa. Several studies conducted on IP (not including amoebas) and Pf have shown conflicting results. Some helminths suppress different T-helper types and favor an increase in regulatory T (Treg) cell (9). Studies on concomitant infections in humans suggest that *A. lumbricoides* infection may protect against cerebral malaria (10,11), while other studies suggest that children infected by *Schistosoma mansoni may be* more susceptible to *P. falciparum* infections and develop acute malaria episodes (12,13). Also, it has been shown that the levels of TNF-α, IL-2, IL-10, IL-6 in *Plasmodium*-helminth co-infected individuals were significantly higher than the malaria-positive (MP) group (14) dampening the immune response to malaria. However, little is known regarding host immune responses to malaria in children co-infected with protozoan pathogens.

Studies suggest that children co-infected with malaria and intestinal helminths had significantly decreased antibody levels to the malarial antigen apical merozoite antigen 1 (AMA-1) compared to those with *P. falciparum* or IP alone(15). Hence, infections with intestinal helminths can stifle protective anti-plasmodial antibody responses (15). However, increase in MSP3 IgG1–4 levels were significantly associated with children infected with malaria alone compared to children co-infected with both parasites(15).

Malaria and other intestinal parasites overlap extensively in their epidemiological distributions causing polyparasitism. Polyparasitism with intestinal parasites has been reported as one of the contributing factors to hypo-responsiveness (16), dampening of the immune response by inducing a strong Treg response, which could in turn, blunt a strong response to vaccines (17). Equally, some studies have suggested an effect of IP on antibody responses to P. falciparum gametocyte antigens that may have consequences on transmission-blocking immunity (18).

Effective elimination and future eradication of malaria will require not only vector control, but also managing asymptomatic malaria patients and developing an effective vaccine. Given the high burden and concomitant nature of both malaria and intestinal parasites in the same geographical setting, conflicting data shows polyparasitism could interfere with the efficacy of malaria vaccines (19). To our knowledge, since limited information is available on whether and how co-infections of intestinal parasites and malaria affect the specific immune response to malaria antigens (20), the goal of this study was to investigate the prevalence and relationship between co-infections of malaria (MAL+) and intestinal parasites (IP+) (nematodes, trematodes, and protozoans) on naturally acquired antibodies to malaria merozoite .

# Methods

## Study area description

The study was conducted in Ngali II and Mfou, two villages in the central region of Cameroon (located at 4°27′N and 11°38′E) with a total population of about 1,000 children per squared Km (about 4000 in Ngali II and 6000 in Mfou) under the age of 15 years. The climate is typically equatorial with two discontinuous dry and rainy seasons. The annual average rainfall measures about 1600 $mm^3$ with an annual average temperature of 23°C (21).

Most children in Ngali II and Mfou over 3 years of age accompany their parents to the farm and return home late at night. The use of mosquito bed nets is rare in the two villages and residents have minimal access to portable water with approximately one well per 500 inhabitants.  Currently, mass drug administration with albendazole is being performed twice a year by the Ministry of Health, that is usually conduced in schools and symptomatic cases are sent to the local clinic or hospital for follow up treatment.

## Study population

A cross sectional study was carried out in Ngali II and Mfou from January to May 2017, a transitional period from the dry to wet season. Children who had lived in either of the villages for at least six months and whose parents gave informed consent were included in the study. All participants were systematically examined by a physician for clinical systems of malaria and IP. Children who presented with symptoms of malaria, e.g., fever, headaches or intestinal illnesses, e.g., diarrhea, vomiting were not enrolled. A total of 320 participants (140 from Ngali II and 180 from Mfou) aged 1-15 years participated in the study. Since both villages have the same demographic features, data for the two villages were combined.

## Blood collection and on-site testing for malaria

Venous peripheral blood (about 4mL) was collected by venipuncture using a butterfly needle (G22) and a 5mL labeled EDTA tube from all 320 participants. Haemoglobin (Hb) was measured using the HemoCue (AB Leo Diagnostics, Helsingborg, Sweden). On site, after collecting the venous blood from the participants, a drop from the same collected blood was placed on a CareStart$^{TM}$ Malaria pLDH/HRP-2 Combo Test (Access Bio Inc. USA) to detect histidine-rich protein-2 (HRP-2) specific to *Plasmodium falciparum* and Plasmodium lactate dehydrogenase (pLDH) pan-specific to *Plasmodium* spp (*falciparum, P. vivax, P. malariae, P. ovale*). Results were read according to manufacturer instructions and recorded after 5 minutes.

## Laboratory detection of malaria parasites

Ten microliters of whole blood were used to prepare thick and thin smears for malaria parasite identification, speciation and quantification. The slides were air-dried overnight, and the thin blood smears were fixed in absolute (100%) methanol. Both thick and thin smears were stained using 10% Giemsa solution, washed with water and air-dried. Slides were then microscopically examined (thin and thick smear) for the presence of malaria parasites by two

experienced microscopists. The parasite density was determined by counting the number of parasites against 200 leucocytes. The counts were expressed as the number of P. falciparum-infected erythrocytes (IE) per microliter of blood (Pf IE/µl), assuming an average leukocyte count of 8,000 cells/µl of blood (22). When the difference in parasitaemia between the two readers was greater than 5 Pf IE/µl of blood, a third reader re-examined the slide and the mean of the two closest values were considered. Also, a differential count for eosinophil, lymphocytes, monocytes, neutrophils was obtained alongside parasitaemia and different malaria species (by microscopy)

## Antibody Analysis

Plasma samples were tested for antibodies against the merozoite antigens MSP-$1_{42}$, MSP-2, MSP-3 and EBA-175 using a multi-analyte platform assay with antigen-coupled magnetic beads with different spectral addresses. Details of this assay used has been described previously (23) (24). In brief, plasma samples were diluted 1:100 with PBS, 50µl of plasma was incubated with 50µl antigen-coupled microspheres (2000 microspheres/test) for 60 minutes in the dark, washed with PBS, and then incubated at 500rpm for 60minutes at 25 °C on a rotating shaker and using a magnet plate separator. Then, 100 µl of secondary Ab (R-phycoerythrin-conjugated, Affini Pure $F(ab')_2$ fragment, Goat anti-human IgG Fc fragment specific, Jackson Immuno-research, West Grove, PA, USA, Cat no. 109-116-170) diluted to 2 µg/ml in PBS-1 % BSA was added to each well and incubated as above in the dark for 1 h. The mixture is then washed and a minimum of 100 beads were read in a MAGPIX® reader. A minimum bead count of 100 per spectral address recorded as Median Fluorescence Intensity (MFI).

Controls included on each plate were: PBS to determine background fluorescence, the negative control (NC) consisted of pooled plasma from four malaria-naïve US individuals, and the positive control (PC) was pooled plasma from Cameroonians with high antibody levels to *Plasmodium* falciparum. Results were exported to Excel for analysis. The cut-off for

positivity was calculated as mean of MFI +3 standard deviation of the negative control as shown in the results sections.

## Stool sample collection and analysis

Sterile labelled stool collection vials were given to the parents along with instructions for proper stool collection. All samples were analyzed within 7 hours of collection to avoid missing hookworm eggs and minimize chances of under reporting. Approximately, 4 mg of feces was suspended in 5ml PBS and a drop examine by wet mount. The Kato Katz technique was used for morphological identification of helminths eggs, e.g., *A. lumbricoides, T. trichiura, or* larval stage of *Strongyloides stercoralis* (25) while the modified Ritchie's concentration stool technique was used to identify all protozoans and cestodes (26). The smears were read at objective 10X for eggs and larvae and objective 40X for cysts and vegetative forms of protozoan.   All stool slides were read by 2 technicians and in 2 different laboratories under supervision of a microbiologist and parasitologists.

## Data analysis

Data were analyzed using Microsoft Excel 2013, and GraphPad® prism 8. Standard summary statistics were used to describe the study population and results are presented as proportions. Fischer's exact test was used to compare antibody levels between the malaria-negative, IP-positive (MAL-,IP+) and malaria-positive, IP-negative (MAL+,IP-) groups, because of the small sample sizes of the groups. The one-way-ANOVA test was used to compare all 4 groups after checking for normality (e.g., age). An unpaired t test was used to compare the means of the MAL-,IP- vs. MAL+,IP- groups. Kruskal-Wallis test was used to compare antibody levels, which are not normally distributed, among the groups or within the MAL+IP+ groups. An individual was considered to have a co-infection if at least one IP species and *P. falciparum* were present. Anaemia was considered when Hb values were < 11.5 g/dL and classified according to WHO (27,28).  To search DNA sequences of *P.*

*falciparum* EBA-175 and those of *E. histolytica* for possible cross-reactive epitopes, PfEBA175 (ncbi.nlm.nih.gov/gene/2654998) was compared with E. histolytica (ncbi.nlm.nih.gov/assembly/GCF_000208925.1) using Megablast for highly similar sequences and discontinuous megablast for more dissimilar sequences.

# Results

## The study population

A total of 320 children were enrolled (Table 1). Among the children, 76.3% were slide-positive for malaria (MAL+), with 59.4% having malaria without intestinal parasites (MAL+,IP) and 16.9% being coinfected with malaria and intestinal parasites (MAL+, IP+). All subjects who tested positive for malaria using the rapid diagnostic field test were confirmed positive by microscopy. Among children who were infected with malaria, 71.3% were infected with only *P. falciparum* and 5% had *P. falciparum* and *P. malariae*. Interestingly, only 2.2% of the children had IP without malaria and 21.6% were negative for both malaria and IP.

The mean age of the children changed with infection status among the 4 groups (p = 0.0001) with the lowest age found in uninfected children (6.4 years) and highest in children with co-infections (9.3 years) (Table 1). Malaria infections were found in all age groups; whereas, none of the children under age 4 years had intestinal parasites. Mean haemoglobin levels were lower in children infected with malaria, but the difference was of marginal significance (p = 0.08; MAL-,IP- vs MAL+,IP-). The prevalence of anaemia was higher in children who were infected with malaria (MAL+,IP-)(p=0.032), but not those with co-infections (p >0.999) compared to children who were parasite-negative (MAL-,IP-).

Table 1: Description of 320 children infected with malaria and intestinal parasites (IP)

| | MAL-, IP- | MAL+ IP- | MAL-,IP+ | Co-infections (Mal+,IP+) | P values |
|---|---|---|---|---|---|

| | | | | | |
|---|---|---|---|---|---|
| Number (%) of children | 69 (21.6) | 190 (59.4) | 7 (2.2) | 54 (16.9) | |
| Mean years of age (range) | 6.4 (1-14) | 7.9 (1-15) | 8.6(4-12) | 9.3(4-15) | 0.0001* |
| Parasitaemia: (median # infected erythrocytes/µl (range) | 0 | 420 (40-96,000) | 0 | 900 (40 –30,970 | 0.1599** |
| Measures of anaemia | | | | | |
| Hb (g/dL) (mean ±SD) | 12.1 ±1.6 | 11.6 ± 2.2 | 12.2 ± 1.4 | 12.4±1.8 | 0.0658* |
| Prevalence of anaemia | | | | | |
| # (%) of children with Hb <11.5 g/dL | 21 (30.4) | 87 (45.8) | 2 (28.6) | 17 (31.5) | 0.0324*** |

*comparison among the 4 groups (ordinary one-way ANOVA)

** comparison among the 4 groups (Mann-Whitney test)

*** comparison between MAL-,IP- vs. MAL+,IP- (Fisher's exact test)

# Prevalence of intestinal parasites

Overall, 19.1% (61/320) of the children were positive for intestinal parasites, 16.9% of whom were also infected with malaria and 2.2% were IP+ but MAL- (Table 2). The most frequent helminthic parasites detected were *A. lumbrioides* (2.8%) and single cases of *Trichura sp*. and *Strongyloides sp.* Among the 320 children, 14.7% had detectable protozoan infections, including 7.8% infected with *Giardia lamblia*, 5.9% with *E. histolytica*, and 0.9% with *Isospora sp*. Very few children had intestinal cestodes (Table 2). Interestingly, all of the children had single parasite infections, and polyparasitism was not found.

Table 2: Prevalence of Intestinal Parasites (IP+) in the 320 Children, Ages 1 to 15 years

| | Number of Children | | |
|---|---|---|---|
| | MAL-, IP+ | MAL+, IP+ | Total IP+ (% positive) |
| Intestinal Parasites | | | |
| **Helminths** | | | **11 (3.4%)** |
| *Ascaris lumbricoides* | 2 | 7 | 9 (2.8%) |
| Others* | 0 | 2 | 2 (0.87%) |
| **Protozoans** | | | **48 (14.7%)** |
| *Giradia lamblia* | 3 | 22 | 25 (7.8%) |
| *Entamoeba histolytica complex* | 1 | 18 | 19 (5.9%) |
| Other** | 1 | 3 | 4 (0.9%) |
| **Cestodes** | | | **2 (0.63%)** |
| *Hymenolepis nana* | 0 | 2 | 2 (0.63%) |
| **Total IP** | **7** (2.2%) | **54** (16.9%) | **61** (19.1%) |

Others*: 1 *Trichura sp*. and 1 *Strongyloides sp.*

Others**: 3 *Isospora sp*.

## Influence of age on malaria, intestinal parasites, anaemia and moderate eosinophilia

As expected, children aged 1 through 2 years did not have soil-transmitted IP and had normal eosinophil levels; whereas, 63% of 1-2-year old children were infected with malaria and had the highest prevalence of anaemia (Table 3). In contrast, in children 9-15 years of

age ~80% were slide-positive for malarial parasites, 24%-29% had intestinal parasites, and 10-38% had moderate eosinophilia. Thus, as children living in these villages increased with age, they began developing partial immunity to malaria symptoms and anaemia declined; whereas, the prevalence of IP and eosinophilia increased.

Table 3: Influence of Age on Malaria, Intestinal Parasites, Anaemia and Percentage of Peripheral Eosinophils

| Age (years) | N = | % Mal+ | % IP+ | * % with anaemia | **% with eosinophilia |
|---|---|---|---|---|---|
| 1 - 2 | 27 | 63.0 | 0 | 55.6 | 0 |
| 3 - 4 | 47 | 61.7 | 6.4 | 48.9 | 4.3 |
| 5-6 | 40 | 62.5 | 15.0 | 40.0 | 7.5 |
| 7-8 | 63 | 88.9 | 28.6 | 36.5 | 9.5 |
| 9-10 | 55 | 83.6 | 21.8 | 38.2 | 20.0 |
| 11-12 | 54 | 79.6 | 25.9 | 38.9 | 22.2 |
| 13-15 | 34 | 82.4 | 23.5 | 26.5 | 38.3 |

*Aneamia: Children with haemoglobin less than 11.5 g/dL. **Moderate eosinophilia: ≥1,500 eosinophils/mm$^3$ or ≥18.7% peripheral eosinophils

A comparison of anaemia and eosinophilia among the 4 groups of children shown in Table 1 was made (S1 Table). Results showed that anaemia was associated with malaria infections and eosinophilia was associated with IP.

# Antibody Levels to Malaria Merozoite Antigens

With repeated exposure to malaria, Ab prevalence and levels increased with age to the four merozoite antigens (Fig 1). Among 1- to 2-year-olds, only 25% of the infants had Ab to EBA-175 and MSP3, 30% had Ab to MSP2, but 80% had Ab to MSP1 (Fig 1). However, by age 13-15 years, 60% had acquired Ab to MSP3 and >80% had Ab EBA-175, MSP2 and MSP3 (Fig 1A). Among Ab-positive children, Ab levels also increased with age (Fig 1B-E).

Although different amounts of Ab were ultimately obtained to the different antigens, the overall trend was for an increase in median Ab with age. Thus, it was important to take age into consideration when making comparison between children infected with malaria (MAL+,IP-), co-infected with malaria and IP (MAL+,IP+) and those who were not infected (MAL-,IP-) at the time the study was conducted.

[Insert Figure 1]

**Fig1: Prevalence and amount of Ab in different age groups**. (A) Prevalence of Ab to the 4 merozoite antigens. The number of participants in each age group is provided in Table 3. Fig1 B – E show Ab levels (MFI) for children who were Ab-positive for each age group. Horizontal bars represent median Ab levels. Kruskal-Wallis test (nonparametric comparison among groups) values were for MSP1 ($p=0.067$); MSP2 ($p<0.001$); MSP3 ($p=0.086$) and EBA ($p=0.056$). MFI = Median fluorescence intensity; MSP = merozoite surface proteins; EBA= erythrocytes binding antigen

# Comparison of Ab levels in participants with and without malaria and IP

Since children below 3 years of age were not infected with IP, they were not included in the comparative studies described below. Given that Ab prevalence and levels increased with age, the study population was divided into 2 groups: children aged 3-10 years, a time period when children were becoming infected with IP (Table 3) and those 11-15 years, mainly children who had been infected repeatedly with malaria and may had lived with IP for a period of time. As predicted, Ab levels were slightly higher in MAL+ children due to current boosting compared to MAL-, but the differences were not significant (all p values >0.05) (Fig 2).

A comparison between Ab levels in children infected with malaria and co-infected with IP was conducted. Children with helminths and cestodes were not included in the

analysis because the sample sizes were too small. Ab levels were compared between children aged 3-10 years infected with malaria (n=112) and co-infected with flagellate and intestinal amoeba (n= 25 children), including *G. lamblia* (n= 15) and *E. histolytica* (n = 10 children) (Fig 2). Antibody levels did not differ between malaria-infected children with or without intestinal amoeba for MSP1, MSP2 and MSP3; however, there were significantly higher Ab levels to EBA-175 in children co-infected with malaria and intestinal amoeba (p = 0.018) (Fig 2D). The higher Ab levels were due to *E. histolytica* infections (p=0.0026), and not *G. lamblia* (p=0.3844). No differences were found between children aged 11 to 15 years for any of the antigens between children with malaria (single infection) and co-infected with any of the IP.

To determine if higher Ab levels in children co-infected with *P. falciparum* and *E. histolytica* might be due to cross-reactive epitopes, a BLAST search for sequence homology between EBA-175 and *E. histolytica* proteins. No similarities were found using Metablast, and only one hit was found using discontinuous metablast which had a span of only 38 nucleotides (~13 amino acids) that had 82% similarity. Thus, there does not appear to be shared epitopes between these two pathogens that would explain the increase in Ab to EBA-175 in children with co-infections.

[Insert Figure 2]

**Fig 2. Antibody levels in children ages 3 to 10 for all antibody-positive individuals**
Distribution of Ab levels in MFI among malaria negative (MAL-) and malaria-positive (MAL+) and those co-infected with malaria plus Intestinal (Int.) protozoa (n=25); malaria plus *G. lamblia* (n=15); and malaria plus *E. histolytica* (n=10). The number of datapoints varied because not all participants had Ab to all antigens. Horizontal lines represent medians for the group. MFI = median florescence intensity; MSP = merozoite surface proteins, EBA = erythrocytes binding antigen (EBA)

# Discussion

Malaria and polyparasitism (cestodes, protozoans, trematodes) are still common conditions throughout Africa (29,30). In the 1-15-year-old children living in the rural Cameroonian villages surveyed, the prevalence of slide-positive malaria was 76.3% and 19.1% had intestinal parasites, with 16.9% co-infections (Table 1-2). This prevalence of malaria is similar to those found in other highly endemic regions of the country (31), and the prevalence of co-infections was 19.1%, which is similar to a prevalence of 18 – 27% reported in other regions of Cameroon (32,33). This high transmission is related to geo-ecological and climatic conditions at the time of the study which was the transition from the dry to wet season, a period that favors vector breeding and distribution (34).

From Table 3, the prevalence of slide-positive malaria ranged from 61% to 90% in different age groups implying that children in these villages were repeatedly exposed to malaria throughout their lives. The current prevalence of malaria in 2017 is similar to that recorded previously for Ngali II between 1998-2004, that ranged from 50% to 85% in 5-15 year old children with an estimated entomological inoculation rate of 0.7 infectious bites/per/night (~257 infectious bites annually)(35). Prior studies have established that repeated exposure induces immunity to malaria, with development of anti-disease immunity followed by anti-parasite immunity (36–39).  As a result, the highest prevalence of 56% anaemia was found in young children (2,40,41) with a decline to 27% in 13 to 15-year-olds (Table 3). On the other hand, Infections with IP only occurred later in life from 3 years onward with a mean infection age of 8.1 years. Increase in intestinal parasites was associated with an age-related increase in eosinophil counts (42,43), a known innate immune response to helminthic and other soil-transmitted organisms (Table 3).  In this study, only 11/320 (3.4%) children were infected with helminths. Although some epidemiological studies have demonstrated an increased risk of infection by *P. falciparum* in individuals co-infected with helminths, other results are conflicting (44,45). The low prevalence of helminths is explained, in part by, the

fact that mass community de-worming is done biannually following the national infectious disease guide-line for IP control program. The most prevalent intestinal parasites were the protozoans, *G. lamblia* and *E. histolytica* (48). These protozoa are commonly found in damp soil and contaminated water with a prevalence of 2-20% in Cameroon (50–53). These results suggest children acquire their intestinal infections after learning to walk and interact with the environment. Thus, children in the study population were exposed to malaria early in life and began developing anti-malaria immunity prior to exposure to intestinal parasites.

Generally, both Ab prevalence and Ab levels increased with age in 1 to 15-year-olds living in this high transmission area (Fig 1). Often, the presence of Ab is used as markers of infection, including the merozoite antigens used in this study. This study compared antibody levels with age in four main groups of children, MAL+,IP+, MAL-,IP-, MAL,-IP+ and MAL+,IP- children to four (MSP1, MSP2 MSP3, EBA17) malaria antigens (54–56). Since over 80% of 1-2-year-olds had Ab to MSP1, humoral immunity began to develop early in life and continued to mature as children developed into adolescents (Fig 1). Often individuals who are MAL+ have higher Ab levels than MAL- individuals due to boosting of the Ab response (36,38,39). In the current study, Ab levels did not differ significantly between MAL+ and MAL- individuals, neither those who were 3-10 years nor 11-15 years-old. This result was not surprising, since 75% of the children were slide-positive for malaria (Table 1). Because of high transmission, children are becoming infected almost on a daily basis and either are in the process of eliminating the new infection or reducing it to submicroscopic levels. Thus, most children living in areas with high perennial transmission will test positive for malaria by PCR. *Because of constant re-exposure, the resulting immune response will be similar to that produced by a chronic infection.*

Prior studies have demonstrated that malaria-helminths co-infections can down regulate malaria and orient the immune response via the Th2 response hence, making patients less sick (20,36,57,58) whereas, others have demonstrated on the contrary that IP and malaria co-infections increase malaria disease (13,56). Unfortunately, the current study could not

resolve the controversy because very few children had helminthic infections, due to frequent treatment with albendazole via the mass drug administration program conducted by the Ministry of Health and other random health campaigns. However, co-infections with malaria and amoeba were relatively common. Ab levels to MSP1, MSP2 and MSP3 were similar in children infected with *P. falciparum* alone and those with amoeba (Fig 2); however, significantly higher Ab levels to EBA-175 were found in children co-infected with malaria and intestinal amoeba (p = 0.018). The higher Ab levels were due to *E. histolytica* infections (p = 0.0026), and not *G. lamblia* (p = 0.384). This result was unexpected. *E. histolytica* is a gut amoeba that cause both intestinal and extraintestinal infections such as amebic colitis (dysentery) and liver or brain abscess. This protozoa can cause a marked down-regulation of macrophage functions rendering the cells incapable of antigen presentation and unresponsive to cytokine stimulation (59). This decrease in macrophage function does not explain the increase in Ab to EBA-175. One possible explanation is that since malaria and *E. histolytica* are both protozoan pathogens, they might share common antigens, for example, EBA-175 could share homology with an *E. histolytica* antigen. To investigate this possibility, a blast search of the NCIB gene bank was conducted for EBA-175 and the *E. histolytica* genome. However, this search revealed only a ~13 amino acid sequence with 82% similarity, which is clear too small to explain the increase in Ab levels of co-infected children. Finally, an alternative explanation could be that this result is a spurious observation by chance. Clearly the association between malaria and *E. histolytica* merits further study.

Altogether, a keen observation needs to be repeated with a larger sample size as *E. histolytica* boosting of Ab to EBA-175 – co-infection might not only be limited to EBA-175, but other antigens as well. Children in these villages began to acquire an Ab response to the 4 merozoite antigens early in life, prior to infection with IP. There was no evidence that infection with IP influenced Ab levels or negatively-altered the already established Ab response to the 4 merozoite antigens.

# CONCLUSION

The prevalence of malaria was high in children 1-2 years old; whereas, intestinal parasite infections occurred in children over 3 years old. Thus, immunity to *P. falciparum* began prior to infection with soil-transmitted parasites. No differences were found in antibody prevalence or levels in malaria-infected and co-infected children, except antibody levels to EBA175 were significantly higher in children co-infected with malaria and *E. histolytica*. This is the first report of an interaction between malaria and *E. histolytica* and antibodies to EBA-175 and merits further evaluation.

# Declarations

## Ethical consideration

Ethical clearance used for the study was obtained from the Cameroon National Ethics Committee (IRB approval: Nº2016/12/845/CE/CNERSH/SP). Administrative authorizations were obtained from authorities of the Ngali II and Mfou health districts.

Informed consents were obtained from parents of all participants. A clinical examination was performed for all eligible participants by a medical doctor.

All participants positive for any *Plasmodium s*pp by RDT at the time of blood collection and those who were found to have PI by stool analysis were treated for free following the protocol recommended by the Cameroonian Ministry of Health. All children with mild anaemia were given an iron supplement free of charge.

## Authors' contributions

GFLR supervised the study. JDB and WM co-supervised the study. CMN and ELG conceived and designed the work. CMN, EFL, DJC, AEW, MBN carried out experiments.

Data was collected and analyzed by CMN. The first draft of this manuscript was written by CMN, critically read and edited by DWT, YML. DWT and GFLR reviewed the final draft.


## Acknowledgements

We heartily thank all the participants and their parents who let them participate in the study in Ngali II and Mfou. We equally express our gratitude to the community health workers of these areas for their assistance during sample collection.

Anna Babakhanyan, of Hawaii university provided the recombinant proteins (beads) used for the assays. The authors acknowledge the support of the entire staff of the Biotechnology Centre of University of Yaoundé I, Cameroon for their tremendous work in the field during sample collection and sample processing in the lab.

## Funding statement

The Luminex MAGpix was provided by grant P30GM11473, Centers of Biomedical Research Excellence, National Institute of General Medical Sciences, NIH. GFLR provided the funds for the field work and analysis.


## Competing interest

Authors declare no competing interests.

## Data Availability:

The database for the study can be found in the “Supporting Material File.”

## Consent for publication

All authors give their consent for publication of this manuscript.

## References

1. World-Malaria-Report-2019-briefing-kit-eng.pdf [Internet]. [cited 2020 Jul 6]. Available from: https://www.who.int/malaria/publications/world-malaria-report-2019/World-Malaria-Report-2019-briefing-kit-eng.pdf

2. Chen I, Clarke SE, Gosling R, Hamainza B, Killeen G, Magill A, et al. "Asymptomatic" Malaria: A Chronic and Debilitating Infection That Should Be Treated. PLOS Med. 2016 Jan 19;13(1):e1001942.

3. Pampiglione S, Visconti S, Pezzino G. [Human intestinal parasites in Subsaharan Africa. II. Sao Tomé and Principe]. Parassitologia. 1987 Apr;29(1):15–25.

4. Fletcher SM, Stark D, Ellis J. Prevalence of gastrointestinal pathogens in Sub-Saharan Africa: systematic review and meta-analysis. J Public Health Afr [Internet]. 2011 Sep 5 [cited 2018 Sep 9];2(2). Available from: https://www.ncbi.nlm.nih.gov/pmc/articles/PMC5345503/

5. Fusi-Ngwa C, Besong E, Pone JW, Mbida M. A Cross-Sectional Study of Intestinal Parasitic Infections in Children in Ghettoed, Diverse and Affluent Communities in Dschang, West Region, Cameroon. Open Access Libr J. 2014 Dec 1;01:1.

6. Mwangi TW, Bethony JM, Brooker S. Malaria and helminth interactions in humans: an epidemiological viewpoint. Ann Trop Med Parasitol. 2006 Oct;100(7):551–70.

7. Zeukeng F, Tchinda VHM, Bigoga JD, Seumen CHT, Ndzi ES, Abonweh G, et al. Co-infections of Malaria and Geohelminthiasis in Two Rural Communities of Nkassomo and Vian in the Mfou Health District, Cameroon. PLoS Negl Trop Dis [Internet]. 2014 Oct 16 [cited 2016 Dec 5];8(10). Available from: http://www.ncbi.nlm.nih.gov/pmc/articles/PMC4199518/

8. Njunda AL, Fon SG, Assob JCN, Nsagha DS, Kwenti TDB, Kwenti TE. Coinfection with malaria and intestinal parasites, and its association with anaemia in children in Cameroon. Infect Dis Poverty [Internet]. 2015 Oct 6 [cited 2016 Dec 8];4. Available from: http://www.ncbi.nlm.nih.gov/pmc/articles/PMC4595138/

9. Hopkin J. Immune and genetic aspects of asthma, allergy and parasitic worm infections: evolutionary links. Parasite Immunol. 2009;31(5):267–73.

10. Nacher M, Gay F, Singhasivanon P, Krudsood S, Treeprasertsuk S, Mazier D, et al. *Ascaris lumbricoides* infection is associated with protection from cerebral malaria. Parasite Immunol. 2000 Mar 1;22(3):107–13.

11. Brutus L, Watier L, Hanitrasoamampionona V, Razanatsoarilala H, Cot M. Confirmation of the protective effect of Ascaris lumbricoides on *Plasmodium falciparum* infection: results of a randomized trial in Madagascar. Am J Trop Med Hyg. 2007 Dec;77(6):1091–5.

12. Author(S): M. Nacher, P. Singhasivanon, S. Yimsamran, W. Manibunyong, N. Thanyavanich, P., Wuthisen, And S. Looareesuwan, Author(S): M. Nacher, P. Singhasivanon, S. Yimsamran, W. Manibunyong, N. Thanyavanich, P. Intestinal helminth infections are associated with increased incidence of *Plasmodium falciparum* almaria in Thailand [Internet]. [Cited 2017 May 1]. Available From: Http://Sci-Hub.Cc/Http://Dx.Doi.Org/10.1645/0022-3395(2002)088[0055:Ihiaaw]2.0.Co;2

13. Sokhna C, Le Hesran J-Y, Mbaye PA, Akiana J, Camara P, Diop M, et al. Increase of malaria attacks among children presenting concomitant infection by *Schistosoma mansoni* in Senegal. Malar J. 2004;3:43.

14. Lo AC, Faye B, Gyan BA, Amoah LE. Plasmodium and intestinal parasite perturbations of the infected host's inflammatory responses: a systematic review. Parasit Vectors [Internet]. 2018 Jul 3 [cited 2019 Sep 16];11. Available from: https://www.ncbi.nlm.nih.gov/pmc/articles/PMC6031113/

15. Njua-Yafi C, Nkuo-Akenji T, Anchang-Kimbi J, Apinjoh T, Mugri R, Chi H, et al. The Effect of Helminth Co-Infection on Malaria-Specific Immunoglobulin G Responses. BMJ Glob Health. 2017 Feb 1;2(Suppl 2):A66–A66.

16. Nacher M. Malaria vaccine trials in a wormy world. Trends Parasitol. 2001 Dec 1;17(12):563–5.

17. Maizels RM, Pearce EJ, Artis D, Yazdanbakhsh M, Wynn TA. Regulation of pathogenesis and immunity in helminth infections. J Exp Med. 2009 Sep 28;206(10):2059–66.

18. Ateba-Ngoa U, Jones S, Zinsou JF, Honkpehedji J, Adegnika AA, Agobe J-CD, et al. Associations between helminth infections, *Plasmodium falciparum* parasite carriage and antibody responses to sexual and asexual stage malarial antigens. Am J Trop Med Hyg. 2016 Aug 3;95(2):394–400.

19. Hartgers FC, Yazdanbakhsh M. Co-infection of helminths and malaria: modulation of the immune responses to malaria. Parasite Immunol. 2006 Oct 1;28(10):497–506.

20. Sanchez, nchez-Arcila JC, Perce-da-Silva D de S, Vasconcelos MPA, Rodrigues-da-Silva RN, Pereira VA, et al. Intestinal Parasites Coinfection Does Not Alter Plasma Cytokines Profile Elicited in Acute Malaria in Subjects from Endemic Area of Brazil. Mediators Inflamm. 2014 Sep 16;2014:e857245.

21. Kuitcha D, Fouepe A, Ndjama J, Takem G, Awah M, Kamgang V. Chemical and isotopic signal of precipitation in Yaounde-Cameroon. Arch Appl Sci Res. 2012 Oct 15;4:2591–7.

22. District Laboratory Practice in Tropical Countries Part 1, Second Edition - monica-cheesbrough-district-laboratory-practice-in-tropical-countries-part-1.pdf [Internet]. [cited 2017 Nov 14]. Available from: https://medicallabtechno.weebly.com/uploads/7/5/1/5/7515789/monica-cheesbrough-district-laboratory-practice-in-tropical-countries-part-1.pdf

23. Fodjo BAY, Atemnkeng N, Esemu L, Yuosembom EK, Quakyi IA, Tchinda VHM, et al. Antibody responses to the full-length VAR2CSA and its DBL domains in Cameroonian children and teenagers. Malar J [Internet]. 2016 [cited 2020 Feb 8];15. Available from: https://www.ncbi.nlm.nih.gov/pmc/articles/PMC5097422/

24. Babakhanyan A, Fang R, Wey A, Salanti A, Sama G, Efundem C, et al. Comparison of the specificity of antibodies to VAR2CSA in Cameroonian multigravidae with and without placental malaria: a retrospective case–control study. Malar J. 2015 Dec 1;14(1):480.

25. Lindholz CG, Favero V, Verissimo C de M, Candido RRF, Souza RP de, Santos RR dos, et al. Study of diagnostic accuracy of Helmintex, Kato-Katz, and POC-CCA methods for diagnosing intestinal schistosomiasis in Candeal, a low intensity transmission area in northeastern Brazil. PLoS Negl Trop Dis. 2018 Mar 8;12(3):e0006274.

26. Yanet F-S, Fidel Angel N-F, Guillermo N, Sergio S-P. Comparison of parasitological techniques for the diagnosis of intestinal parasitic infections in patients with presumptive malabsorption. J Parasit Dis Off Organ Indian Soc Parasitol. 2017 Sep;41(3):718–22.

27. haemoglobin_en.doc - haemoglobin WHO .pdf [Internet]. [cited 2017 Aug 21]. Available from: http://www.who.int/vmnis/indicators/haemoglobin.pdf

28. Hemoglobin Concentration (Hb): Reference Range, Interpretation, Collection and Panels. 2016 Jun 1 [cited 2017 May 18]; Available from: http://emedicine.medscape.com/article/2085614-overview

29. Degarege A, Veledar E, Degarege D, Erko B, Nacher M, Madhivanan P. *Plasmodium falciparum* and soil-transmitted helminth co-infections among children in sub-Saharan Africa: a systematic review and meta-analysis. Parasit Vectors [Internet]. 2016 Jun 15 [cited 2020 Feb 11];9. Available from: https://www.ncbi.nlm.nih.gov/pmc/articles/PMC4908807/

30. Hürlimann E, Yapi RB, Houngbedji CA, Schmidlin T, Kouadio BA, Silué KD, et al. The epidemiology of polyparasitism and implications for morbidity in two rural communities of Côte d'Ivoire. Parasit Vectors. 2014 Feb 25;7:81.

31. Akinbo FO, Omoregie R, Mordi R, Okaka CE. Prevalence of Malaria and Anemia Among Young Children in a Tertiary Hospital in Benin City, Edo State, Nigeria. Fooyin J Health Sci. 2009 Nov 1;1(2):81–4.

32. Nkuo-Akenji TK, Chi PC, Cho JF, Ndamukong KKJ, Sumbele I. Malaria and helminth co-infection in children living in a malaria endemic setting of mount Cameroon and predictors of anemia. J Parasitol. 2006 Dec;92(6):1191–5.

33. Malaria, helminths, co-infection and anaemia in a cohort of children from Mutengene, south western Cameroon | Malaria Journal | Full Text [Internet]. [cited 2020 Jul 13]. Available from: https://malariajournal.biomedcentral.com/articles/10.1186/s12936-016-1111-2

34. Achonduh-Atijegbe OA, Mfuh KO, Mbange AHE, Chedjou JP, Taylor DW, Nerurkar VR, et al. Prevalence of malaria, typhoid, toxoplasmosis and rubella among febrile children in Cameroon. BMC Infect Dis. 2016 Nov 8;16(1):658.

35. Rf L, Jd B, J Z, Gg F, Rj L, V T, et al. Longitudinal studies of *Plasmodium falciparum* malaria in pregnant women living in a rural Cameroonian village with high perennial transmission. Am J Trop Med Hyg. 2010 Nov 1;83(5):996–1004.

36. Artavanis-Tsakonas K, Tongren Je, Riley Em. The war between the malaria parasite and the immune system: Immunity, immunoregulation and immunopathology. Clin Exp Immunol. 2003 Aug;133(2):145–52.

37. Roetynck S, Baratin M, Vivier É, Ugolini S. Cellules natural killer et immunité innée contre le paludisme. médecine/sciences. 2006 Aug 1;22(8–9):739–44.

38. Griffin JT, Hollingsworth TD, Reyburn H, Drakeley CJ, Riley EM, Ghani AC. Gradual acquisition of immunity to severe malaria with increasing exposure. Proc R Soc B Biol Sci [Internet]. 2015 Feb 22 [cited 2020 Feb 11];282(1801). Available from: https://www.ncbi.nlm.nih.gov/pmc/articles/PMC4309004/

39. Bediako Y, Adams R, Reid AJ, Valletta JJ, Ndungu FM, Sodenkamp J, et al. Repeated clinical malaria episodes are associated with modification of the immune system in children. BMC Med [Internet]. 2019 Mar 13 [cited 2020 Feb 11];17. Available from: https://www.ncbi.nlm.nih.gov/pmc/articles/PMC6415347/

40. Haldar K, Mohandas N. Malaria, erythrocytic infection, and anemia. Hematol Am Soc Hematol Educ Program. 2009;87–93.

41. World Malaria Report 2016 (annexes) - WMR-2016-annexes.pdf [Internet]. [cited 2017 May 1]. Available from: http://www.who.int/malaria/publications/world-malaria-report-2016/WMR-2016-annexes.pdf

42. Kovalszki A, Weller PF. Eosinophilia. Prim Care. 2016 Dec;43(4):607–17.

43. O'Connell EM, Nutman TB. Eosinophilia in Infectious Diseases. Immunol Allergy Clin North Am. 2015 Aug;35(3):493–522.

44. Lyke Ke, Dicko A, Dabo A, Sangare L, Kone A, Coulibaly D, Et Al. Association Of Schistosoma Haematobium Infection with Protection Against Acute Plasmodium Falciparum Malaria In Malian Children. Am J Trop Med Hyg. 2005 Dec;73(6):1124–30.

45. Melo GC, Reyes-Lecca RC, Vitor-Silva S, Monteiro WM, Martins M, Benzecry SG, et al. Concurrent Helminthic Infection Protects Schoolchildren with *Plasmodium vivax* from Anemia. PLOS ONE. 2010 Jun 21;5(6):e11206.

46. Giardia: Overview and Update [Internet]. [cited 2017 May 16]. Available from: https://oup.silverchair-cdn.com/oup/backfile/Content_public/Journal/cid/25/3/10.1086/513745/2/25-3-545.pdf?

47. Giardiasis: Background, Pathophysiology, Etiology. 2016 Dec 2 [cited 2017 May 16]; Available from: http://emedicine.medscape.com/article/176718-overview

48. Entamoeba histolytica - Overview [Internet]. Encyclopedia of Life. [cited 2017 May 16]. Available from: http://eol.org/pages/491174/overview

49. Amebiasis: Background, Pathophysiology, Etiology. 2017 Jan 6 [cited 2017 May 16]; Available from: http://emedicine.medscape.com/article/212029-overview

50. Richardson DJ, Callahan KD, Dondji B, Tsekeng P, Richardson KE. Prevalence of Waterborne Protozoan Parasites in Two Rural Villages in the West Province of Cameroon. Comp Parasitol. 2011 Jan;78(1):180–4.

51. Mbuh JV, Ntonifor NH, Ojong J. The epidemiology of soil-transmitted helminth and protozoan infections in south-west Cameroon. J Helminthol. 2012 Mar;86(1):30–7.

52. Mbuh JV, Ntonifor HN, Ojong JT. The incidence, intensity and host morbidity of human parasitic protozoan infections in gastrointestinal disorder outpatients in Buea Sub Division, Cameroon. J Infect Dev Ctries. 2009 Dec 28;4(1):38–43.

53. Kuete T, Yemeli FLS, Mvoa EE, Nkoa T, Somo RM, Ekobo AS. Prevalence and Risk Factors of Intestinal Helminth and Protozoa Infections in an Urban Setting of Cameroon: the Case of Douala. Am J Epidemiol Infect Dis Am J Epidemiol Infect Dis. 2015 Jun 16;3(2):36–44.

54. Diallo TO, Remoue F, Gaayeb L, Schacht A-M, Charrier N, Clerck DD, et al. Schistosomiasis Coinfection in Children Influences Acquired Immune Response against Plasmodium falciparum Malaria Antigens. PLOS ONE. 2010 Sep 15;5(9):e12764.

55. Courtin D, Djilali-Saïah A, Milet J, Soulard V, Gaye O, Migot-Nabias F, et al. Schistosoma haematobium infection affects *Plasmodium falciparum*-specific IgG responses associated with protection against malaria. Parasite Immunol. 2011 Feb 1;33(2):124–31.

56. Hartgers FC, Obeng BB, Kruize YCM, Dijkhuis A, McCall M, Sauerwein RW, et al. Responses to Malarial Antigens Are Altered in Helminth- Infected Children. J Infect Dis. 199:1528–35.

57. Su Z, Segura M, Stevenson MM. Reduced Protective Efficacy of a Blood-Stage Malaria Vaccine by Concurrent Nematode Infection. Infect Immun. 2006 Apr 1;74(4):2138–44.

58. Lemaitre M, Watier L, Briand V, Garcia A, Hesran JYL, Cot M. Coinfection with *Plasmodium falciparum* and S*chistosoma haematobium*: Additional evidence of the protective effect of Schistosomiasis on malaria in Senegalese children. Am J Trop Med Hyg. 2014 Feb 5;90(2):329–34.

59. Entamoeba histolytica: Host Defense and Immune Responses [Internet]. http://www.eurekaselect.com. [cited 2020 Feb 11]. Available from: http://www.eurekaselect.com/54531/chapter

Figure 2

Click here to access/download;Figure;Figure 2.tif

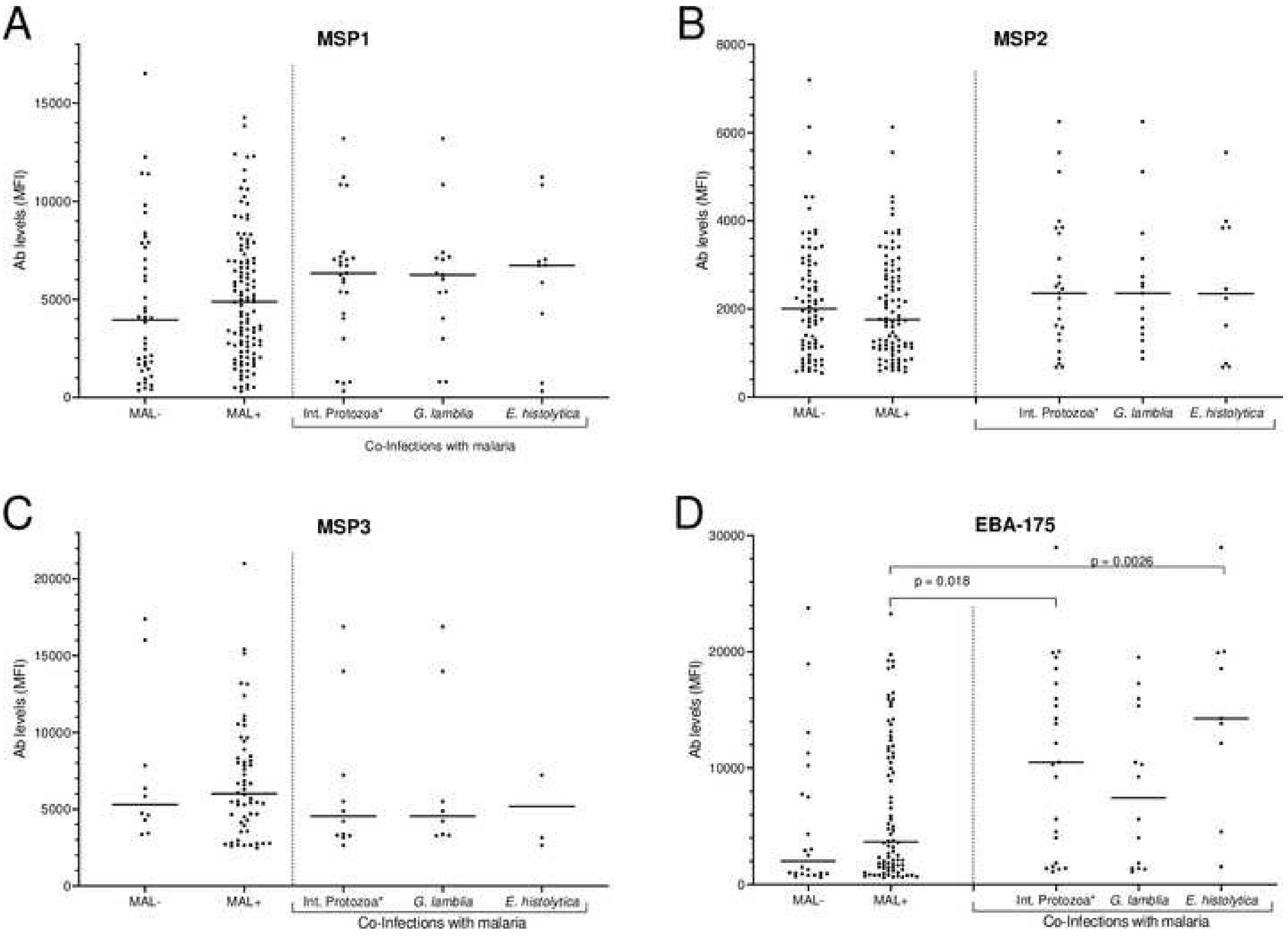

Figure 1

Click here to access/download;Figure;Figure 1.tif

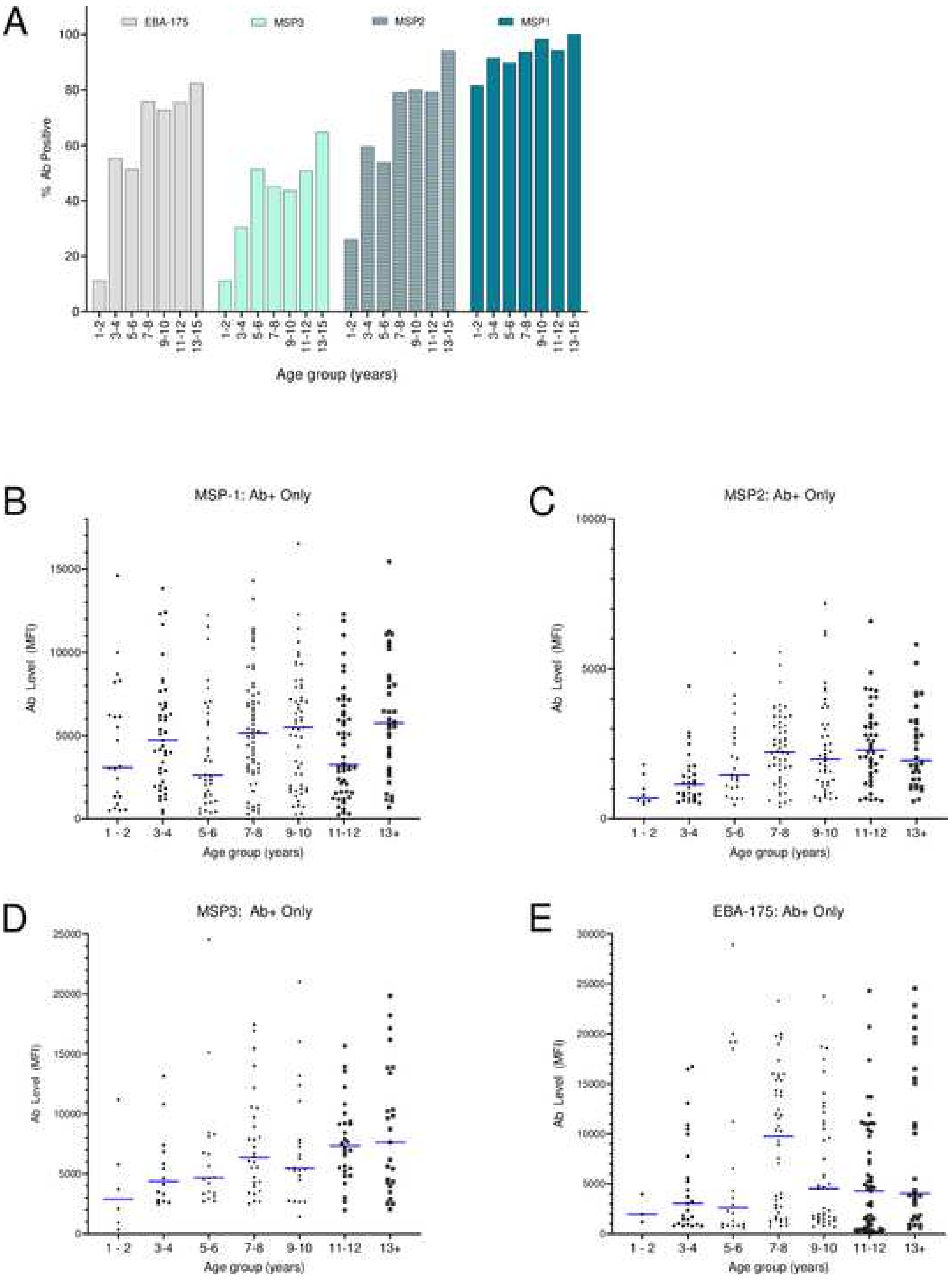

S1 Table

Click here to access/download
**Supporting Information**
S1 Table.docx

Click here to access/download
**Supporting Information**
S Supporting Database.xls

**Full-title: The immunoglobulin G aAntibody Rresponse to Mmalaria Mmerozoite Aantigens in Aasymptomatic cChildren cCo-infected with Mmalaria and Iintestinal Pparasites**

Running title: Malaria antibodies in children with intestinal parasites

7/20/20 – this is the original MS with Reviewer #3 tracks included.

Authors: Ndiabamoh Crespo'o Mbe-cho[1]Crespo'o M. Ndiabamoh[,1,2*], Gabriel L. Ekali[2,5], Livo Esemu[2,3], Yukie M. Lloyd [2,4] Wilfred Mbacham[1,2,3], Jude D. Bigoga[2,3] Diane W. Taylor[4] and Rose G.F. Leke[2*Ψ]

[1]Faculty of Medicine and Biomedical Sciences, University of Yaoundé I, Cameroon

[2]The Biotechnology Center, Nkolbisson, University of Yaoundé I, Cameroon

[3]Department of Biochemistry, University of Yaoundé 1, Cameroon

[4]John A. Burns School of Medicine, University of Hawai'i at Mānoa, USA

[5]National AIDS Control Committee, Ministry of Public Health, Cameroon

*Correspondence to Rose Gana Fomban Leke (roseleke@yahoo.com)(RGFL) and Crespo'o Mbe-cho Ndiabamoh (cndiabamoh@gmail.com) (CMN).

# ABSTRACT

Formatted: Font: 18 pt

## Background

Formatted: Font: 16 pt, Bold

Co-infection with malaria and intestinal parasites is common in children in Africa and may affect their immune response to a malaria parasite infection. Prior studies suggest that co-infections may lead to increased susceptibility to malaria infection and disease severity; however, other studies have shown the reverse. Knowledge on how co-morbidities specifically affect the immune response to malaria antigens is limited. Therefore, this study sought to determine the prevalence of co-infection of malaria and intestinal parasites and its association with antibody levels to malaria merozoite antigens.

## Methods

Formatted: Font: 16 pt, Bold

A cross sectional study was carried out in two villages with high transmission of malaria in Cameroon (Ngali II and Mfou). Children aged 1-15 years were enrolled after obtaining parental consent. A malaria rapid diagnostic test was used on site. Four (4) ml of peripheral blood was collected from each participant for microscopy to determine *Plasmodium falciparum* infections by microscopy and speciation, haemoglobin levels and serology. Fresh stool samples were collected and examined by wet mount, Kato-Katz method and modified Ritchie Rietchi concentration techniques. A Multiplex Analyte Platform (MAP) assay was used to measure antibody levels.

Formatted: Font: Italic

## Results

Formatted: Font: Bold

Formatted: Font: 16 pt, Bold

A total of 320 children were enrolled. The prevalence of malaria by blood smear was 76.3% (244199/230 (75. 6%) and prevalence of malaria and intestinal parasites was co-infections16.9% (-54/320) (16.9%). Malaria prevalence was highest in young children; whereas, intestinal parasites (IP+) were not present until after 3 years of age. All children positive for malaria had antibodies to $MSP1_{42}$, MSP2, MSP3 and EBA175. No difference in antibody levels in children with malaria-co infections compared to malaria alone were found, except for antibody levels to EBA-175 were higher in children co-infected with intestinal

protozoa (amoeba) (p = 0.018), especially those with *Entamoeba histolytica* infections (p=0.0026).

## Conclusion~~:~~

**Formatted:** Font: 16 pt, Bold

**Formatted:** Font: 16 pt

Antibody levels to EBA175 were significantly higher in children co-infected with malaria and *E. histolytica* compared to children infected with malaria alone. It is important to further investigate why and how the presence of these protozoans can modulate the immune response ~~(Th1/Th2)~~ to malaria antigens.

**Key words**: malaria, intestinal-parasites, antibody, *Giardia lamblia, Entamoeba histolytica*

# ~~Background~~Introduction:

In sub-Saharan Africa, malaria caused by *Plasmodium falciparum* (Pf) remains an important public health threat, killing over 271,000 ~~292,000~~ children under the age of five each year(1) ~~(1,2)~~. In malaria endemic areas, individuals exposed to malaria infections gradually develop clinical immunity (2)~~(3)~~ and commonly experience asymptomatic infections without fever or symptoms and do not require antimalarial treatment. Asymptomatic infection results from partial immunity that controls, but does not completely eliminate, malaria parasites, thus allowing for constant presence of circulating parasites (2)~~(3)~~. ~~However, with most children getting infected with several episodes of infections in a short period, this renders them more prone to having clinical symptoms since the immune systems doesn't fully recover.~~

The prevalence of intestinal parasitic infections in children is fairly constant across sub-Saharan Africa with an average prevalence of 26% (3,4)~~(4,5)~~. In Cameroon, the prevalence in children less than 18 years is 26.8% (5)~~(6)~~, while that for the general population is more than 28% The major ~~soil-transmitted~~ intestinal parasites are A*scaris*~~.~~ *lumbricoides, T**richuria*~~.~~ *trichuria and E**ntamoeba*~~.~~ *histolytica* (6–8)~~(7–9)~~, but many cases of intestinal parasites go undetected.

Co-infections with malaria and intestinal parasites (IP) are common in malaria endemic areas in sub-Saharan Africa (7,8)~~(8,9)~~ and infections with IP and Pf are both ranked among the major cause of mortality and morbidity in sub-Saharan Africa. Several studies conducted on IP (not including amoebas) and Pf have shown conflicting results. Some helminths suppress different T-helper types and favor an increase in regulatory T (Treg) cell (9)~~(10)~~. Studies on c~~C~~oncomitant infections in humans ~~have suggested~~ suggest that *A.~~.~~scaris lumbricoides* infection may protect against cerebral malaria (10,11)~~(11,12)~~, while other studies~~,~~ suggest that children infected by *S**chistosoma*~~.~~ *mansoni* *may be* ~~were~~ more susceptible to *P. falciparum* infections and develop acute malaria episodes (12,13)~~(13,14)~~. Also, it has been shown that the levels of TNF-α, IL-2, IL-10, IL-6 in *Plasmodium*-helminth co-infected individuals were significantly higher than the malaria-positive (MP) group (14)~~(15)~~ dampening the immune response to malaria. However, little is known regarding host immune responses to malaria in children co-infected with protozoan pathogens.~~amoebas.~~

**Formatted:** Font: 18 pt

**Formatted:** Font: 18 pt

**Field Code Changed**

**Field Code Changed**

**Field Code Changed**

**Field Code Changed**

**Formatted:** Font: (Default) Arial

**Field Code Changed**

**Field Code Changed**

**Field Code Changed**

**Field Code Changed**

**Field Code Changed**

**Field Code Changed**

Studies suggest that children co-infected with malaria and intestinal helminths had significantly decreased antibody levels to the malarial antigen apical merozoite antigen 1 (AMA-1) compared to those with *P. falciparum* or IP alone(15)(16). Hence, infections with intestinal helminths can stifle protective anti-plasmodial antibody responses (15)(16). However, increase in MSP3 IgG1–4 levels were significantly associated with children infected with malaria alone compared to children co-infected with both parasites(15)(16).

Field Code Changed
Field Code Changed
Field Code Changed

Malaria and other intestinal parasites overlap extensively in their epidemiological distributions causing polyparasitism. Polyparasitism with intestinal parasites has been reported as one of the contributing factors to hypo-responsiveness (16)(17), dampening of the immune response by inducing a strong Treg response, which could in turn, blunt a strong response to vaccines (17)(18). Equally, some studies have suggested an effect of IP on antibody responses to P. falciparum gametocyte antigens that may have consequences on transmission-blocking immunity (18)(19).

Field Code Changed
Field Code Changed
Field Code Changed

Effective elimination and future eradication of malaria will require not only vector control, but also managing asymptomatic malaria patients and developing an effective vaccine. Given the high burden and concomitant nature of both malaria and intestinal parasites in the same geographical setting, conflicting data shows polyparasitism could interfere with the efficacy of malaria vaccines (19)(20). To our knowledge, since limited information is available on whether and how co-infections of intestinal parasites and malaria affect the specific immune response to malaria antigens (20)(21), the goal of this study was to investigate the prevalence and relationship between co-infections of malaria (MAL+) and intestinal parasites (IP+) (nematodes, trematodes, and protozoans) on naturally acquired antibodies to malaria merozoite .

Field Code Changed
Field Code Changed

# Methods:

**Formatted:** Font: 18 pt

## Study Aarea description:

**Formatted:** Font: 16 pt
**Formatted:** Font: 16 pt

The study was conducted in Ngali II and Mfou, two villages in the centeralcentral region of Cameroon (located at 4°27′N and 11°38′E) with a total population of about 1,000 children per squared Km (about 4000 in Ngali II and 6000 in Mfou) under the age of 15 years. The climate

is typically equatorial with two discontinuous dry and rainy seasons. The annual average rainfall measures about 1600 $mm^3$ with an annual average temperature of 23°C (21)~~(22)~~.

Most children in Ngali II and Mfou over 3 years of age accompany their parents to the farm and return home late at night. ~~They seldom sleep under~~ The use of mosquito bed nets is rare in the ~~. The~~ two villages and ~~are geographically similar,~~ residents ~~are relatively poor and~~ have minimal access to portable water~~,~~ with approximately one well per 500 inhabitants. Currently, mass drug administration with albendazole is being performed twice a year by the Ministry of Health, that is usually conduced in schools and symptomatic cases are sent to the local clinic or hospital for follow up treatment.

## Study p~~P~~opulation

A cross sectional study was carried out in Ngali II and Mfou from January to May 2017, a transitional period from the dry to wet season. Children who had lived in either of ~~these two~~ the villages for at least six months and whose parents gave informed consent were included in the study. ~~Since both villages (Ngali II & Mfou) were very similar in all features, data for both village were combined. Vital parameters (temperature, pulse) and anthropometric parameters (weight, height) were measured by assisting attendant nurses. These parameters were used to calculate body mass index (BMI) and advice was given to the parents of the participating children, as part of a service for participation. .~~ All participants were systematically examined by a physician for clinical systems of malaria and IP. ~~Only asymptomatic participants were included in the study.~~ Children who presented with symptoms of malaria, e.g., fever, headaches or intestinal illnesses, e.g., diarrhea, vomiting were not enrolled. A total of 320 participants (140 from Ngali II and 180 from Mfou) aged 1-15 years participated in the study. Since both villages have the same demographic features, data for the two villages were combined.

## Blood ~~C~~collection and ~~O~~on-site ~~T~~testing for ~~M~~malaria

Venous peripheral blood (about 4mL) was collected by venipuncture using a butterfly needle (G22) and a 5mL labeled EDTA tube from all 320 participants. Haemoglobin (Hb) was measured using the HemoCue (AB Leo Diagnostics, Helsingborg, Sweden). On site, after

**Field Code Changed**

**Formatted:** Font: 16 pt

**Formatted:** Font: 16 pt

collecting the venous blood from the participants, a drop from the same collected blood was placed on a CareStart$^{TM}$ Malaria pLDH/HRP-2 Combo Test (Access Bio Inc. USA) to detect histidine-rich protein-2 (HRP-2) specific to *Plasmodium falciparum* and Plasmodium lactate dehydrogenase (pLDH) pan-specific to *Plasmodium* spp (*falciparum, P. vivax, P. malariae, P. ovale*). Results were read according to manufacturer instructions and recorded after 5 minutes.

## Laboratory detection~~, quantification and speciation~~ of malaria parasites~~.~~

Formatted: Font: 16 pt

Ten microliters of whole blood were used to prepare thick and thin smears for malaria parasite identification, speciation and quantification. The slides were air-dried overnight, and the thin blood smears were fixed in absolute (100%) methanol. Both thick and thin smears were stained using 10% Giemsa solution, washed with water and air-dried. Slides were then microscopically examined (thin and thick smear) for the presence of malaria parasites by two experienced microscopists. The parasite density was determined by counting the number of parasites against 200 leucocytes. The counts were expressed as the number of P. falciparum-infected erythrocytes (IE) ~~parasites~~ per micro-liter of blood (Pf IE/µl), assuming an average leukocyte count of 8,000 cells/µl of blood (22)~~(23)~~. When the difference in parasitaemia between the two readers was greater than 5 Pf IE/µl of blood, a third reader re-examined the slide and the mean of the two closest values were considered. Also, a differential count for eosinophil, lymphocytes, monocytes, neutrophils was obtained alongside parasitaemia and different malaria species (by microscopy)

## Antibody A~~a~~nalysis

Formatted: Font: 16 pt

Plasma samples were tested for antibodies against the merozoite antigens MSP-$1_{42}$, MSP-2, MSP-3 and EBA-175 using a multi-analyte platform ~~(MAP)~~ assay with antigen-coupled magnetic beads with different spectral addresses. Details of this assay used has been described previously (23)~~(24)~~ (24)~~(25)~~. In brief, plasma samples were diluted 1:100 with PBS, 50µ~~u~~l of plasma was incubated with 50~~ul~~µl antigen-coupled microspheres (2000 microspheres/test) for 60 minutes in the dark, washed with PBS, and then incubated at

Field Code Changed

Field Code Changed

500rpm for 60minutes at 25 °C on a rotating shaker and using a magnet plate separator. Then, 100 µl of secondary Ab (R-phycoerythrin-conjugated, Affini Pure F(ab′)$_2$ fragment, Goat anti-human IgG Fc fragment specific, Jackson Immuno-research, West Grove, PA, USA, Cat no. 109-116-170) diluted to 2 µg/ml in PBS-1 % BSA was added to each well and incubated as above in the dark for 1 h. The mixture is then washed and a minimum of 100 beads were read in a MAGPIX® reader. A minimum bead count of 100 per spectral address recorded as Median Fluorescence Intensity (MFI).

Controls included on each plate were: PBS to determine background fluorescence, the negative control (NC) consisted of pooled plasma from four malaria-naïve US individuals, and the positive control (PC) was pooled plasma from Cameroonians with high antibody levels to *Plasmodium* falciparum. Results were exported to Excel for analysis. The cut-off for positivity was calculated as mean of MFI +3 standard deviation of the negative control as shown in the results sections.

## Stool sample collection and analysis

Formatted: Font: 16 pt

Sterile labelled stool collection vials were given to the parents along with instructions for proper stool collection. All samples were analyzed within 7 hours of collection to avoid missing hookworm eggs and minimize chances of under reporting. Approximately, 4 mg of feces was suspended in 5ml PBS and a drop examine by wet mount.~~-~~ The Kato Katz technique was used for morphological identification of helminths eggs, e.g., *A.~~scaris.~~ lumbricoides, T.~~richuris~~ trichiura, or* larval stage of *Strongyloides stercoralis* (25)~~(26)~~ while the modified Ritchie's concentration stool technique was used to identify all protozoans and cestodes (26)~~(27)~~. The smears were read at objective 10X for eggs and larvae and objective 40X for cysts and vegetative forms of protozoan. ~~The number of eggs per gram of stool were estimated for the parasites listed. Helminth eggs and protozoans were counted in about 4mg of stool and counts were extrapolated as the number of eggs per gram of stool.~~ All stool slides were read by 2 technicians and in 2 different laboratories under supervision of a microbiologist and parasitologists.

## Data ~~a~~Analysis

Formatted: Font: 16 pt

Formatted: Font: 16 pt

Data were analyzed using Microsoft Excel 2013, and GraphPad® prism 8. Standard summary statistics were used to describe the study population and results are presented as proportions. Fischer's exact test was used to compare antibody levels between~~in~~ the malaria-negative, IP-~~postitive~~positive (MAL-,~~-~~IP~~-~~+~~-~~) and malaria-positive, IP-negative (MAL~~+,IP~~+,IP-) groups, because of the small sample sizes of the groups. The one-way-ANOVA test was used to compare all 4 groups after checking for normality (e.g., age). An unpaired t test was used to compare the means of the MAL-,~~-~~IP- vs. MAL+,~~-~~IP- groups. Kruskal-Wallis test was used to compare~~isons~~ antibody~~ies~~ levels, which are not normally distributed,~~-~~among the groups or within the MAL+IP+ groups. An individual was considered to have a co-infection if at least one IP species and *P. falciparum* were present. Anaemia was considered when Hb values were < 11.5 g/dL and classified according to WHO (27,28)~~(28,29)~~. To search DNA sequences of *P. falciparum* EBA-175 and those of *E. histolytica* for possible cross-reactive epitopes, PfEBA175 (ncbi.nlm.nih.gov/gene/2654998) was compared with E. histolytica (ncbi.nlm.nih.gov/assembly/GCF_000208925.1) using Megablast for highly similar sequences and discontinuous megablast for more dissimilar sequences.

# Results

## The ~~S~~study ~~P~~population

A total of 320 children were enrolled (Table 1). Among the children, 76.3~~5.6~~% were slide-positive for malaria (MAL+), with 59.4~~8.8~~% having malaria without intestinal parasites (MAL+, IP-) and 16.9% being coinfected with malaria and intestinal parasites (MAL+, IP+). All subjects who tested positive for malaria using the rapid diagnostic field test were confirmed positive by microscopy. Among children who were infected with malaria, 71.3% were infected with only *P. falciparum* and 5% had *P. falciparum* and *P. malariae*. Interestingly, only 2.2% of the children had IP without malaria and 21.6~~2.2~~% were negative for both malaria and IP.

The mean age of the children changed with infection status among the 4 groups (p = 0.0001) with the lowest age found in uninfected children (6.4 years) and highest in children with co-infections (9.3 years) (Table 1). Malaria infections were found in all age groups;

**Field Code Changed**

**Formatted:** Font: 18 pt

whereas, none of the children under age 4 years had intestinal parasites. Mean haemoglobin levels were lower in children infected with malaria, but the difference was of marginal significance (p = 0.08; MAL-,IP- vs MAL+,~~,~~IP-). The prevalence of anaemia was higher in children who were infected with malaria (MAL+,IP-)(p=0.032~~4~~), but not those with co-infections (p >0.999) compared to children who were parasite-negative (MAL-,IP-).

| Table 1: Description of 320 children infected with malaria and intestinal parasites (IP) | | | | | |
|---|---|---|---|---|---|
| | MAL-, IP- | MAL+ IP- | MAL-, IP+ | Co-infections (Mal+,~~-~~IP+) | ~~Total~~ P values |
| Number (%) of children | 69 (21.6~~%~~) | 190 (59.4~~%~~) | 7 (2.2~~%~~) | 54 (16.9~~%~~) | ~~320~~ |
| Mean years of age~~*~~ (range) | 6.4 (1-14) | 7.9 (1-15) | 8.6 (4-12) | 9.3 (4-15) | 0.0001* |
| Parasitaemia: (median # infected erythrocytes/µl (range) | 0 | 420~~/µl~~ (40-96,000) | 0 | 900~~/µl~~ (40 –30,970 | 0.1599** |
| Measures of anaemia | | | | | |
| Hb (g/dL) (mean ±SD) | 12.1 ±1.6 | 11.6 ± 2.2~~**~~ | 12.2 ± 1.4 | 12.4±1.8 | 0.0658* |
| Prevalence of anaemia | | | | | |
| # (%) of children with Hb <11.5 g/dL | 21 (30.4~~%~~) | 87 (45.8~~%~~)~~***~~ | 2 (28.6~~%~~) | 17 (31.5) | ~~131~~ 0.0324*** |

*~~p = 0.0001;~~ comparison among the 4 groups (ordinary one-way ANOVA)

** comparison among the 4 groups (Mann-Whitney test)

~~**p = 0.087, comparison between MAL-,IP- vs. MAL+,IP- (unpaired t test).~~

***~~p= 0.032,~~ comparison between MAL-,IP- vs. MAL+,IP- (Fisher's exact test)

## Prevalence of i~~I~~ntestinal p~~P~~arasites

**Formatted:** Font: 16 pt

**Formatted:** Font: 16 pt, Bold, No underline

**Formatted:** Font: 16 pt, Bold, No underline

Overall, 19.1% (61/320) of the children were positive for intestinal parasites, 16.9% of whom were also infected with malaria and 2.2% were IP+ but MAL- (Table 2). The most frequent ~~major of~~ helminthic parasites detected were *A.~~scaris~~ lumbrioides* (2.8%) and single cases of *Trichura sp*. and *Strongyloides sp.* Among the 320 children, 14.7% had detectable protozoan infections, including 7.8% infected with *Giardia lamblia*, 5.9% with *E.~~ntamoeba~~ histolytica*, and 0.9% with *Isospora sp*. Very few children had intestinal cestodes (Table 2). Interestingly, ~~-~~all of the children had single parasite infections, and polyparasitism was not found.

| Table 2: Prevalence of Intestinal Parasites (IP+) in the 320 Children, Ages 1 to 15 years | | | |
|---|---|---|---|
| | Number of Children | | |
| | MAL-, IP+ | MAL+, IP+ | Total IP+ (% positive) |
| Intestinal Parasites | | | |
| **Helminths** | | | **11 (3.4%)** |
| *Ascaris lumbricoides* | 2 | 7 | 9 (2.8%) |
| Others* | 0 | 2 | 2 (0.87%) |
| **Protozoans** | | | **48~~7~~ (14.7%)** |
| *Giradia lamblia* | 3 | 22 | 25 (7.8%) |
| *Entamoeba histolytica* complex | 1 | 18 | 19 (5.9%) |
| Other** | 1 | 3 | 4 (0.9%) |
| **Cestodes** | | | **2 (0.63%)** |
| *Hymenolepis nana* | 0 | 2 | 2 (0.63%) |
| **Total IP** | **7** (2.2%) | **54** (16.9%) | **61** (19.1%) |

Others*: 1 *Trichura sp*. and 1 *Strongyloides sp.*

Others**: 3 *Isospora sp*.

# Influence of ~~A~~age on ~~M~~malaria, ~~I~~intestinal p~~P~~arasites, a~~A~~naemia and m~~M~~oderate e~~E~~osinophilia

As expected, children aged 1 through 2 years did not have soil-transmitted IP and had normal eosinophil levels; whereas, 63% of 1-2-year old~~s~~ children were infected with malaria and had the highest prevalence of anaemia (Table 3). In contrast, in children 9-15 years of age ~80% were slide-positive for malarial parasites, 24%-29% had intestinal parasites, and 10-38% had moderate eosinophilia. Thus, as children living in these villages increased with age, they began developing~~ed~~ partial immunity to malaria symptoms and anaemia declined; whereas, the prevalence of IP and eosinophilia increased.

Table 3: Influence of Age on Malaria, Intestinal Parasites, Anaemia and Percentage of Peripheral Eosinophils

| Age (years) | N = | % Mal+ | % IP+ | * % with anaemia | **% with eosinophilia |
|---|---|---|---|---|---|
| 1 - 2 | 27 | 63.0 | 0 | 55.6 | 0 |
| 3 - 4 | 47 | 61.7 | 6.4 | 48.9 | 4.3 |
| 5-6 | 40 | 62.5 | 15.0 | 40.0 | 7.5 |
| 7-8 | 63 | 88.9 | 28.6 | 36.5 | 9.5 |
| 9-10 | 55 | 83.6 | 21.8 | 38.2 | 20.0 |
| 11-12 | 54 | 79.6 | 25.9 | 38.9 | 22.2 |
| 13-15 | 34 | 82.4 | 23.5 | 26.5 | 38.3 |

*Aneamia: Children with h~~H~~aemoglobin less than 11.5 g/dL. **Moderate eosinophilia: ≥1,500 eosinophils/mm$^3$ or ≥18.7% peripheral eosinophils

A comparison of anaemia and eosinophilia among the 4 groups of children shown in Table 1 was made (S1 Table). Results showed that anaemia was associated with malaria infections and eosinophilia was associated with IP.

# Antibody Levels to Malaria Merozoite Antigens

**Formatted:** Font: 16 pt, Bold, No underline

**Formatted:** Font: 16 pt, Bold, No underline

**Formatted:** Font: 16 pt, Bold, No underline

**Formatted:** Font: 16 pt, Bold, No underline

**Formatted:** Font: 16 pt, Bold, No underline

**Formatted:** Font: 16 pt, Bold, No underline

With repeated exposure to malaria, Ab prevalence and levels increased with age to the four merozoite antigens (Fig~~.~~ 1). Among 1- to 2-year-olds, only 25% of the infants had Ab to EBA-175 and MSP3, 30% had Ab to MSP2, but 80% had Ab to MSP1 (Fig~~ure~~ 1). ~~-~~However, by age 13-15 years, 60% had acquired Ab to MSP3 and >80% had Ab EBA-175, MSP2 and MSP3 (Fig~~.~~ 1A). Among Ab-positive children~~participants~~, Ab levels also increased with age (Fig~~.~~ 1B-E). Although different amounts of Ab were ultimately obtained to the different antigens, the overall trend was for an increase in median Ab with age. ~~-~~Thus, it was important to take age into consideration when making comparison between children infected with malaria (MAL+,IP-), co-infected with malaria and IP (MAL+,IP+) and those who were not infected (MAL-,IP-) at the time the study was conducted.

[Figure 1 – revised]

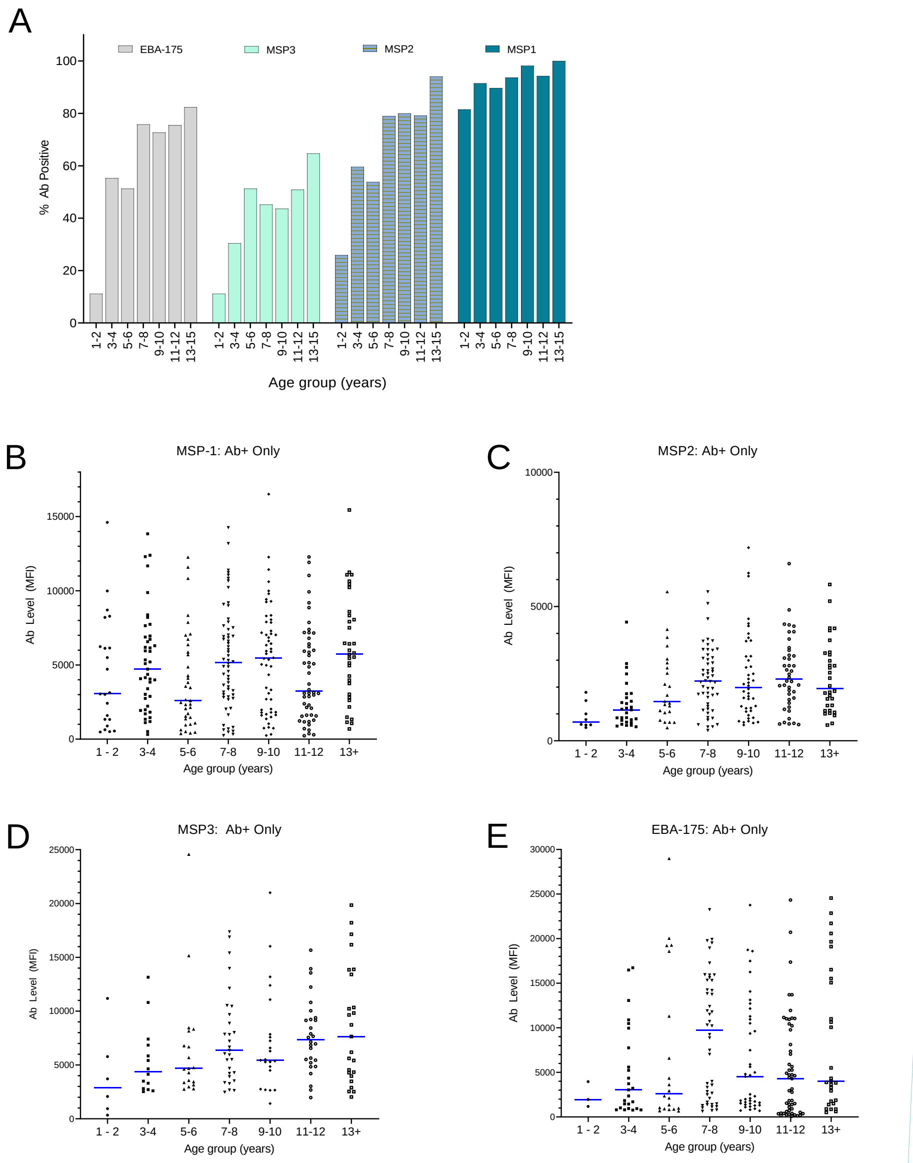


Field Code Changed

**Fig~~ure~~ 1: Prevalence and amount of Ab in different age groups**. (A) Prevalence of Ab to the 4 merozoite antigens. The number of participants in each age group is provided in Table 3. Fig1 B – E show~~s~~ Ab levels (MFI) for children who were Ab-positive for each age group. Horizontal bars represent median Ab levels. Kruskal-Wallis test (nonparametric comparison among groups) values were for MSP1 (p=0.067); MSP2 (p<0.001); MSP3 (p=0.086) and

**Formatted:** Font: Bold, No underline

EBA (p=0.056~~3~~). MFI = Median fluorescence intensity; MSP = merozoite surface proteins; EBA= erythrocytes binding antigen

## Comparison of Ab ~~L~~levels in ~~P~~participants with and without ~~M~~malaria and IP

Formatted: Font: 16 pt, Bold, No underline

Since children below 3 years of age were not infected with IP, they were not included in the comparative studies described below. Given that Ab prevalence and levels increased with age, the study population was divided into 2 groups: children aged 3-10 years, a time period when children were becoming infected with IP (Table 3) and those 11-15 years, mainly children who had been infected repeatedly with malaria and may had lived with IP for a period of time. As predicted, Ab levels were slightly higher in MAL+ children due to current boosting compared to MA~~S~~L-, but the differences were not significant (all p values >0.05) (Fig~~ure~~ 2).

A comparison between Ab levels in children infected with malaria and co-infected with IP was conducted. Children with helminths and cestodes were not included in the analysis because the sample sizes were too small. Ab levels were compared between children aged 3-10 years infected with malaria (n=112) and co-infected with flagellate and intestinal amoeba (n= 25 children), including *G. lamblia* (n= 15) and *E. histolytica* (n = 10 children) (Fig~~.~~ 2). Antibody levels did not differ between malaria-infected children with or without intestinal amoeba for MSP1, MSP2 and MSP3; however, there were significantly higher Ab levels to EBA-175 in children co-infected with malaria and intestinal amoeba (p = 0.018) (Fig~~.~~ 2D). The higher Ab levels were due to *E. histolytica* infections (p=0.0026), and not *G. lamblia* (p=0.3844). No differences were found between children aged 11 to 15 years for any of the antigens between children with malaria (single infection) and co-infected with any of the IP.

To determine if higher Ab levels in children co-infected with *P. falciparum* and *E. histolytica* might be due to cross-reactive epitopes, a BLAST search for sequence homology between EBA-175 and *E. histolytica* proteins. No similarities were found using Metablast, and only one hit was found using discontinuous metablast which had a span of only 38 nucleotides (~13 amino acids) that had 82% similarity. Thus, there does not appear to be

shared epitopes between these two pathogens that would explain the increase in Ab to EBA-175 in children with co-infections.

[FIGURE 2 - revised]

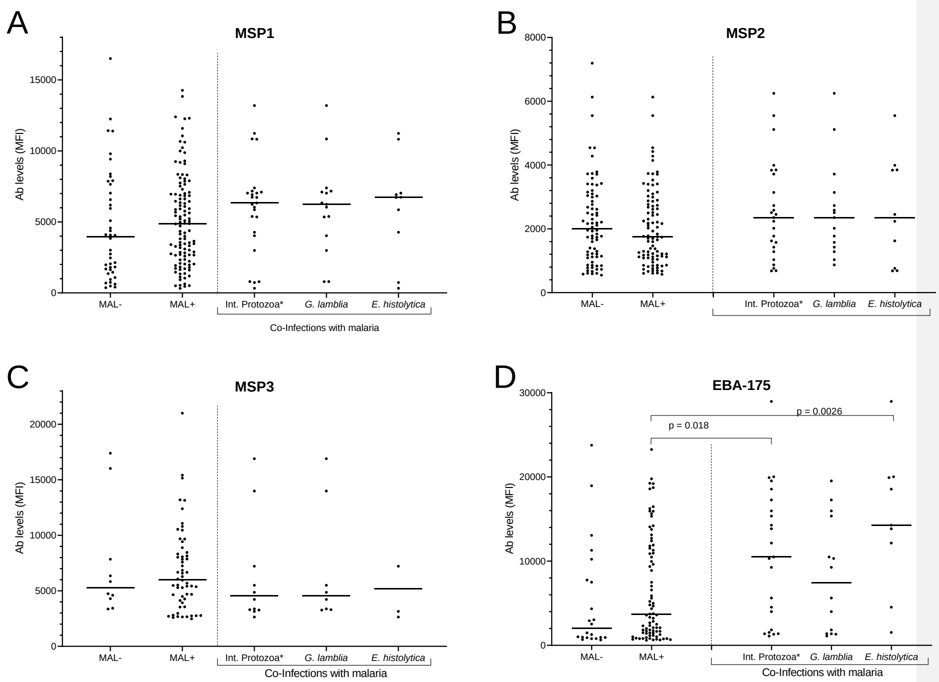


**Fig 2. Antibody levels in children ages 3 to 10 for all antibody-positive individuals**

Distribution of Ab levels in MFI among malaria negative (MAL-) and malaria-positive (MAL+) and those co-infected with malaria plus Intestinal (Int.) ~~amoeba~~protozoa (n=25); malaria plus *G. lamblia* (n=15); and malaria plus *E. histolytica* (n=10). The number of datapoints varied because not all participants had Ab to all antigens. Horizontal lines represent medians for the group. MFI = median florescence intensity; MSP = merozoite surface proteins, EBA = erythrocytes binding antigen (EBA)

# Discussion

**Formatted:** No underline

**Formatted:** Font: 18 pt

Malaria and polyparasitism (cestodes, protozoans, trematodes) are still common conditions throughout Africa (29,30)~~(30,31)~~. In the 1-15-year-old children living in the ~~two~~ rural Cameroonian villages surveyed, the prevalence of slide-positive malaria was 76.3~~5.6~~% and 19.1% had intestinal parasites, with 16.9% co-infections (Table 1-2). This prevalence of malaria is similar to those found in other highly endemic regions of the country (31)~~(32)~~, and the ~~.~~prevalence of co-infections was 19.1%, which is similar to a prevalence of 18 – 27% reported in other regions of Cameroon (32,33)~~(9,44)~~. This high transmission is related to geo-ecological and climatic conditions at the time of the study which was the transition from the dry to wet season, a period that favors vector breeding and distribution (34)~~(33)~~.

From Table 3 ~~above~~, the prevalence of slide-positive malaria ranged from 61% to 90% in different age groups implying that children in these villages were repeatedly exposed to malaria throughout their lives. The current prevalence of malaria in 2017 is similar to that recorded previously for Ngali II between 1998-2004, that ranged from 50% to 85% in 5-15 year olds, with an estimated entomological inoculation rate of 0.7 infectious bites/per/night (~257 infectious bites annually)(35).~~[LEKE ET AL.]~~ Prior studies have established that repeated exposure induces immunity to malaria, with development of anti-disease immunity followed by anti-parasite immunity (36–39)~~(34–37)~~. As a result, the highest prevalence of 56% anaemia was found in young children (2,40,41)~~(3,38,39)~~ with a decline to 27% in 13 to 15-year-olds (Table 3). On the other hand, Infections with IP only occurred later in life from 3 years onward with a mean infection age of 8.1 years. Increase in ~~I~~intestinal parasites was associated with an age-related increase in eosinophil counts (42,43)~~(40,41)~~, a known innate immune response to helminthic and other soil-transmitted organisms (Table 3). In this study, only 11/320 (3.4%) children were infected with helminths. Although some epidemiological studies have demonstrated an increased risk of infection by *P. falciparum* in individuals co-infected with helminths, other results are conflicting (44,45)~~(42,43)~~. The low prevalence of helminths ~~This could be~~ is explained, in part by, the fact that mass community de-worming is done biannually following the national infectious disease guide-line for IP control program. The most prevalent intestinal parasites were the protozoans, *G.~~iardia~~ lamblia ~~intestinalis~~* and *E.~~ntamoeba~~ histolytica* (48). ~~-~~These protozoa are commonly found in damp soil and contaminated water with a prevalence of 2-20% in Cameroon (50–53)~~(48–51)~~. These results

Field Code Changed
Field Code Changed
Field Code Changed
Field Code Changed
Field Code Changed
**Formatted:** Font: (Default) Arial
Field Code Changed
Field Code Changed
Field Code Changed
Field Code Changed
Field Code Changed
**Formatted:** Font: (Default) Arial

suggest children acquire their intestinal infections after learning to walk and interact with the environment. Thus, children in the study population were exposed to malaria early in life and began developing anti-malaria immunity prior to exposure to intestinal parasites.”

Generally, both Ab prevalence and Ab levels increased with age in 1 to 15-year-olds living in this high transmission area (Fig~~.~~ 1). Often, the presence of Ab is used as markers of infection, including the merozoite antigens used in this study. This study compared antibody levels with age in four main groups of children, MAL+,IP+, MAL-,IP-, MAL,-IP-+ and MAL+,IP- children to four (MSP1, MSP2 MSP3, EBA17) malaria antigens (54–56)~~(52–54)~~. Since over 80% of 1-2-year-olds had Ab to MSP1, humoral immunity began to develop early in life and continued to mature as children developed into adolescents (Fig~~.~~ 1). Often individuals who are MAL+ have higher Ab levels than MA~~S~~L- individuals due to boosting of the Ab response (36,38,39)~~(34,36,37)~~. In the current study, Ab levels did not differ significantly between MAL+ and~~than~~ MAL- individuals, neither those who were 3-10 years nor 11-15 years-old. This result~~, however,~~ was not surprising, since 75% of the children were slide-positive for malaria (Table 1). Because of high transmission, ~~Therefore, it is likely that~~ children are becoming infected almost on a daily basis and either are in the process of eliminating the new infection or reducing it to ~~who were slide-negative had either been recently infected or had~~ submicroscopic levels. ~~infections.~~ Thus, most children living in areas with high perennial transmission will test positive for malaria by PCR. ~~In essence, the immune response in individuals who are repeatedly infection would be similar to that produce during chronic infections.~~ *Because of constant re-exposure, the resulting immune response will be similar to that produced by a chronic infection.*

Prior studies have demonstrated that malaria-helminths co-infections can down regulate malaria and orient the immune response via the Th2 response hence, making patients less sick (20,36,57,58)~~(21,34,55,56)~~ whereas, others have demonstrated on the contrary that IP and malaria co-infections increase malaria disease (13,56)~~(14,54)~~. Unfortunately, the current study could not resolve the controversy because very few children had helminthic infections, due to frequent treatment with albendazole via the mass drug administration program

**Formatted:** Font: (Default) Arial
**Field Code Changed**
**Field Code Changed**
**Formatted:** Font: (Default) Arial
**Formatted:** Font: (Default) Arial
**Field Code Changed**
**Field Code Changed**

conducted by the Ministry of Health and other random health campaigns. However, co-infections with malaria and amoeba were relatively common. Ab levels to MSP1, MSP2 and MSP3 were similar in children infected with *P. falciparum* alone and those with amoeba (Fig. 2); however, significantly higher Ab levels to EBA-175 were found in children co-infected with malaria and intestinal amoeba (p = 0.018). The higher Ab levels were due to *E. histolytica* infections (p = 0.0026), and not *G. lamblia* (p = 0.384). This result was unexpected. *E. histolytica* is a gut amoeba that cause both intestinal and extraintestinal infections such as amebic colitis (dysentery) and liver or brain abscess. Thise protozoa can cause a marked down-regulation of macrophage functions rendering the cells incapable of antigen presentation and unresponsive to cytokine stimulation (59)(57). This decrease in macrophage function does not explain the increase in Ab to EBA-175. One possible explanation is that

In addition to a possible immunological interaction, there are at least 2 other explanations as to why *E. histolytica* infections might be associated with higher Ab levels to EBA-175. First, children living in moist or wet environments where mosquitoes breed and *E. histolytica* are more abundant would have a high risk of acquiring both infections, that would result in frequent boosting of the Ab response. Secondly, since malaria and *E. histolytica* are both protozoan pathogensamoebae, they might share common antigens, for example, EBA-175 could share homology with an *E. histolytica* antigen. To investigate this possibility, a blast search of the NCIB gene bank was conducted for EBA-175 and the *E. histolytica* genome. However, this search revealed only a ~13 amino acid sequence with 82% similarity, which is clear too small to explain the increase in Ab levels of co-infected children. showed no significant similarity between both gene sequences. Finally, an alternative explanation could be that this result is a spurious observation by chance. Clearly the association between malaria and *E. histolytica* merits further study.

Altogether, a keen observation needs to be repeated with a larger sample size as *E. histolytica* boosting of Ab to EBA-175 – co-infection might not only be limited to EBA-175, but other antigens as well. Children in these villages began to acquire an Ab response to the 4 merozoite antigens early in life, prior to infection with IP. -There was no evidence that

**Field Code Changed**

**Formatted:** Highlight

infection with IP influenced Ab levels or negatively-altered the already established Ab response to the 4 merozoite antigens.

# CONCLUSION

The prevalence of malaria was high in children 1-2 years old; whereas, intestinal parasite infections occurred in children over 3 years old. Thus, immunity to *P. falciparum* began prior to infection with soil-transmitted parasites. No differences were found in antibody prevalence or levels in malaria-infected and co-infected children, except antibody levels to EBA175 were significantly higher in children co-infected with malaria and *E. histolytica*.   This is the first report of an interaction between malaria and *E. histolytica* and antibodies to EBA-175 and merits further evaluation.

# Declarations

## Ethical consideration

Ethical clearance used for the study was obtained from the Cameroon National Ethics Committee (IRB approval: Nº2016/12/845/CE/CNERSH/SP). Administrative authorizations were obtained from authorities of the Ngali II and Mfou health districts.

Informed consents were obtained from parents of all participants. A clinical examination was performed for all eligible participants by a medical doctor.

All participants positive for any *Plasmodium* spp by RDT at the time of blood collection and those who were found to have PI by stool analysis were treated for free following the protocol recommended by the Cameroonian Ministry of Health. All children with mild anaemia were given an iron supplement free of charge.

## Authors' contributions

Formatted: Font: 18 pt

Formatted: Font: 18 pt

Formatted: Font: 16 pt

Formatted: Font: 16 pt

GFLR supervised the study. JDB and WM co-supervised the study. ~~N~~CMN and ELG conceived and designed the work. ~~N~~CMN, EFL, DJC, AEW, MBN carried out experiments. Data was collected and analyzed by ~~N~~CMN. The first draft of this manuscript was written by ~~N~~CMN, critically read and edited by DWT, YML. DWT and GFLR reviewed the final draft.


## Acknowledgements

We heartily thank all the participants and their parents who let them participate in the study in Ngali II and Mfou. We equally express our gratitude to the community health workers of these areas for their assistance during sample collection.

Anna Babakhanyan, of Hawaii university provided the recombinant proteins (beads) used for the assays and the entire staff of the Biotechnology Centre of University of Yaoundé I, Cameroon for their tremendous work in the field during sample collection and sample processing in the lab.

## Funding s~~S~~tatement~~:~~

~~The beats used for the experiments were a gift from A. Babakhanyan (University of Hawaii) and coupled by ELG. The MAGPIX used for the data analysis was MAGPX I3038703, Luminex corporation 12212 technology Blvd Austin.~~ The Luminex MAGpix was provided by grant P30GM11473, Centers of Biomedical Research Excellence, National Institute of General Medical Sciences, NIH. ~~Texas 78727.~~ GFLR provided the funds for the field work and analysis.


## Competing i~~I~~nterest~~:~~

Authors declare no competing interests.

## Data Availability:

The database for the study can be found in the "Supporting Material File." ~~The authors approve of the availability of all data underlying the findings and without restriction upon reasonable request from the corresponding authors. All important data are within the paper.~~

**Formatted:** Font: 16 pt

**Formatted:** Font: 16 pt

**Formatted:** Font: 16 pt

**Formatted:** Font: 16 pt

**Formatted:** Font: 16 pt

**Formatted:** Not Highlight

## Consent for publication:

All authors give their consent for publication of this manuscript.

## References:


1. World-Malaria-Report-2019-briefing-kit-eng.pdf [Internet]. [cited 2020 Jul 6]. Available from: https://www.who.int/malaria/publications/world-malaria-report-2019/World-Malaria-Report-2019-briefing-kit-eng.pdf

2. Chen I, Clarke SE, Gosling R, Hamainza B, Killeen G, Magill A, et al. "Asymptomatic" Malaria: A Chronic and Debilitating Infection That Should Be Treated. PLOS Med. 2016 Jan 19;13(1):e1001942.

3. Pampiglione S, Visconti S, Pezzino G. [Human intestinal parasites in Subsaharan Africa. II. Sao Tomé and Principe]. Parassitologia. 1987 Apr;29(1):15–25.

4. Fletcher SM, Stark D, Ellis J. Prevalence of gastrointestinal pathogens in Sub-Saharan Africa: systematic review and meta-analysis. J Public Health Afr [Internet]. 2011 Sep 5 [cited 2018 Sep 9];2(2). Available from: https://www.ncbi.nlm.nih.gov/pmc/articles/PMC5345503/

5. Fusi-Ngwa C, Besong E, Pone JW, Mbida M. A Cross-Sectional Study of Intestinal Parasitic Infections in Children in Ghettoed, Diverse and Affluent Communities in Dschang, West Region, Cameroon. Open Access Libr J. 2014 Dec 1;01:1.

6. Mwangi TW, Bethony JM, Brooker S. Malaria and helminth interactions in humans: an epidemiological viewpoint. Ann Trop Med Parasitol. 2006 Oct;100(7):551–70.

7. Zeukeng F, Tchinda VHM, Bigoga JD, Seumen CHT, Ndzi ES, Abonweh G, et al. Co-infections of Malaria and Geohelminthiasis in Two Rural Communities of Nkassomo and Vian in the Mfou Health District, Cameroon. PLoS Negl Trop Dis [Internet]. 2014 Oct 16 [cited 2016 Dec 5];8(10). Available from: http://www.ncbi.nlm.nih.gov/pmc/articles/PMC4199518/

8. Njunda AL, Fon SG, Assob JCN, Nsagha DS, Kwenti TDB, Kwenti TE. Coinfection with malaria and intestinal parasites, and its association with anaemia in children in Cameroon. Infect Dis Poverty [Internet]. 2015 Oct 6 [cited 2016 Dec 8];4. Available from: http://www.ncbi.nlm.nih.gov/pmc/articles/PMC4595138/

9. Hopkin J. Immune and genetic aspects of asthma, allergy and parasitic worm infections: evolutionary links. Parasite Immunol. 2009;31(5):267–73.

10. Nacher M, Gay F, Singhasivanon P, Krudsood S, Treeprasertsuk S, Mazier D, et al. Ascaris lumbricoides infection is associated with protection from cerebral malaria. Parasite Immunol. 2000 Mar 1;22(3):107–13.

11. Brutus L, Watier L, Hanitrasoamampionona V, Razanatsoarilala H, Cot M. Confirmation of the protective effect of Ascaris lumbricoides on Plasmodium falciparum infection: results of a randomized trial in Madagascar. Am J Trop Med Hyg. 2007 Dec;77(6):1091–5.


**Formatted:** Font: 16 pt

**Formatted:** Font: 18 pt

**Formatted:** Bibliography, Widow/Orphan control, Adjust space between Latin and Asian text, Adjust space between Asian text and numbers

12. Author(S): M. Nacher, P. Singhasivanon, S. Yimsamran, W. Manibunyong, N. Thanyavanich, P., Wuthisen, And S. Looareesuwan, Author(S): M. Nacher, P. Singhasivanon, S. Yimsamran, W. Manibunyong, N. Thanyavanich, P. Intestinal Helminth Infections Are Associated With Increased Incidence Of Plasmodium Falciparum Malaria In Thailand [Internet]. [Cited 2017 May 1]. Available From: Http://Sci-Hub.Cc/Http://Dx.Doi.Org/10.1645/0022-3395(2002)088[0055:Ihiaaw]2.0.Co;2

13. Sokhna C, Le Hesran J-Y, Mbaye PA, Akiana J, Camara P, Diop M, et al. Increase of malaria attacks among children presenting concomitant infection by Schistosoma mansoni in Senegal. Malar J. 2004;3:43.

14. Lo AC, Faye B, Gyan BA, Amoah LE. Plasmodium and intestinal parasite perturbations of the infected host's inflammatory responses: a systematic review. Parasit Vectors [Internet]. 2018 Jul 3 [cited 2019 Sep 16];11. Available from: https://www.ncbi.nlm.nih.gov/pmc/articles/PMC6031113/

15. Njua-Yafi C, Nkuo-Akenji T, Anchang-Kimbi J, Apinjoh T, Mugri R, Chi H, et al. The Effect of Helminth Co-Infection on Malaria-Specific Immunoglobulin G Responses. BMJ Glob Health. 2017 Feb 1;2(Suppl 2):A66–A66.

16. Nacher M. Malaria vaccine trials in a wormy world. Trends Parasitol. 2001 Dec 1;17(12):563–5.

17. Maizels RM, Pearce EJ, Artis D, Yazdanbakhsh M, Wynn TA. Regulation of pathogenesis and immunity in helminth infections. J Exp Med. 2009 Sep 28;206(10):2059–66.

18. Ateba-Ngoa U, Jones S, Zinsou JF, Honkpehedji J, Adegnika AA, Agobe J-CD, et al. Associations between Helminth Infections, Plasmodium falciparum Parasite Carriage and Antibody Responses to Sexual and Asexual Stage Malarial Antigens. Am J Trop Med Hyg. 2016 Aug 3;95(2):394–400.

19. Hartgers FC, Yazdanbakhsh M. Co-infection of helminths and malaria: modulation of the immune responses to malaria. Parasite Immunol. 2006 Oct 1;28(10):497–506.

20. Sanchez, nchez-Arcila JC, Perce-da-Silva D de S, Vasconcelos MPA, Rodrigues-da-Silva RN, Pereira VA, et al. Intestinal Parasites Coinfection Does Not Alter Plasma Cytokines Profile Elicited in Acute Malaria in Subjects from Endemic Area of Brazil. Mediators Inflamm. 2014 Sep 16;2014:e857245.

21. Kuitcha D, Fouepe A, Ndjama J, Takem G, Awah M, Kamgang V. Chemical and isotopic signal of precipitation in Yaounde-Cameroon. Arch Appl Sci Res. 2012 Oct 15;4:2591–7.

22. District Laboratory Practice in Tropical Countries Part 1, Second Edition - monica-cheesbrough-district-laboratory-practice-in-tropical-countries-part-1.pdf [Internet]. [cited 2017 Nov 14]. Available from: https://medicallabtechno.weebly.com/uploads/7/5/1/5/7515789/monica-cheesbrough-district-laboratory-practice-in-tropical-countries-part-1.pdf

23. Fodjo BAY, Atemnkeng N, Esemu L, Yuosembom EK, Quakyi IA, Tchinda VHM, et al. Antibody responses to the full-length VAR2CSA and its DBL domains in Cameroonian children and teenagers. Malar J [Internet]. 2016 [cited 2020 Feb 8];15. Available from: https://www.ncbi.nlm.nih.gov/pmc/articles/PMC5097422/

24. Babakhanyan A, Fang R, Wey A, Salanti A, Sama G, Efundem C, et al. Comparison of the specificity of antibodies to VAR2CSA in Cameroonian multigravidae with and without placental malaria: a retrospective case–control study. Malar J. 2015 Dec 1;14(1):480.

25. Lindholz CG, Favero V, Verissimo C de M, Candido RRF, Souza RP de, Santos RR dos, et al. Study of diagnostic accuracy of Helmintex, Kato-Katz, and POC-CCA methods for diagnosing intestinal schistosomiasis in Candeal, a low intensity transmission area in northeastern Brazil. PLoS Negl Trop Dis. 2018 Mar 8;12(3):e0006274.

26. Yanet F-S, Fidel Angel N-F, Guillermo N, Sergio S-P. Comparison of parasitological techniques for the diagnosis of intestinal parasitic infections in patients with presumptive malabsorption. J Parasit Dis Off Organ Indian Soc Parasitol. 2017 Sep;41(3):718–22.

27. haemoglobin_en.doc - haemoglobin WHO .pdf [Internet]. [cited 2017 Aug 21]. Available from: http://www.who.int/vmnis/indicators/haemoglobin.pdf

28. Hemoglobin Concentration (Hb): Reference Range, Interpretation, Collection and Panels. 2016 Jun 1 [cited 2017 May 18]; Available from: http://emedicine.medscape.com/article/2085614-overview

29. Degarege A, Veledar E, Degarege D, Erko B, Nacher M, Madhivanan P. Plasmodium falciparum and soil-transmitted helminth co-infections among children in sub-Saharan Africa: a systematic review and meta-analysis. Parasit Vectors [Internet]. 2016 Jun 15 [cited 2020 Feb 11];9. Available from: https://www.ncbi.nlm.nih.gov/pmc/articles/PMC4908807/

30. Hürlimann E, Yapi RB, Houngbedji CA, Schmidlin T, Kouadio BA, Silué KD, et al. The epidemiology of polyparasitism and implications for morbidity in two rural communities of Côte d'Ivoire. Parasit Vectors. 2014 Feb 25;7:81.

31. Akinbo FO, Omoregie R, Mordi R, Okaka CE. Prevalence of Malaria and Anemia Among Young Children in a Tertiary Hospital in Benin City, Edo State, Nigeria. Fooyin J Health Sci. 2009 Nov 1;1(2):81–4.

32. Nkuo-Akenji TK, Chi PC, Cho JF, Ndamukong KKJ, Sumbele I. Malaria and helminth co-infection in children living in a malaria endemic setting of mount Cameroon and predictors of anemia. J Parasitol. 2006 Dec;92(6):1191–5.

33. Malaria, helminths, co-infection and anaemia in a cohort of children from Mutengene, south western Cameroon | Malaria Journal | Full Text [Internet]. [cited 2020 Jul 13]. Available from: https://malariajournal.biomedcentral.com/articles/10.1186/s12936-016-1111-2

34. Achonduh-Atijegbe OA, Mfuh KO, Mbange AHE, Chedjou JP, Taylor DW, Nerurkar VR, et al. Prevalence of malaria, typhoid, toxoplasmosis and rubella among febrile children in Cameroon. BMC Infect Dis. 2016 Nov 8;16(1):658.

35. Rf L, Jd B, J Z, Gg F, Rj L, V T, et al. Longitudinal studies of Plasmodium falciparum malaria in pregnant women living in a rural Cameroonian village with high perennial transmission. Am J Trop Med Hyg. 2010 Nov 1;83(5):996–1004.

36. Artavanis-Tsakonas K, Tongren Je, Riley Em. The War Between The Malaria Parasite And The Immune System: Immunity, Immunoregulation And Immunopathology. Clin Exp Immunol. 2003 Aug;133(2):145–52.

37. Roetynck S, Baratin M, Vivier É, Ugolini S. Cellules natural killer et immunité innée contre le paludisme. médecine/sciences. 2006 Aug 1;22(8–9):739–44.

38. Griffin JT, Hollingsworth TD, Reyburn H, Drakeley CJ, Riley EM, Ghani AC. Gradual acquisition of immunity to severe malaria with increasing exposure. Proc R Soc B Biol Sci [Internet]. 2015 Feb 22 [cited 2020 Feb 11];282(1801). Available from: https://www.ncbi.nlm.nih.gov/pmc/articles/PMC4309004/

39. Bediako Y, Adams R, Reid AJ, Valletta JJ, Ndungu FM, Sodenkamp J, et al. Repeated clinical malaria episodes are associated with modification of the immune system in children. BMC Med [Internet]. 2019 Mar 13 [cited 2020 Feb 11];17. Available from: https://www.ncbi.nlm.nih.gov/pmc/articles/PMC6415347/

40. Haldar K, Mohandas N. Malaria, erythrocytic infection, and anemia. Hematol Am Soc Hematol Educ Program. 2009;87–93.

41. World Malaria Report 2016 (annexes) - WMR-2016-annexes.pdf [Internet]. [cited 2017 May 1]. Available from: http://www.who.int/malaria/publications/world-malaria-report-2016/WMR-2016-annexes.pdf

42. Kovalszki A, Weller PF. Eosinophilia. Prim Care. 2016 Dec;43(4):607–17.

43. O'Connell EM, Nutman TB. Eosinophilia in Infectious Diseases. Immunol Allergy Clin North Am. 2015 Aug;35(3):493–522.

44. Lyke Ke, Dicko A, Dabo A, Sangare L, Kone A, Coulibaly D, Et Al. Association Of Schistosoma Haematobium Infection with Protection Against Acute Plasmodium Falciparum Malaria In Malian Children. Am J Trop Med Hyg. 2005 Dec;73(6):1124–30.

45. Melo GC, Reyes-Lecca RC, Vitor-Silva S, Monteiro WM, Martins M, Benzecry SG, et al. Concurrent Helminthic Infection Protects Schoolchildren with Plasmodium vivax from Anemia. PLOS ONE. 2010 Jun 21;5(6):e11206.

46. Giardia: Overview and Update [Internet]. [cited 2017 May 16]. Available from: https://oup.silverchair-cdn.com/oup/backfile/Content_public/Journal/cid/25/3/10.1086/513745/2/25-3-545.pdf?

47. Giardiasis: Background, Pathophysiology, Etiology. 2016 Dec 2 [cited 2017 May 16]; Available from: http://emedicine.medscape.com/article/176718-overview

48. Entamoeba histolytica - Overview [Internet]. Encyclopedia of Life. [cited 2017 May 16]. Available from: http://eol.org/pages/491174/overview

49. Amebiasis: Background, Pathophysiology, Etiology. 2017 Jan 6 [cited 2017 May 16]; Available from: http://emedicine.medscape.com/article/212029-overview

50. Richardson DJ, Callahan KD, Dondji B, Tsekeng P, Richardson KE. Prevalence of Waterborne Protozoan Parasites in Two Rural Villages in the West Province of Cameroon. Comp Parasitol. 2011 Jan;78(1):180–4.

51. Mbuh JV, Ntonifor NH, Ojong J. The epidemiology of soil-transmitted helminth and protozoan infections in south-west Cameroon. J Helminthol. 2012 Mar;86(1):30–7.

52. Mbuh JV, Ntonifor HN, Ojong JT. The incidence, intensity and host morbidity of human parasitic protozoan infections in gastrointestinal disorder outpatients in Buea Sub Division, Cameroon. J Infect Dev Ctries. 2009 Dec 28;4(1):38–43.

53. Kuete T, Yemeli FLS, Mvoa EE, Nkoa T, Somo RM, Ekobo AS. Prevalence and Risk Factors of Intestinal Helminth and Protozoa Infections in an Urban Setting of Cameroon: the Case of Douala. Am J Epidemiol Infect Dis Am J Epidemiol Infect Dis. 2015 Jun 16;3(2):36–44.

54. Diallo TO, Remoue F, Gaayeb L, Schacht A-M, Charrier N, Clerck DD, et al. Schistosomiasis Coinfection in Children Influences Acquired Immune Response against Plasmodium falciparum Malaria Antigens. PLOS ONE. 2010 Sep 15;5(9):e12764.

55. Courtin D, Djilali-Saïah A, Milet J, Soulard V, Gaye O, Migot-Nabias F, et al. Schistosoma haematobium infection affects Plasmodium falciparum-specific IgG responses associated with protection against malaria. Parasite Immunol. 2011 Feb 1;33(2):124–31.

56. Hartgers FC, Obeng BB, Kruize YCM, Dijkhuis A, McCall M, Sauerwein RW, et al. Responses to Malarial Antigens Are Altered in Helminth- Infected Children. J Infect Dis. 199:1528–35.

57. Su Z, Segura M, Stevenson MM. Reduced Protective Efficacy of a Blood-Stage Malaria Vaccine by Concurrent Nematode Infection. Infect Immun. 2006 Apr 1;74(4):2138–44.

58. Lemaitre M, Watier L, Briand V, Garcia A, Hesran JYL, Cot M. Coinfection with Plasmodium falciparum and Schistosoma haematobium: Additional Evidence of the Protective Effect of Schistosomiasis on Malaria in Senegalese Children. Am J Trop Med Hyg. 2014 Feb 5;90(2):329–34.

59. Entamoeba histolytica: Host Defense and Immune Responses [Internet]. http://www.eurekaselect.com. [cited 2020 Feb 11]. Available from: http://www.eurekaselect.com/54531/chapter

1. World Malaria Report 2016 - 9789241511711-eng.pdf [Internet]. [cited 2016 Dec 17]. Available from: http://apps.who.int/iris/bitstream/10665/252038/1/9789241511711-eng.pdf

2. WHO | World Malaria Day 2016: End malaria for good [Internet]. WHO. [cited 2016 Nov 26]. Available from: http://www.who.int/campaigns/malaria-day/2016/event/en/

3. Chen I, Clarke SE, Gosling R, Hamainza B, Killeen G, Magill A, et al. “Asymptomatic” Malaria: A Chronic and Debilitating Infection That Should Be Treated. PLOS Med. 2016 Jan 19;13(1):e1001942.

4. Pampiglione S, Visconti S, Pezzino G. [Human intestinal parasites in Subsaharan Africa. II. Sao Tomé and Principe]. Parassitologia. 1987 Apr;29(1):15–25.

5. Fletcher SM, Stark D, Ellis J. Prevalence of gastrointestinal pathogens in Sub-Saharan Africa: systematic review and meta-analysis. J Public Health Afr [Internet]. 2011 Sep 5 [cited 2018 Sep 9];2(2). Available from: https://www.ncbi.nlm.nih.gov/pmc/articles/PMC5345503/

6. Fusi-Ngwa C, Besong E, Pone JW, Mbida M. A Cross-Sectional Study of Intestinal Parasitic Infections in Children in Ghettoed, Diverse and Affluent Communities in Dschang, West Region, Cameroon. Open Access Libr J. 2014 Dec 1;01:1.

7. Mwangi TW, Bethony JM, Brooker S. Malaria and helminth interactions in humans: an epidemiological viewpoint. Ann Trop Med Parasitol. 2006 Oct;100(7):551–70.


**Formatted:** Font: (Default) Times New Roman

**Formatted:** Font: (Default) Times New Roman, 12 pt

**Formatted:** Font: (Default) Times New Roman

**Formatted:** Font: (Default) Times New Roman, 12 pt

**Formatted:** Font: (Default) Times New Roman

**Formatted:** Font: (Default) Times New Roman, 12 pt

**Formatted:** Font: (Default) Times New Roman

**Formatted:** Font: (Default) Times New Roman, 12 pt

**Formatted:** Font: (Default) Times New Roman

**Formatted:** Font: (Default) Times New Roman, 12 pt

**Formatted:** Font: (Default) Times New Roman

**Formatted:** Font: (Default) Times New Roman, 12 pt

**Formatted:** Font: (Default) Times New Roman

**Formatted:** Font: (Default) Times New Roman, 12 pt

8. Zeukeng F, Tchinda VHM, Bigoga JD, Seumen CHT, Ndzi ES, Abonweh G, et al. Co-infections of Malaria and Geohelminthiasis in Two Rural Communities of Nkassomo and Vian in the Mfou Health District, Cameroon. PLoS Negl Trop Dis [Internet]. 2014 Oct 16 [cited 2016 Dec 5];8(10). Available from: http://www.ncbi.nlm.nih.gov/pmc/articles/PMC4199518/

9. Njunda AL, Fon SG, Assob JCN, Nsagha DS, Kwenti TDB, Kwenti TE. Coinfection with malaria and intestinal parasites, and its association with anaemia in children in Cameroon. Infect Dis Poverty [Internet]. 2015 Oct 6 [cited 2016 Dec 8];4. Available from: http://www.ncbi.nlm.nih.gov/pmc/articles/PMC4595138/

10. Hopkin J. Immune and genetic aspects of asthma, allergy and parasitic worm infections: evolutionary links. Parasite Immunol. 2009;31(5):267–73.

11. Nacher M, Gay F, Singhasivanon P, Krudsood S, Treeprasertsuk S, Mazier D, et al. Ascaris lumbricoides infection is associated with protection from cerebral malaria. Parasite Immunol. 2000 Mar 1;22(3):107–13.

12. Brutus L, Watier L, Hanitrasoamampionona V, Razanatsoarilala H, Cot M. Confirmation of the protective effect of Ascaris lumbricoides on *Plasmodium falciparum* infection: results of a randomized trial in Madagascar. Am J Trop Med Hyg. 2007 Dec;77(6):1091–5.

13. Author(s): M. Nacher, P. Singhasivanon, S. Yimsamran, W. Manibunyong, N. Thanyavanich, P., Wuthisen, and S. Looareesuwan, Author(s): M. Nacher, P. Singhasivanon, S. Yimsamran, W. Manibunyong, N. Thanyavanich, P. INTESTINAL HELMINTH INFECTIONS ARE ASSOCIATED WITH INCREASED INCIDENCE OF PLASMODIUM FALCIPARUM MALARIA IN THAILAND [Internet]. [cited 2017 May 1]. Available from: http://sci-hub.cc/http://dx.doi.org/10.1645/0022-3395(2002)088[0055:IHIAAW]2.0.CO;2

14. Sokhna C, Le Hesran J-Y, Mbaye PA, Akiana J, Camara P, Diop M, et al. Increase of malaria attacks among children presenting concomitant infection by Schistosoma mansoni in Senegal. Malar J. 2004;3:43.

15. Lo AC, Faye B, Gyan BA, Amoah LE. Plasmodium and intestinal parasite perturbations of the infected host's inflammatory responses: a systematic review. Parasit Vectors [Internet]. 2018 Jul 3 [cited 2019 Sep 16];11. Available from: https://www.ncbi.nlm.nih.gov/pmc/articles/PMC6031113/

16. Njua-Yafi C, Nkuo-Akenji T, Anchang-Kimbi J, Apinjoh T, Mugri R, Chi H, et al. The Effect of Helminth Co-Infection on Malaria-Specific Immunoglobulin G Responses. BMJ Glob Health. 2017 Feb 1;2(Suppl 2):A66–A66.

17. Nacher M. Malaria vaccine trials in a wormy world. Trends Parasitol. 2001 Dec 1;17(12):563–5.

18. Maizels RM, Pearce EJ, Artis D, Yazdanbakhsh M, Wynn TA. Regulation of pathogenesis and immunity in helminth infections. J Exp Med. 2009 Sep 28;206(10):2059–66.

19. Ateba-Ngoa U, Jones S, Zinsou JF, Honkpehedji J, Adegnika AA, Agobe J-CD, et al. Associations between Helminth Infections, Plasmodium falciparum Parasite Carriage and Antibody Responses to Sexual and Asexual Stage Malarial Antigens. Am J Trop Med Hyg. 2016 Aug 3;95(2):394–400.


**Formatted:** Font: (Default) Times New Roman

**Formatted:** Font: (Default) Times New Roman, 12 pt

**Formatted:** Font: (Default) Times New Roman

**Formatted:** Font: (Default) Times New Roman, 12 pt

**Formatted:** Font: (Default) Times New Roman

**Formatted:** Font: (Default) Times New Roman, 12 pt

**Formatted:** Font: (Default) Times New Roman

**Formatted:** Font: (Default) Times New Roman, 12 pt

**Formatted:** Font: (Default) Times New Roman

**Formatted:** Font: (Default) Times New Roman, Italic

**Formatted:** Font: (Default) Times New Roman

**Formatted:** Font: (Default) Times New Roman, 12 pt

**Formatted:** Font: (Default) Times New Roman

**Formatted:** Font: (Default) Times New Roman, 12 pt

**Formatted:** Font: (Default) Times New Roman

**Formatted:** Font: (Default) Times New Roman, 12 pt

**Formatted:** Font: (Default) Times New Roman

**Formatted:** Font: (Default) Times New Roman, 12 pt

**Formatted:** Font: (Default) Times New Roman

**Formatted:** Font: (Default) Times New Roman, 12 pt

**Formatted:** Font: (Default) Times New Roman

**Formatted:** Font: (Default) Times New Roman, 12 pt

**Formatted:** Font: (Default) Times New Roman

**Formatted:** Font: (Default) Times New Roman, 12 pt

**Formatted:** Font: (Default) Times New Roman

**Formatted:** Font: (Default) Times New Roman, 12 pt

20. Hartgers FC, Yazdanbakhsh M. Co-infection of helminths and malaria: modulation of the immune responses to malaria. Parasite Immunol. 2006 Oct 1;28(10):497–506.

21. Sanchez-Arcila JC, Perce-da-Silva D de S, Vasconcelos MPA, Rodrigues-da-Silva RN, Pereira VA, et al. Intestinal Parasites Coinfection Does Not Alter Plasma Cytokines Profile Elicited in Acute Malaria in Subjects from Endemic Area of Brazil. Mediators Inflamm. 2014 Sep 16;2014:e857245.

22. Kuitcha D, Fouepe A, Ndjama J, Takem G, Awah M, Kamgang V. Chemical and isotopic signal of precipitation in Yaounde-Cameroon. Arch Appl Sci Res. 2012 Oct 15;4:2591–7.

23. District Laboratory Practice in Tropical Countries Part 1, Second Edition - monica-cheesbrough-district-laboratory-practice-in-tropical-countries-part-1.pdf [Internet]. [cited 2017 Nov 14]. Available from: https://medicallabtechno.weebly.com/uploads/7/5/1/5/7515789/monica-cheesbrough-district-laboratory-practice-in-tropical-countries-part-1.pdf

24. Fodjo BAY, Atemnkeng N, Esemu L, Yuosembom EK, Quakyi IA, Tchinda VHM, et al. Antibody responses to the full-length VAR2CSA and its DBL domains in Cameroonian children and teenagers. Malar J [Internet]. 2016 [cited 2020 Feb 8];15. Available from: https://www.ncbi.nlm.nih.gov/pmc/articles/PMC5097422/

25. Babakhanyan A, Fang R, Wey A, Salanti A, Sama G, Efundem C, et al. Comparison of the specificity of antibodies to VAR2CSA in Cameroonian multigravidae with and without placental malaria: a retrospective case–control study. Malar J. 2015 Dec 1;14(1):480.

26. Lindholz CG, Favero V, Verissimo C de M, Candido RRF, Souza RP de, Santos RR dos, et al. Study of diagnostic accuracy of Helmintex, Kato-Katz, and POC-CCA methods for diagnosing intestinal schistosomiasis in Candeal, a low intensity transmission area in northeastern Brazil. PLoS Negl Trop Dis. 2018 Mar 8;12(3):e0006274.

27. Yanet F-S, Fidel-Angel N-F, Guillermo N, Sergio S-P. Comparison of parasitological techniques for the diagnosis of intestinal parasitic infections in patients with presumptive malabsorption. J Parasit Dis Off Organ Indian Soc Parasitol. 2017 Sep;41(3):718–22.

28. Haemoglobin_en.doc - haemoglobin WHO .pdf [Internet]. [cited 2017 Aug 21]. Available from: http://www.who.int/vmnis/indicators/haemoglobin.pdf

29. Hemoglobin Concentration (Hb): Reference Range, Interpretation, Collection and Panels. 2016 Jun 1 [cited 2017 May 18]; Available from: http://emedicine.medscape.com/article/2085614-overview

30. Degarege A, Veledar E, Degarege D, Erko B, Nacher M, Madhivanan P. Plasmodium falciparum and soil-transmitted helminth co-infections among children in sub-Saharan Africa: a systematic review and meta-analysis. Parasit Vectors [Internet]. 2016 Jun 15 [cited 2020 Feb 11];9. Available from: https://www.ncbi.nlm.nih.gov/pmc/articles/PMC4908807/

31. Hürlimann E, Yapi RB, Houngbedji CA, Schmidlin T, Kouadio BA, Silué KD, et al. The epidemiology of polyparasitism and implications for morbidity in two rural communities of Côte d'Ivoire. Parasit Vectors. 2014 Feb 25;7:81.

**Formatted:** Font: (Default) Times New Roman
**Formatted:** Font: (Default) Times New Roman, 12 pt
**Formatted:** Font: (Default) Times New Roman
**Formatted:** Font: (Default) Times New Roman, 12 pt
**Formatted:** Font: (Default) Times New Roman
**Formatted:** Font: (Default) Times New Roman, 12 pt
**Formatted:** Font: (Default) Times New Roman
**Formatted:** Font: (Default) Times New Roman, 12 pt
**Formatted:** Font: (Default) Times New Roman
**Formatted:** Font: (Default) Times New Roman, 12 pt
**Formatted:** Font: (Default) Times New Roman
**Formatted:** Font: (Default) Times New Roman, 12 pt
**Formatted:** Font: (Default) Times New Roman
**Formatted:** Font: (Default) Times New Roman, 12 pt
**Formatted:** Font: (Default) Times New Roman
**Formatted:** Font: (Default) Times New Roman, 12 pt
**Formatted:** Font: (Default) Times New Roman
**Formatted:** Font: (Default) Times New Roman, 12 pt
**Formatted:** Font: (Default) Times New Roman
**Formatted:** Font: (Default) Times New Roman, 12 pt
**Formatted:** Font: (Default) Times New Roman
**Formatted:** Font: (Default) Times New Roman, 12 pt
**Formatted:** Font: (Default) Times New Roman
**Formatted:** Font: (Default) Times New Roman, 12 pt

32. Akinbo FO, Omoregie R, Mordi R, Okaka CE. Prevalence of Malaria and Anemia Among Young Children in a Tertiary Hospital in Benin City, Edo State, Nigeria. Fooyin J Health Sci. 2009 Nov 1;1(2):81–4.

33. Achonduh-Atijegbe OA, Mfuh KO, Mbange AHE, Chedjou JP, Taylor DW, Nerurkar VR, et al. Prevalence of malaria, typhoid, toxoplasmosis and rubella among febrile children in Cameroon. BMC Infect Dis. 2016 Nov 8;16(1):658.

34. ARTAVANIS-TSAKONAS K, TONGREN JE, RILEY EM. The war between the malaria parasite and the immune system: immunity, immunoregulation and immunopathology. Clin Exp Immunol. 2003 Aug;133(2):145–52.

35. Roetynck S, Baratin M, Vivier É, Ugolini S. Cellules natural killer et immunité innée contre le paludisme. médecine/sciences. 2006 Aug 1;22(8–9):739–44.

36. Griffin JT, Hollingsworth TD, Reyburn H, Drakeley CJ, Riley EM, Ghani AC. Gradual acquisition of immunity to severe malaria with increasing exposure. Proc R Soc B Biol Sci [Internet]. 2015 Feb 22 [cited 2020 Feb 11];282(1801). Available from: https://www.ncbi.nlm.nih.gov/pmc/articles/PMC4309004/

37. Bediako Y, Adams R, Reid AJ, Valletta JJ, Ndungu FM, Sodenkamp J, et al. Repeated clinical malaria episodes are associated with modification of the immune system in children. BMC Med [Internet]. 2019 Mar 13 [cited 2020 Feb 11];17. Available from: https://www.ncbi.nlm.nih.gov/pmc/articles/PMC6415347/

38. Haldar K, Mohandas N. Malaria, erythrocytic infection, and anemia. Hematol Am Soc Hematol Educ Program. 2009;87–93.

39. World Malaria Report 2016 (annexes) - WMR-2016-annexes.pdf [Internet]. [cited 2017 May 1]. Available from: http://www.who.int/malaria/publications/world-malaria-report-2016/WMR-2016-annexes.pdf

40. Kovalszki A, Weller PF. Eosinophilia. Prim Care. 2016 Dec;43(4):607–17.

41. O'Connell EM, Nutman TB. Eosinophilia in Infectious Diseases. Immunol Allergy Clin North Am. 2015 Aug;35(3):493–522.

42. LYKE KE, DICKO A, DABO A, SANGARE L, KONE A, COULIBALY D, et al. ASSOCIATION OF SCHISTOSOMA HAEMATOBIUM INFECTION WITH PROTECTION AGAINST ACUTE PLASMODIUM FALCIPARUM MALARIA IN MALIAN CHILDREN. Am J Trop Med Hyg. 2005 Dec;73(6):1124–30.

43. Melo GC, Reyes-Lecca RC, Vitor-Silva S, Monteiro WM, Martins M, Benzecry SG, et al. Concurrent Helminthic Infection Protects Schoolchildren with Plasmodium vivax from Anemia. PLOS ONE. 2010 Jun 21;5(6):e11206.

44. Giardia: Overview and Update [Internet]. [cited 2017 May 16]. Available from: https://oup.silverchair-cdn.com/oup/backfile/Content_public/Journal/cid/25/3/10.1086/513745/2/25-3-545.pdf?

45. Giardiasis: Background, Pathophysiology, Etiology. 2016 Dec 2 [cited 2017 May 16]; Available from: http://emedicine.medscape.com/article/176718-overview

46. Entamoeba histolytica - Overview [Internet]. Encyclopedia of Life. [cited 2017 May 16]. Available from: http://eol.org/pages/491174/overview

**Formatted:** Font: (Default) Times New Roman
**Formatted:** Font: (Default) Times New Roman, 12 pt
**Formatted:** Font: (Default) Times New Roman
**Formatted:** Font: (Default) Times New Roman, 12 pt
**Formatted:** Font: (Default) Times New Roman
**Formatted:** Font: (Default) Times New Roman, 12 pt
**Formatted:** Font: (Default) Times New Roman
**Formatted:** Font: (Default) Times New Roman, 12 pt
**Formatted:** Font: (Default) Times New Roman
**Formatted:** Font: (Default) Times New Roman, 12 pt
**Formatted:** Font: (Default) Times New Roman
**Formatted:** Font: (Default) Times New Roman, 12 pt
**Formatted:** Font: (Default) Times New Roman
**Formatted:** Font: (Default) Times New Roman, 12 pt
**Formatted:** Font: (Default) Times New Roman
**Formatted:** Font: (Default) Times New Roman, 12 pt
**Formatted:** Font: (Default) Times New Roman
**Formatted:** Font: (Default) Times New Roman, 12 pt
**Formatted:** Font: (Default) Times New Roman
**Formatted:** Font: (Default) Times New Roman, 12 pt
**Formatted:** Font: (Default) Times New Roman
**Formatted:** Font: (Default) Times New Roman, 12 pt
**Formatted:** Font: (Default) Times New Roman
**Formatted:** Font: (Default) Times New Roman, 12 pt
**Formatted:** Font: (Default) Times New Roman
**Formatted:** Font: (Default) Times New Roman, 12 pt
**Formatted:** Font: (Default) Times New Roman
**Formatted:** Font: (Default) Times New Roman, 12 pt
**Formatted:** Font: (Default) Times New Roman
**Formatted:** Font: (Default) Times New Roman, 12 pt

47. Amebiasis: Background, Pathophysiology, Etiology. 2017 Jan 6 [cited 2017 May 16]; Available from: http://emedicine.medscape.com/article/212029-overview

48. Richardson DJ, Callahan KD, Dondji B, Tsekeng P, Richardson KE. Prevalence of Waterborne Protozoan Parasites in Two Rural Villages in the West Province of Cameroon. Comp Parasitol. 2011 Jan;78(1):180–4.

49. Mbuh JV, Ntonifor NH, Ojong J. The epidemiology of soil-transmitted helminth and protozoan infections in south-west Cameroon. J Helminthol. 2012 Mar;86(1):30–7.

50. Mbuh JV, Ntonifor HN, Ojong JT. The incidence, intensity and host morbidity of human parasitic protozoan infections in gastrointestinal disorder outpatients in Buea Sub Division, Cameroon. J Infect Dev Ctries. 2009 Dec 28;4(1):38–43.

51. Kuete T, Yemeli FLS, Mvoa EE, Nkoa T, Somo RM, Ekobo AS. Prevalence and Risk Factors of Intestinal Helminth and Protozoa Infections in an Urban Setting of Cameroon: the Case of Douala. Am J Epidemiol Infect Dis Am J Epidemiol Infect Dis. 2015 Jun 16;3(2):36–44.

52. Diallo TO, Remoue F, Gaayeb L, Schacht A-M, Charrier N, Clerck DD, et al. Schistosomiasis Coinfection in Children Influences Acquired Immune Response against Plasmodium falciparum Malaria Antigens. PLOS ONE. 2010 Sep 15;5(9):e12764.

53. Courtin D, Djilali-Saïah A, Milet J, Soulard V, Gaye O, Migot-Nabias F, et al. Schistosoma haematobium infection affects Plasmodium falciparum-specific IgG responses associated with protection against malaria. Parasite Immunol. 2011 Feb 1;33(2):124–31.

54. Hartgers FC, Obeng BB, Kruize YCM, Dijkhuis A, McCall M, Sauerwein RW, et al. Responses to Malarial Antigens Are Altered in Helminth-Infected Children. J Infect Dis. 199:1528–35.

55. Su Z, Segura M, Stevenson MM. Reduced Protective Efficacy of a Blood-Stage Malaria Vaccine by Concurrent Nematode Infection. Infect Immun. 2006 Apr 1;74(4):2138–44.

56. Lemaitre M, Watier L, Briand V, Garcia A, Hesran JYL, Cot M. Coinfection with Plasmodium falciparum and Schistosoma haematobium: Additional Evidence of the Protective Effect of Schistosomiasis on Malaria in Senegalese Children. Am J Trop Med Hyg. 2014 Feb 5;90(2):329–34.

57. Entamoeba histolytica: Host Defense and Immune Responses [Internet]. http://www.eurekaselect.com. [cited 2020 Feb 11]. Available from: http://www.eurekaselect.com/54531/chapter

Rose F. G. Leke, Jude D. Bioga, James Zhou, Genevieve G. Fouda, Robert J. I. Leke, Viviane Tchinda, Rosette Megnekou, Josephine Fogako, Grace Sama, Philomina Gwanmesia, Germaine Bomback, Charles Nama, Ababacar Diouf, Naveen Bobbili, Diane Wallace Taylor. Longitudinal Studies of *Plasmodium falciparum* Malaria in Pregnant Women Living in a Rural Cameroonian Village with High Perennial Transmission. Am J Trop Med Hyg. 2010 Nov 5; 83(5): 996–1004. doi: 10.4269/ajtmh.2010.10-0249 PMCID: PMC2963958

**Formatted:** Font: (Default) Times New Roman
**Formatted:** Font: (Default) Times New Roman, 12 pt
**Formatted:** Font: (Default) Times New Roman
**Formatted:** Font: (Default) Times New Roman, 12 pt
**Formatted:** Font: (Default) Times New Roman
**Formatted:** Font: (Default) Times New Roman, 12 pt
**Formatted:** Font: (Default) Times New Roman
**Formatted:** Font: (Default) Times New Roman, 12 pt
**Formatted:** Font: (Default) Times New Roman
**Formatted:** Font: (Default) Times New Roman, 12 pt
**Formatted:** Font: (Default) Times New Roman
**Formatted:** Font: (Default) Times New Roman, 12 pt
**Formatted:** Font: (Default) Times New Roman
**Formatted:** Font: (Default) Times New Roman, 12 pt
**Formatted:** Font: (Default) Times New Roman
**Formatted:** Font: (Default) Cambria Math
**Formatted:** Bibliography
**Formatted:** Bibliography, Pattern: Clear

**Reviewer #1**: Dr. Mbe-cho and colleagues sought to determine the prevalence of co-infection of malaria and intestinal parasites and its association with antibody levels to malaria merozoite antigens. The authors report that there was no difference in antibody prevalence or levels in malaria-infected and co-infected children, except antibody levels to EBA-175 were significantly higher in children co-infected with malaria and E. histolytica. Overall, the study is well-designed but these results do not significantly alter or impact our understanding of the association of malaria and helminths on antibody to malaria merozoite antigens.

1. The limitation of the study is that the parasite testing in children was not followed by sensitive diagnostic techniques like PCR, and light infections may have been missed which may have resulted in misclassification of the groups. Light infections may boost the antibody responses while children remain asymptomatic.
*Reply: We understand the concern. When the study was conducted (2017) in the rural villages, the prevalence of slide-positive malaria was 75.6%. In a prior study conducted in the village (Leke et al 2010), an equivalent prevalence was found of P. falciparum (50-85%) in children aged 5-15 years over a 5-year period. The estimated entomological inoculation rate (EIR) was 0.7 infectious bites/person/ nightly thought out the year (~257 IB/P/Y). Based on the more recent malaria prevalence, it appears that the current EIR is similar. Thus, children were most likely being bitten approximately every-other night by an infectious mosquito, since bednets were not routinely used. With this high level of transmission, most of the slide-negative children would be PCR-positive for malaria, i.e., have enough immunity to reduce malaria to submicroscopic levels. Unfortunately, in very high transmission areas like the one reported herein, everyone will have some circulating P. falciparum parasites. So, classifying subjects as slide-positive vs slide-negative may not reflect presence/absence of parasites, but provide information on the immune status of the person. In revising the MS, information from the study by Leke et al. was included as well as a discussion of submicroscopic infections in the revised Discussion.*

2. In this study, only 3.4% children were infected with helminths alone to get any meaningful data for antibody response to malaria in this group.
*Reply: We agree, the sample size of children with helminth infections is too small to provide meaningful information. Accordingly, Ab levels in children with helminth infections were not analyzed. To explain the low prevalence of helminths, information on the Ministry of Heatlth's policy for biannual treatment of children for worms was provided.*

3. Very few children are positive for E. histolytica.
*Reply: True, the prevalence of Entamoeba in our study was only 5.9%, which is lower than that reported in studies in these areas of ~23% (T. E. Kwenti et al., 2016). In our study, the prevalence was lower, probably due to rigorous mass drug administration (MDA) programs implemented by the Ministry of Health and other regular or seasonal health campaigns.*

4. The data on the children's anthropomorphic measurements are not mentioned. Thus, there is not much point describing how they were collected.
*Reply: This section was removed from the Methods section.*

5. There is no data on hookworm infection in the results.
*Reply: The prevalence of hookworm infections was considered in this study during stool exams and, surprisingly, we did not find hookworms in the samples collected, most likely due to regular deworming and improved hygiene in the area. No invasive methods were used for diagnosis of adult worms. From a paper published by E. Kwenti et al. (2016) the prevalence of hookworm was 7% in south west region Cameroon.*

6. The number of eggs per gram of stool were estimated for the parasites listed. Did the authors look at the responses in children with high or low intensity of the parasites?
*Reply: In this study, after obtaining the prevalence of parasites and comparing with antibody response, no significant difference was observed between the malaria antibodies levels and parasites eggs counts.*

7. Table 2 is not necessary, it can be written as text.
*Reply: Thanks for the comment, but we think Table 2 summarizes the data more clearly and allows readers to easily compare results from different groups than presenting them in the text. Table 2 has been revised.*

8. Page 21, reference # 54, year of publication is missing.
*Reply: Year of publication has been included.*

Please check spelling and typographical errors scattered through the manuscript (page and lines are given from word document):
1. Page 2, line 3, change led to lead in the sentence.
*Reply: The word "led" has been changed to "lead".*

2. Page 2, line 14, correct the spelling of Rietchi concentration method
*Reply: Spelling has been corrected to "Ritchie"*

3. Page 6, line 21: The bracket has to be closed here: (AB Leo Diagnostics, Helsingborg, Sweden.
*Reply: The bracket has been closed.*

4. Page 7, line 17 and 18: Correct 50ul to 50µl
*Reply: The change has been made.*

5. Page 9 and 10: In the text, the p value for anemia (MAL+,IP-) is p=0.034; p value for the same in Table 1 is p=0.032; it needs to be corrected.
*Reply: P value has been corrected to P=0.032 (correct value) in the text.*

6. Page 10: In Table 1, % sign is missing in column 5 for children with Hb.
*Reply: The % symbol has been included in table 1, column 5.*

7. Page 10, line 3: In the sentence, change major to majority.
*Reply: The word "major" has been changed to "majority".*

8. Page 14, line 27: In the sentence, MSL- should be MAL-
*Reply: In Line 27 of page 14, MSL- has been changed to MAL-*

9. Page 17, line 15: change beats to beads
*Reply: The spelling of beads has been corrected.*

10. Re-write the following sentences, they are not very clear:

Page 4, line 8:
However, with most children getting infected with several episodes of infections in a short period, this renders them more prone to having clinical symptoms since the immune systems doesn't fully recover.

*Reply: The sentence has been deleted because the information is not directly relevant to the study.*

Page 4, line 20:
Concomitant infections in humans have suggested that Ascaris lumbricoides infection may protect against cerebral malaria (11,12), while other studies, children infected by S. mansoni were more susceptible to P. falciparum infection and develop acute malaria episodes.
*Reply: The sentence has been revised to read: "Studies on concomitant infections in humans suggest that A. lumbricoides infection may protect against cerebral malaria (11,12), while other studies suggest that children infected by S. mansoni may be more susceptible to P. falciparum infections and develop acute malaria episodes (13,14)."*

Page 15, line 3:
In essence, the immune response in individuals who are repeatedly infection would be similar to that produce during chronic infections.
*Reply: To clarify the statement, the text has been revised to read: "Because of high transmission, the children are becoming infected almost daily and are either in the process of eliminating the new infection or reducing it to a submicroscopic level. Because of constant re-exposure, the resulting immune response will be similar to that produced by a chronic infection.*

**Reviewer #2:** The answer to the questions is divided into Major comments, Minor comments. Additionally, I wrote minor observations that, I hope, will help this manuscript to improve readability and consistency.

1. Is the manuscript technically sound, and do the data support the conclusions?
2. Has the statistical analysis been performed appropriately and rigorously?
3. Have the authors made all data underlying the findings in their manuscript fully available?
4. Is the manuscript presented in an intelligible fashion and written in standard English?

Major comments:

• Given that there were no differences in the IgG response between age groups, it would be interesting to join these data, evaluate all the coinfected individuals, and then split the data into Giardia, E. hystolitica.
*Reply: We are confused by this comment, because Fig 1 shows an increase in both Ab prevalence (Fig. 1A) and Ab levels (Fig 1 B-E) with age in Ab-positive children (Kruskal-Wallis test p values were p<0.001 MSP2 and p=0.05-0.086 (borderline) for the other antigens).*

*We believe combining all MAL+,IP+ children into single a group is unwise, since they were infected with a conglomerate of intestinal helminths, cestodes and protozoa (see Table 2). Combining children with such heterogenous infections is unlikely to provide meaningful information.*

• I strongly suggest dividing the age of individuals in 0-5, 5-10, 10-15 years-old to partially solve the "N" problem of the groups.
*Reply: Thanks for the comment. Initially, children were groups into 5-year categories as suggested by the Reviewer, i.e., 0-5, 5-10, 10-15 years old. However, when the data set showed that children aged 1 to 2 did not have intestinal parasites, the results were grouped into 2-year intervals, that allowed us to more closely define the increase in Ab prevalence (Fig. 1A) and Ab levels (Fig 1 -B,C,D,E) with age. The purpose of Fig 1 was to determine if age was a variable that needed to be taken into consideration during data analysis.*

• Because of the absence of molecular Diagnosis and considering that the authors mention the possibility of oh having low parasitemia infections in the MAL- group. It is important to include MAL- individuals in Figure 1.
*Reply: We are sorry if we didn't make the point clear. ALL children who were Ab-positive are included in Fig 1, including those who are MAL+ and* ***MAL-****. Because malaria transmission is high in the area, all children in the study had been exposed to P. falciparum and many of the MAL- children were Ab-positive.*

• It is necessary to compare parasite data with similar regions in Cameroon. Please compare and cite:
• (Malaria and Helminth Co-Infection in Children Living in a Malaria Endemic Setting of Mount Cameroon and Predictors of Anemia from Theresa K Nkuo-Akenji et al. 2006)
• Malaria, Helminths, Coinfection and Anaemia in a Cohort of Children From Mutengene, South Western Cameroon from Clarisse Njua-Yafi et al. 2016.
*Reply: We thank the Reviewer for pointing out the omission of key references. Information from these studies have been included in the revised Discussion. The text now reads, ".....to those found in other highly [malaria] endemic regions of the country (32), and the prevalence of co-infections was 19.1%, which is similar to the prevalence of co-infections of 18 – 27% reported in other regions of Cameroon (9,44).* The references have been added to the reference section.

• Do the authors have information about malaria and intestinal parasites last treatments? On page 17, it was commented that Albendazole treatment was frequent in these children. Deworming information will help the readers to understand why the prevalence of intestinal parasites was low compared with other studies in Cameroon. Additionally, reinforce in the discussion section that collecting/reporting that information is valuable for coinfection studies.
*Reply: In response to the Reviewer's suggestion, the following information has been added to the Methods section. "Currently, mass drug administration with albendazole is being performed twice a year by the Ministry of Health, that is usually conduced in schools and symptomatic cases are sent to the local clinic or hospital for follow up treatment."*

• (Figure 1 B, C, D, E) use the same scale limits for all plots. This is also useful to understand differences in levels of antigenicity between proteins.
*Reply: We understand the comment, but we do not wish to change the Y-axis on Fig 1, since it is risky to make a direct comparison of Ab levels between antigens in serological assays. A number of variables, including parasite strain, the system to produce recombinant proteins, protein purity, the amount of antigen used, number of exposed epitopes, dilution of plasma, etc., influence the overall results. Even when Luminex beads are covalently-coupled with saturating amounts of antigen, it is questionable if direct comparison of MFI can be made between antigens. Although our assays have been optimized and equivalence amounts of antigen used during bead-coupling, comparisons among the antigens may not provide accurate information about immunogenicity. In Figs1 B, C, D, E, the Y-Axis was selected to show the best distribution of the MFI results.*

• (table 3) How could the authors explain increased eosinophilia with low levels of helminth infection? This mainly applies to the age group > 9 years-old.
*Reply: After age 2, children start becoming infected with helminths, resulting in an increase in eosinophil counts. During the biannual drug treatment campaign, helminthic infections are eliminated, but eosinophilia persists for a period of time. With increasing age, more children in the area become i) infected and ii) re-infected, resulting in an increase in prevalence of eosinophilia.*

• (Page 17) The authors argue, "First, children living in moist or wet environments where mosquitoes breed and E. histolytica are more abundant would have a high risk of acquiring both infections, that would result in frequent boosting of the Ab response." This explanation for intestinal parasite influence on antibody production alteration is not viable since Giardia's frequency is higher than E. histolytica in the studied population.
*Reply: The sentence has been deleted from the Discussion.*

• (Page 17) The affirmation "Secondly, since malaria and E. histolytica are both amoebae, they might share common antigens, for example, EBA-175 could share homology with an E. histolytica antigen." is false. Plasmodium falciparum is not an is a protozoan. This group belongs to Apicomplexa organisms. For that reason, the hypothesis about correlating Plasmodium and E. histolytic is wrong.
*Sorry, "amoebae" was a typo. Both Plasmodium falciparum and E. histolytica are protozoans. The Discussion has been revised to read "parasitic protozoa."*

• How different are the two Villages Ngali II and Mfou in the central region of Cameroon? Does it exist a difference in humidity and soil moist, once the authors claimed that this variable could explain differences of Entamoeba histolytica?
*Reply: The two villages are very similar with no major differences in humidity or soil moisture. The estimated annual average rainfall measures 1600 $mm^3$ with an annual average temperature of 23°C for Ngali II and for Mfou. According to the National Meteorology agency, the average humidity for the center regions is 83%. Ngali and Mfou are both in the center region of Cameroon about 60km apart. Note: as mentioned above, the words "humidity and soil moisture" have been deleted from the MS.*

Minor comments:
• What criteria were used to divide the population into seven groups according to age?
*Reply: The fact that Intestinal parasite (IP) infections was only observed in children >2 years, helped guide separation of the children into seven groups.*

• Please specify how anthropometric parameters were used in the study, once they were described but not used in the study. If this information was not used, please remove these sentences.
*Reply: The sentence has been removed.*

• Has the studied region presence of Schistosoma haematobium? If the authors have register if this parasite in the area, Did they examined urine samples to discard infections with this parasite?
*Reply: Detection of S. haematobium was not included in the study design because of low prevalence in the study area. A study conducted in this area (and other regions of Cameroon) by Louis-Albert Tchuem Tchuenté et al., (2012) reported a prevalence of S. haematobium of only 1.72%. Since a large sample size would be required to assess the impact of this pathogen on the Ab response to malaria, S. haematobium was not included in the study.*

• Were the individuals asymptomatic to intestinal parasites infection too? No diarrhea, abdominal pain, etc.? Please clarify.
*Reply: Yes. To make the point clear, the Methods section has been revised and states that all children with clinical cases of malaria or intestinal parasites were not included in the study and referred to the local clinic/hospital by the attending physician for treatment. Thank you for the comment.*

• (Page 6) It was mentioned that Plasmodium parasitemia was quantified. Did the authors observe any correlation between the Plasmodium parasite burden and the levels of IgG responses to the antigens?
*Reply: As expected, there was no correlation between parasitemia and malaria antibody levels.*

• (End of Page 7) Please specify: If the cut-off is MFI+3*SD, how the standard deviation was calculated if the negative controls were pooled? Was this experiment repeated or used replicates? Traditionally, the negative controls are tested simultaneously in different wells of the plate, and the cut-off is calculated from those values.
*Reply: Pooled negative control plasma sample were run in triplicates on the same plates as the test samples in all experiments, as well as the positive controls. The cut-off was obtained by calculating MFI+3 SD of the triplicates on all plates in the experiment.*

• Did the authors analyze the effect of helminth parasite burden (number of eggs/gram of stool) in those individuals with helminths? This valuable information was commented on but never included in the analysis. If not used,e I do not see the necessity of describing in the methods section
*Reply: The information has been deleted from the Methods section.*

• For data analysis:
• Before using ANOVA, did the authors checked for the normality of the variables? If yes, please specify, if not, calculate the normality of the variables and the other ANOVA assumptions.
*Reply: Yes, ANOVA was used to compare difference in age across the 4 groups (Table 2). However, comparisons of Ab MFI, which are not normally distributed, with age (Fig. 1) were performed using the Kruskal-Wallis test. The Methods section (Data analysis) has been revised. Information in Fig. 1 legend was correct.*

• If the authors have not-normal variables, they should use the Kruskal-Wallis non-parametric, and Dunn posthoc tests to verify differences between groups.
*Reply: Sorry for the mistake in the Methods section. The Kruskal-Wallis nonparametric test was performed in Fig 1 and 2. A posthoc test was not performed, as the goal was not to determine when peak Ab levels were obtained, but to determine if age had an influence on Ab levels. Since age was a variable, data for all age groups could not be combined, but rather age was taken into consideration during data analysis.*

• Please check frequencies described in table 1 (MAL+IP- 58.8%) vs. the values reported in the second line page 9. (59.4%).
*Reply: 59.4% is the correct value. The text has been revised.*

• Sum of 58.8%+16.9% = 75.7% not 75.6%.
*Reply: Thank you for catching the error. The values in Table 1 and text have been revised and are now consistent.*

• In table 1, please add a column with P-values to facilitate the interpretation of the differences between groups. Please report statistics of multiple comparisons between groups too.
*Reply: The comparisons requested by the reviewer were originally provided in the Table legend. To comply with the request, the p values have been moved to a column labeled "p values" and the method of analysis was retained in the Table legend.*

• What is the potential hypothesis to explain the increased values of parasitemia in the coinfected group?
*Reply: There is no significant difference in parasitemia between the two groups (p=0.1599). In fact, the higher parasitemia was found in young children who were intestinal parasite-negative (probably because very young children were in this group).*

• Please comment in the text the presence of multi-parasitism in the studied individuals.
*Reply: We thank the reviewer for the comment. The following sentence has been added to the Results section. "Interestingly, all of the children had single parasite infections, and polyparasitism was not found."*

• (Page 11 table 3). Please include values of anemia and eosinophilia in individuals coinfected. In the current configuration is constructed is hard to determine the coinfection impact in anemia and eosinophilia values.
*Reply: Table 3 was designed to evaluate the influence of **age** on malaria, IP, anemia and eosinophilia. The number of co-infections are too small to be divided by age. In an attempt to address the Reviewer's comment, a separate Table was designed that compares the influence of no infections, malaria-positive only, and co-infections on percent with anemia and eosinophilia. The Table will be up-loaded as supplemental Table 1. It essentially showed that same results as expected, anemia was associated with malaria and eosinophils were associated with co-infections.*

• (Page 11). In the sentence, "Thus, as children living in these villages increased with age, they developed partial immunity to malaria and anemia declined; whereas, the prevalence of IP and eosinophilia increased." In this sentence, it is necessary to specify that "protection" is protection against malaria symptoms. The table clearly shows that the frequency of malaria does not decrease with age, only the anemia.
*Reply: The sentence has been revised to read: "Thus, as children living in these villages increased with age, they began developing partial immunity to malaria symptoms and anemia declined; whereas, the prevalence of IP and eosinophilia increased.*

• Please plot Age vs. Antibody levels for each protein to verify the correlation for each protein studied.
*Reply: The figure on the right confirms that Ab levels increase with age. The figure shows a linear regression analysis of Ab levels for MSP1, MSP2, MSP3 and EBA-175 using data from all 320 children, and includes the equation for the regression line, the $R^2$ value (all positive), and p value (all significant). Thus, the figure confirms that Ab levels increase with age. We do NOT wish to include this figure in the MS since it is essentially identical to the one shown in Fig 1 B, C, D and E. In fact, we feel that the information in Fig 1B-E is easier for the reader to understand.*
*Note: If the figure is not shown, it is provided in a separate document.*

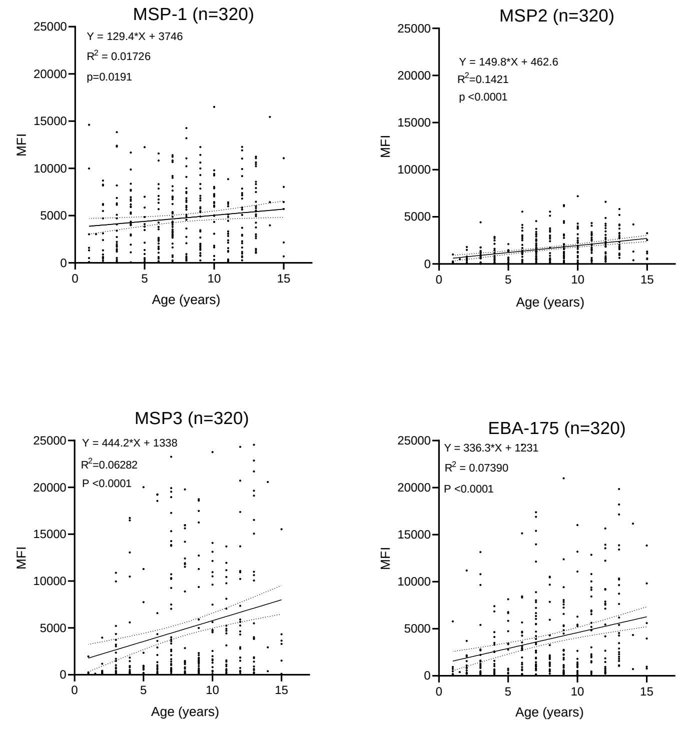


**D**istribution of MFI for all 320 children by age. Figure show the regression line +/- 95% CI.
These data confirm that between the ages of 1 to 15 years, the amount of Ab increases with age, as the results of increasing Ab prevalence and Ab levels.

• As an exploratory analysis, I suggest joining all data and make a boxplot comparing MFI between MAl-PI-, MAL-PI+, MAL+PI-, and MAL+PI+. Mainly for MSP1, MPS2, and MSP3 group age 3-10 and 11-15 to check.

*Reply: We thank the Reviewer Thanks for the suggestion concerning exploratory analysis. A comparison of Ab levels in two of the above groups (MAL-,IP-, and MAL+,IP-) is shown in Fig 2. Unfortunately, the number of children in the MAL-,PI+ group is too small to provide valuable information. As stated above, children in the MAL-,PI+ group (n=54) are infected with a variety of intestinal helminths, cestodes and protozoa (see Table 2). With such a diverse range of pathogens, plotting the data as a boxplot will not provide useful information. In Fig. 2, the distribution of Ab levels in children co-infected with malaria and single intestinal pathogens is provided. We feel this approach is more informative than "dumping all pathogens together."*

• The sentence "E. histolytica is a gut amoeba that causes both intestinal and extraintestinal infections such as amebic colitis (dysentery) and liver or brain abscess. The protozoa cause a marked down-regulation of macrophage functions rendering the cells incapable of antigen presentation and unresponsive to cytokine stimulation (57)" does not explain the increase of antibody production in E. histolytica infected group. Why could a diminishing antigen presentation generate higher levels of anti-Plasmodium antigens?
*Reply: Very true! Not sure why that statement wasn't caught. The Discussion has been changed significantly. It now reads, "The decrease in macrophage function does not explain the increase in Ab to EBA-175. One possible explanation is that since malaria and E. histolytica…"*

Other observations/questions:
• In the title, add "IgG" to Antibody response. *Reply: IgG has been added to title (although not all of the co-authors agree this is necessary).*

• Check all scientific names of parasite species for correct formatting in italics. (Example Entamoeba histolytica in the Results section in the abstract)
*Reply: The scientific name has been checked and are now in italics.*

• Please, mention in the background the region where the study was performed.
*Reply: This information was included in the background section of the Abstract. It is also included in the Materials section.*

• It is necessary to describe and discuss the role of MSP1, MPS2, MSP3, and EBA-175 as markers in serological studies.
*Reply: This information has been added to the Discussion.*

• Considering that coinfection prevalence is relatively low, I consider that it is important to discriminate with colors or point shapes the individuals MAL-IP-, MAL+IP-, MAL-IP+, MAL+IP+ in Figure 1 B-C-D-E
*Reply: We thank the Reviewer for the suggestion. However, information in Fig 1B-E is designed to address the question, are Ab prevalence and levels influence by age? Whereas, Fig 2 provides comparisons between individuals infected with malaria alone or co-infected with specific intestinal parasites. Thus, colored dots or symbols are not needed in Fig 1 (and could be confusing to the reader).*

• In page 6 subtitle "Laboratory detection, quantification and speciation of malaria parasites.", I will not use speciation here. I suggest "Diagnosis and quantification of Plasmodium sp. parasites.
*Reply: The header has been changed to read: "Laboratory detection of malaria parasites."*

• (Page 14-15) What type of parasite is "Amoeba"? What is the difference between "Amoeba" and E. histolytica? Traditionally, E. histolytica is considered an amoeba too.

*Reply: The figure has been revised to read Intestinal Protozoa. Thanks for pointing out the mis-classification.*

• In table 1, to facilitate reading, please remove symbols % and /ul located in cells with data and add to the columns describing the variables.
*Reply: The symbols in the data cells have been removed.*

• For consistency, unify parasitemia vs. parasitemia, anemia vs. anemia in the text and plots.
*Reply: The British spelling of parasitaemia, anaemia, and haemoglobin have been used through out the MS.*

• (Page 10) change "The major of helminth parasites" to "The most frequent helminth species detected."
*Reply: The change was made as suggested.*

• (Table 2) Check all the total numbers for the "Total IP+" column. For example, for protozoans, the sum is 29+19+4 = 48, and it was reported 47
*Reply: This has been verified and corrected to 48 in Table 2*

• (Page 13) In plot titles Change Ab (Antibody) to IgG
*Reply: We thank the Reviewer for the comment, but decide not to make the change. Our rationale is that by definition, IgG is a class of immunoglobulin found in the blood; whereas, Ab are plasma proteins that bind specifically with an antigen. What was measured was IgG Ab. Since the serological assay measured IgG Ab that were recorded as MFI (median fluorescence intensity), we think the labels on the Y-Axis (Ab levels -MFI) reflect what was done. The Methods section makes it clear that the Ab were of the IgG class. [Note: Serum IgG levels (which implies mg/ml) were not measured.]*

• (Figure 1E) Add, Change from EBA to EBA-175.
*Reply: Change has been made.*

• Please verify all references formatting (For example, reference 42 is all in capital letters)
*Reply: References have been edited as requested by the reviewer.*

**Review #3**: Comments were in the attachment.
*Reply: In revising the MS, all requested changes were made and additional information provided in the text, including information on the BLAST search. The only request we would not fully address is the prevalence of bednet use in the villages. The only information available is that very few children use bednets. Since the slide-positivity rate of 75.6% for P. falciparum, it is unlikely the bednets are having a major influence on the current study. The following information has been added to the MS in the Results section. "To determine if higher Ab levels in children co-infected with P. falciparum and E. histolytica might be due to cross-reactive epitopes, a BLAST search for sequence homology between EBA-175 and E. histolytica proteins was made. No similarities were found using Metablast, and only one hit was found using discontinuous metablast which had a span of only 38 nucleotides (~12 amino acids). Thus, there does not appear to be shared epitopes between these two pathogens that would explain the increase in Ab to EBA-175 in children with co-infections."*

**Figure for Reviewer #2 confirming an increase in antibody levels with age.**

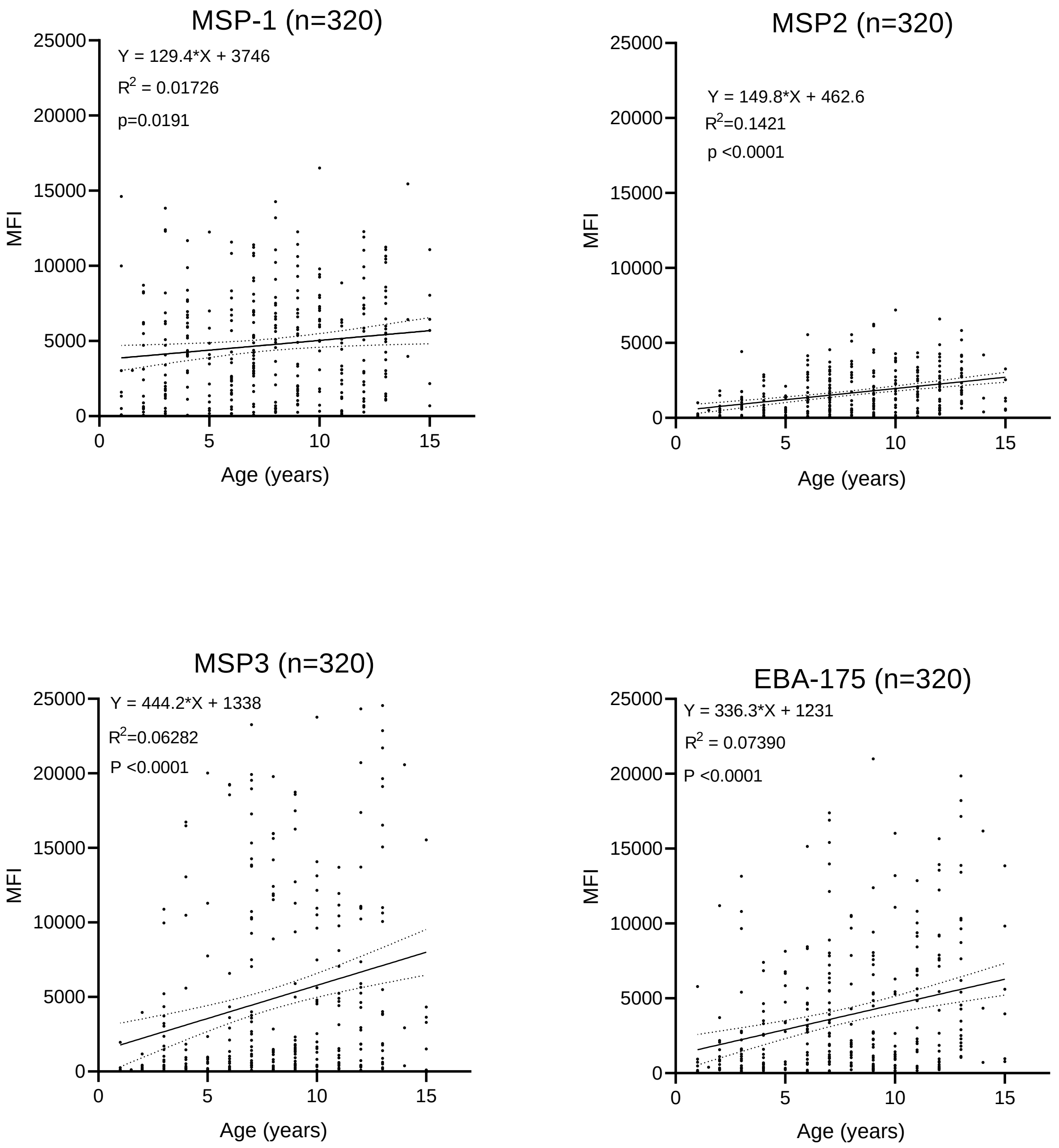


**D**istribution of MFI for all 320 children by age. Figure show the regression line +/- 95% CI. These data confirm that between the ages of 1 to 15 years, the amount of Ab increases with age, as the results of increasing Ab prevalence and Ab levels.
