# Supplementary material for: The immunoglobulin G antibody response to malaria merozoite antigens in asymptomatic children co-infected with malaria and intestinal parasites
Source: PLoS One. 2020 Nov 10;15(11):e0242012. doi: 10.1371/journal.pone.0242012 (PMC7654760; doi:10.1371/journal.pone.0242012)
Supplement: Attachment — Submitted filename: Final comments Reviewer 2.pdf [file pone.0242012.s007.pdf]

Reviewer #2: Comments:

1. Check carefully all the italics for species names
Reply: We thank the reviewer for this comment, and this has been checked and edited throughout the document.

A =/ OK. Thanks for the answer.

2. Is it necessary the sentence in the line 342 in italics?
Reply: We thank the reviewer for this comment. This statement was highlighted to emphasize the statement. The italic has been removed.

A =/ OK. Thanks for the answer

3. As I previously commented, the discussion about MSP1, MSP2, and MSP3 role as maskers is absent. Please discuss and compare findings with other studies using the same proteins.
Reply: Please forgive us for not making the change previously, but we are having difficulty understanding the comment in the context of the current study. Clearly, MSP1, MSP2, and MSP3 are considered as vaccine candidates, but, like all antibodies, Ab to MSP1, MSP2 and MSP3 are simply “markers” of exposure. Over the last 10 years, the presence of Ab to merozoite antigens, not only MSP1, MSP2 and MSP3, are considered to serve as markers for infection with malaria (Patel P et al 2017, Suman et al 2010). Likewise, we are confused about the request to “discuss and compare findings with other studies using the same proteins,” since reference to other studies that directly relate to the current study have been made. Since many studies have documented that Ab prevalence and levels increase with age, it seems unnecessary to provide multiple references to document this point. In an attempt to respond appropriately to the comments, a paragraph has been added to the Discussion. The paragraph reads: “This study measured the Ab response to 4 antigens present in P. falciparum merozoites. Following schizont rupture, the merozoite surface antigens (MSPs) participate in initial attachment of merozoites to erythrocytes; then erythrocyte-binding antigen EBA-175 aids in binding and induces release of proteins localized in the micronemes that participates in junction formation [Beeson et al. - review]. Thus, the immune system of children living in Etoudi, Cameroon, would be exposed to these antigens simultaneously and repeatedly. Although considered to be vaccine candidates, Ab against these antigens are markers of past or current infection and not necessarily markers of protection [Beeson et al]. Antibodies against any one of the antigens alone is not an index of immunity to malaria. Thus, even if intestinal parasitic pathogens had down-regulated humoral responses to P. falciparum, reduced Ab levels would not necessarily mean the individual had increased susceptibility to malaria disease. Studying these antigens, however, provided a way to assess the impact of intestinal pathogens on humoral immunity to asexual P. falciparum parasites.”

A =/ Since the authors could not test the hypothesis about the effect of coinfections in the antibody response due to the low frequency of helminths in the studied population, I was expecting more discussion about the antibody levels against MSP1, MSP2, and MSP3 proteins.
I wanted to see other manuscripts studying MSP1, MSP2, and MSP3 because I expected to see if similar levels of antibodies anti MSPs proteins in other populations/age groups, or even see comments about the association with protozoan coinfections. I know that these merozoite proteins are markers of parasite exposure, but at any moment the authors mentioned why those merozoite proteins were chosen over other Pf proteins (sporozoite or even sexual-stage). This type of information is essential, mainly for non-malaria researchers that will read the manuscript.
4. Please mention in results that hookworms were not detected in the study
Reply; thank the reviewer for this comment. It has been included accordingly in the text (line 232)

A =/ OK, thanks for the answer.

5. Please mention in results that no significant difference was observed between the malaria antibodies levels and parasites eggs counts.
Reply: Sorry, we humbly beg to differ with the Reviewer and have not complied with the suggestion. Since only 9 subjects had Ascaris lumbricoides infections, the sample size is simply too small to determine if an association exists between Ab levels and egg counts. That is, egg counts ranged only from 100 to 320 eggs per gram of stool; whereas, Ab levels (MFI) had a wide range: (median: 25th, 75th percentile: MSP1 - 2088: 696,1367, 7191; MSP2 - 1778:1507,2750; MSP3 - 6778:434,14,323; EBA-175 10,944:1486, 15058 MFI for these 9 individuals). Using a simple linear regression model, none of the p values were significant (p values = 0.19 to 0.80), demonstrating that none of the associations (regression lines) differed significantly from zero. Although, “technically” there was no statistical association between egg counts and Ab levels, we feel that drawing the conclusion that there was no association is invalid and not good science.

A =/ If the data is not analyzed or is insufficient for analysis and further discussion, it should be removed.

6. Although the authors specify two references describing the bead assay, I still believe it is important to report each antigen's concentration in the bead assay. Once the authors explained that these concentrations are not comparable. Additionally, in the references given (old 23 and 24), there is no information about how EBA175 was produced.
Reply: Information on EBA-175, along with the other antigens used, was provided in reference 23 (Fouda et al. 2006). Information on the concentrations and expression systems has been added to the Methods section. “Antigens used

included MSP142 of the FVO and 3D7 strains expressed in Escherichia coli and coupled at 0.2 μg per million beads, recombinant EBA-175 RII expressed in yeast coupled at 2.5 μg per million beads, recombinant MSP-3 C-terminal region expressed in E. coli coupled at 5 μg per million beads and recombinant MSP-2 (FC27 strain) coupled at 1 μg per million beads. (Fouda et al. 2006, Fodjio et al. 2016)."

A =/ OK, thanks for the answer.

7. Please add references showing that eosinophilia is maintained in individuals that had intestinal infections during a longer time even after MDA.
Reply: The only statement in the manuscript concerning eosinophils [line 248] is that … with age … "the prevalence of IP and eosinophilia increased." The statement simply reports the results. Thus, adding references doesn't seem appropriate. The length of time eosinophils persist after drug treatment is influenced by many variables, e.g., initial worm burden, number of eosinophils produced by the innate immune response, length of the infection prior to drug treatment, persistence of antigen, frequency of MDA, complete/incomplete cure, etc. In Etoudi, MDA was conducted approximately twice a year. Thus, some individuals became infected between MSD campaigns. Thus, providing references stating how long eosinophilia persists for an extended period after MDA is impossible to provide.

A=/ The authors should consider adding "The length of time eosinophils persist after drug treatment is influenced by many variables, e.g., initial worm burden, number of eosinophils produced by the innate immune response, length of the infection prior to drug treatment, persistence of antigen, frequency of MDA, complete/incomplete cure" as a complement to the eosinophils discussion and adding the reference. That was what I was expecting, discussion to complement the findings.

8. The author explains that "The fact that Intestinal parasite (IP) infections was only observed in children >2 years, helped guide separation of the children into seven groups." But that is not e real explanation; please explain what the rationale for that separation is? Why not use 3-4-5-6 groups?
Reply: We did use age groups 3-4, 5-6-year, etc. Since IP were not detected in children 1-2 years of age, it seemed logical to group children into 2-year bins. There was no other rationale. Grouping the children made it easier to graph the results. The same conclusion would be obtained if the children had been separated into larger sized groups or had not been grouped.

A=/ Thanks for the answer, I understand the point, and I recognize the need of grouping age because of the "n" for the groups. Only one comment here: Although the necessity of the groups, please consider that the immune system of the

children is changing and being challenged with aging and the cumulative exposure to parasites increases too, making this rational not that consistent.

9. Please comment in the abstract that the population received mass drug administration. I consider that that is a crucial point of the investigation and explains the lower prevalence of helminths and protozoans.
Reply: We thank the reviewer for this comment, and it has been included in the second line of the methods section on the abstract.

A=/ OK, thanks for the answer.

10. I think it would be necessary for the manuscript to verify the existence of other articles reporting intestinal parasites in populations under MDA treatment and add that to the discussion.
Reply: Mass drug administration (MDA) is a common practice in subtropical and tropical countries like Cameroon. The aim is to eliminate intestinal parasitic infections. However, complete eradication is usually not attained as other factors such as hygiene and sanitation, behavior of population and awareness are all require in an integrated manner to avert the problem as these infections may return after a MDA in as early as within four to six months (J.C. Dunn et al., 2019, G. Ortu et al., 2016). Adverse events when not well handled usually lead to rejection of MDA by certain families, thereby serving as reservoir for the parasites to continue to circulate in the community (P. Laurenzo et al., 2019). This information has been added to the Discussion as suggested. Line 385 to 388

A =/ Thanks for the answer. Could the authors please add that information as an additional comment to the results in the discussion section?
