# Supplementary material for: The immunoglobulin G antibody response to malaria merozoite antigens in asymptomatic children co-infected with malaria and intestinal parasites
Source: PLoS One. 2020 Nov 10;15(11):e0242012. doi: 10.1371/journal.pone.0242012 (PMC7654760; doi:10.1371/journal.pone.0242012)
Supplement: Attachment — Submitted filename: annotated review comments.docx [file pone.0242012.s003.docx]

**Full-title: Antibody Response to Malaria Merozoite Antigens in Asymptomatic Children Co-infected with Malaria and Intestinal Parasites**

**Running title: Malaria antibodies in children with intestinal parasites**

Authors: Ndiabamoh Crespo’o Mbe-cho^1,2*^, Gabriel L. Ekali^2,5^, Livo Esemu^2,3^, Yukie M. Lloyd ^2,4^ Wilfred Mbacham^1,2,3^, Jude D. Bigoga^2,3^ Diane W. Taylor^4^ and Rose G.F. Leke^2Ψ^

^1^Faculty of Medicine and Biomedical Sciences, University of Yaoundé I, Cameroon

^2^The Biotechnology Center, Nkolbisson, University of Yaoundé I, Cameroon

^3^Department of Biochemistry, University of Yaoundé 1, Cameroon

^4^John A. Burns School of Medicine, University of Hawai’i at Mānoa, USA

^5^National AIDS Control Committee, Ministry of Public Health, Cameroon

Correspondence to Rose Gana Fomban Leke ([roseleke@yahoo.com](mailto:roseleke@yahoo.com)) and Crespo’o Mbe-cho Ndiabamoh ([cndiabamoh@gmail.com](mailto:cndiabamoh@gmail.com))

**ABSTRACT**

Background

Co-infection with malaria and intestinal parasites is common in children in Africa and may affect their immune response to a malaria parasite infection. Prior studies suggest that co-infections may led to increased susceptibility to malaria infection and disease severity; however, other studies have shown the reverse. Knowledge on how co-morbidities specifically affect the immune response to malaria antigens is limited. Therefore, this study sought to determine the prevalence of co-infection of malaria and intestinal parasites and its association with antibody levels to malaria merozoite antigens.

Methods

A cross sectional study was carried out in two villages with high transmission of malaria in Cameroon (Ngali II and Mfou). Children aged 1-15 years were enrolled after obtaining parental consent. A malaria rapid diagnostic test was used on site. Four (4) ml of peripheral blood was collected from each participant for microscopy to determine Plasmodium infection and speciation, hemoglobin levels and serology. Fresh stool samples were collected and examined by wet mount, Kato-Katz method and modified Rietchi concentration techniques. A Multiplex Analyte Platform (MAP) assay was used to measure antibody levels.

Results

A total of 320 children were enrolled. The prevalence of malaria by blood smear was 199 (75.6%) and co-infections 54 (16.9%). Malaria prevalence was highest in young children; whereas, intestinal parasites (IP+) were not present until after 3 years of age. All children positive for malaria had antibodies to MSP1_42_, MSP2, MSP3 and EBA175. No difference in antibody levels in children with malaria-co infections compared to malaria alone were found, except for antibody levels to EBA-175 were higher in children co-infected with intestinal protozoa (amoeba) (p = 0.018), especially those with *Entamoeba histolytica* infections (p=0.0026).

Conclusion:

Antibody levels to EBA175 were significantly higher in children co-infected with malaria and *E. histolytica* compared to children infected with malaria alone. It is important to further investigate why and how the presence of these protozoans can modulate the immune response (Th1/Th2) to malaria antigens.

**Key words**: malaria, intestinal-parasites, antibody, *Giardia lamblia, Entamoeba histolytica*

**Background:**

In sub-Saharan Africa, malaria caused by *Plasmodium falciparum* (Pf) remains an important public health threat, killing over 292,000 children under the age of five each year (1,2). In malaria endemic areas, individuals exposed to malaria infections gradually develop clinical immunity (3) and commonly experience asymptomatic infections without fever or symptoms and do not require antimalarial treatment. Asymptomatic infection results from partial immunity that controls, but does not completely eliminate, malaria parasites, thus allowing for constant presence of circulating parasites (3). However, with most children getting infected with several episodes of infections in a short period, this renders them more prone to having clinical symptoms since the immune systems doesn’t fully recover.

The prevalence of intestinal parasitic infections in children is fairly constant across sub-Saharan Africa with an average prevalence of 26% (4,5). In Cameroon, the prevalence in children less than 18 years is 26.8% (6), while that for the general population is more than 28% The major soil-transmitted intestinal parasites are A*. lumbricoides, T. trichuria and E. histolytica* (7–9), but many cases of intestinal parasites go undetected.

Co-infections with malaria and intestinal parasites (IP) are common in malaria endemic areas in sub-Saharan Africa (8,9) and infections with IP and Pf are both ranked among the major cause of mortality and morbidity in sub-Saharan Africa. Several studies conducted on IP (not including amoebas) and Pf have shown conflicting results. Some helminths suppress different T-helper types and favor an increase in regulatory T (Treg) cell (10). Concomitant infections in humans have suggested that *Ascaris lumbricoides* infection may protect against cerebral malaria (11,12), while other studies, children infected by *S. mansoni* were more susceptible to P. falciparum infection and develop acute malaria episodes (13,14). Also, it has been shown that the levels of TNF-α, IL-2, IL-10, IL-6 in *Plasmodium*-helminth co-infected individuals were significantly higher than the MP group (15) dampening the immune response to malaria. However, little is known regarding host immune responses to malaria in children co-infected with amoebas.

Studies suggest that children co-infected with malaria and intestinal helminths had significantly decreased antibody levels to the malarial antigen apical merozoite antigen 1 (AMA-1) compared to those with *P. falciparum* or IP alone(16). Hence, infections with intestinal helminths can stifle protective anti-plasmodial antibody responses (16). However, increase in MSP3 IgG1–4 levels were significantly associated with children infected with malaria alone compared to children co-infected with both parasites(16).

Malaria and other intestinal parasites overlap extensively in their epidemiological distributions causing polyparasitism. Polyparasitism with intestinal parasites has been reported as one of the contributing factors to hypo-responsiveness (17), dampening of the immune response by inducing a strong Treg response, which could in turn, blunt a strong response to vaccines (18). Equally, some studies have suggested an effect of IP on antibody responses to Pf gametocyte antigens that may have consequences on transmission-blocking immunity (19).

Effective elimination and future eradication of malaria will require not only vector control, but also managing asymptomatic malaria patients and developing an effective vaccine. Given the high burden and concomitant nature of both malaria and intestinal parasites in the same geographical setting, conflicting data shows polyparasitism could interfere with the efficacy of malaria vaccines (20). To our knowledge, since limited information is available on whether and how co-infections of intestinal parasites and malaria affect the specific immune response to malaria antigens (21), the goal of this study was to investigate the prevalence and relationship between co-infections of malaria (MAL+) and intestinal parasites (IP+) (nematodes, trematodes, and protozoans) on naturally acquired antibodies to malaria merozoite .

**Methods:**

**Study Area description:**

The study was conducted in Ngali II and Mfou, two villages in the centeral region of Cameroon (located at 4°27′N and 11°38′E with a total population of about 1,000 children per squared Km (about 4000 in Ngali II and 6000 in Mfou) under the age of 15 years. The climate is typically equatorial with two discontinuous dry and rainy seasons. The annual average rainfall measures about 1600 mm^3^ with an annual average temperature of 23°C (22).

Most children in Ngali II and Mfou over 3 years of age accompany their parents to the farm and return home late at night. They seldom sleep under mosquito bed nets. The two villages are geographically similar, residents are relatively poor and have minimal access to portable water, with approximately one well per 500 inhabitants.

**Study Population**

A cross sectional study was carried out in Ngali II and Mfou from January to May 2017, a transitional period from the dry to wet season. Children who had lived in either of these two villages for at least six months and whose parents gave informed consent were included in the study. A total of 320 participants (140 from Ngali II and 180 from Mfou) aged 1-15 years participated in the study and were divided into 7 age groups (with a two years per group).. Since both villages (Ngali II & Mfou) were very similar in all features, data for both village were combined. Vital parameters (temperature, pulse) and anthropometric parameters (weight, height) were measured by assisting attendant nurses. These parameters were used to calculate body mass index (BMI) and advice was given to the parents of the participating children, as part of a service for participation. All participants were systematically examined by a physician for clinical systems of malaria and IP. Only asymptomatic participants were included in the study.

**Blood Collection and On-site Testing for Malaria**

Venous peripheral blood (about 4mL) was collected by venipuncture using a butterfly needle (G22) and a 5mL labeled EDTA tube from all 320 participants. Hemoglobin (Hb) was measured using the HemoCue (AB Leo Diagnostics, Helsingborg, Sweden). On site, after collecting the venous blood from the participants, a drop from the same collected blood was placed on a CareStart^TM^ Malaria pLDH/HRP-2 Combo Test (Access Bio Inc. USA) to detect histidine-rich protein-2 (HRP-2) specific to *Plasmodium falciparum* and Plasmodium lactate dehydrogenase (pLDH) pan-specific to *Plasmodium* spp (*falciparum, vivax, malariae, ovale*). Results were read according to manufacturer instructions and recorded after 5minutes.

**Laboratory detection, quantification and speciation of malaria parasites.**

Ten microliters of whole blood were used to prepare thick and thin smears for malaria parasite identification, speciation and quantification. The slides were air-dried overnight, and the thin blood smears were fixed in absolute (100%) methanol. Both thick and thin smears were stained using 10% Giemsa solution, washed with water and air-dried. Slides were then microscopically examined (thin and thick smear) for the presence of malaria parasites by two experienced microscopists. The parasite density was determined by counting the number of parasites against 200 leucocytes. The counts were expressed as the number of parasites per micro-liter of blood (Pf/µl), assuming an average leukocyte count of 8,000 cells/µl of blood (23). When the difference in parasitemia between the two readers was greater than 5 Pf/µl of blood, a third reader re-examined the slide and the mean of the two closest values were considered. Also, a differential count for eosinophil, lymphocytes, monocytes, neutrophils was obtained alongside parasitemia and different malaria species (by microscopy).

**Antibody analysis**

Plasma samples were tested for antibodies against the merozoite antigens MSP-1_42_, MSP-2, MSP-3 and EBA-175 using a multi-analyte platform (MAP) assay with antigen-coupled magnetic beads with different spectral addresses. Details of this assay used has been described previously (24) (25). In brief, plasma samples were diluted 1:100 with PBS, 50ul of plasma was incubated with 50ul antigen-coupled microspheres (2000 microspheres/test) for 60 minutes in the dark, washed with PBS, and then incubated at 500rpm for 60minutes at 25 °C on a rotating shaker and using a magnet plate separator. Then, 100 µl of secondary Ab (R-phycoerythrin-conjugated, Affini Pure F(ab′)_2_ fragment, Goat anti-human IgG Fc fragment specific, Jackson Immuno-research, West Grove, PA, USA, Cat no. 109-116-170) diluted to 2 µg/ml in PBS-1 % BSA was added to each well and incubated as above in the dark for 1 h. The mixture is then washed and a minimum of 100 beads were read in a MAGPIX® reader. A minimum bead count of 100 per spectral address recorded as Median Fluorescence Intensity (MFI).

Controls included on each plate were: PBS to determine background fluorescence, the negative control (NC) consisted of pooled plasma from four malaria-naïve US individuals, and the positive control (PC) was pooled plasma from Cameroonians with high antibody levels to *Plasmodium* falciparum. Results were exported to Excel for analysis. The cut-off for positivity was calculated as mean of MFI +3 standard deviation of the negative control as shown in the results sections.

**Stool sample collection and analysis**

Sterile labelled stool collection vials were given to the parents along with instructions for proper stool collection. All samples were analyzed within 7 hours of collection to avoid missing hookworm eggs and minimize chances of under reporting. Approximately, 4 mg of feces was suspended in 5ml PBS and a drop examined by wet mount. The Kato Katz technique was used for morphological identification of helminths eggs, e.g., *Ascaris. lumbricoides, Trichuris trichiura, or* larval stage of *Strongyloides stercoralis* (26) while the modified Ritchie’s concentration stool technique was used to identify all protozoans and cestodes (27). The smears were read at objective 10X for eggs and larvae and objective 40X for cysts and vegetative forms of protozoan. The number of eggs per gram of stool were estimated for the parasites listed.

Helminth eggs and protozoans were counted in about 4mg of stool and counts were extrapolated as the number of eggs per gram of stool. All stool slides were read by 2 technicians and in 2 different laboratories under supervision of a microbiologist and parasitologists.

**Data Analysis**

Data were analyzed using Microsoft Excel 2013, and GraphPad^®^ prism 8. Standard summary statistics were used to describe the study population and results are presented as proportions. Fischer’s exact test was used to compare antibody levels between the malaria-negative, IP-positive (MAL-, IP-+ ) and malaria-positive, IP-negative (MAL+,IP-) groups, because of the small sample sizes of the groups. The one-way-ANOVA test was used to compare all 4 groups. An unpaired t test was used to compare the means of the MAL-, IP- vs. MAL+, IP- groups. Kruskal-Wallis test was used to compare antibodies levels among the groups. An individual was considered to have a co-infection if at least one IP species and *P. falciparum* were present. Anemia was considered when Hb values were < 11.5 g/dL and classified according to WHO (28,29).

**Results**

The Study Population

A total of 320 children were enrolled (Table 1). Among the children, 75.6% were slide-positive for malaria (MAL+), with 58.8% having malaria without intestinal parasites (MAL+, IP-) and 16.9% being coinfected with malaria and intestinal parasites (MAL+, IP+). All subjects who tested positive for malaria using the rapid diagnostic field test were confirmed positive by microscopy. Among children who were infected with malaria, 71.3% were infected with only *P. falciparum* and 5% had *P. falciparum* and *P. malariae*. Interestingly, only 2.2% of the children had IP without malaria and 22.2% were negative for both malaria and IP.

The mean age of the children changed with infection status among the 4 groups (p = 0.0001) with the lowest age found in uninfected children (6.4 years) and highest in children with co-infections (9.3 years) (Table 1). Malaria infections were found in all age groups; whereas, none of the children under age 4 years had intestinal parasites. Mean haemoglobin levels were lower in children infected with malaria, but the difference was of marginal significance (p = 0.08; MAL-,IP- vs MAL+, IP-). The prevalence of anaemia was higher in children who were infected with malaria (MAL+,IP-)(p=0.034), but not those with co-infections (p >0.999) compared to children who were parasite-negative (MAL-,IP-).

| Table 1: Description of 320 children infected with malaria and intestinal parasites (IP) | | | | | |
| --- | --- | --- | --- | --- | --- |
|  | MAL-,  IP- | MAL+  IP- | MAL-  IP+ | Co-infections  (Mal+, IP+) | Total |
| Number (%) of children | 69  (21.6%) | 190  (59.4%) | 7  (2.2%) | 54  (16.9%) | 320 |
| Mean years of age* (range) | 6.4  (1-14) | 7.9  (1-15) | 8.6  (4-12) | 9.3  (4-15) |  |
| Parasitaemia: (median # infected erythrocytes/µl  (range) | 0 | 420/µl  (40-96,000) | 0 | 900/µl  (40 –30,970) |  |
| Measures of anaemia |  |  |  |  |  |
| Hb (g/dL) (mean ±SD) | 12.1 ±1.6 | 11.6 ± 2.2** | 12.2 ± 1.4 | 12.4±1.8 |  |
| Prevalence of anaemia |  |  |  |  |  |
| # (%) of children with Hb <11.5 g/dL | 21  (30.4%) | 87  (45.8%)*** | 2  (28.6%) | 17  (31.5) | 131 |
| *p = 0.0001; comparison among the 4 groups (ordinary one-way ANOVA)  **p = 0.087, comparison between MAL-,IP- vs. MAL+,IP- (unpaired t test).  ***p= 0.032, comparison between MAL-,IP- vs. MAL+,IP- (Fisher’s exact test) | | | | | |

Prevalence of Intestinal Parasites

Overall, 19.1% (61/320) of the children were positive for intestinal parasites, 16.9% of whom were also infected with malaria and 2.2% were IP+ but MAL- (Table 2). The major of helminth parasites detected were *Ascaris lumbrioides* (2.8%) and single cases of *Trichura sp*. and *Strongyloides sp.* Among the 320 children, 14.7% had detectable protozoan infections, including 7.8% infected with *Giardia lamblia*, 5.9% with *Entamoeba histolytica complex*, and 0.9% with *Isospora sp*. Very few children had intestinal cestodes (Table 2).

| Table 2: Prevalence of Intestinal Parasites (IP+) in  the 320 Children, Ages 1 to 15 years | | | |
| --- | --- | --- | --- |
|  | Number of Children | | |
|  | MAL-, IP+ | MAL+, IP+ | Total IP+  (% positive) |
| Intestinal Parasites |  | | |
| Helminths |  |  | 11 (3.4%) |
| *Ascaris lumbricoides* | 2 | 7 | 9 (2.8%) |
| Others* | 0 | 2 | 2 (0.87%) |
| Protozoans |  |  | 47 (14.7%) |
| *Giardia lamblia* | 3 | 22 | 25 (7.8%) |
| *Entamoeba histolytica complex* | 1 | 18 | 19 (5.9%) |
| Other** | 1 | 3 | 4 (0.9%) |
| Cestodes |  |  | 2 (0.63%) |
| *Hymenolepis nana* | 0 | 2 | 2 (0.63%) |
| Total IP | 7  (2.2%) | 54  (16.9%) | 61  (19.1%) |
| Others*: 1 *Trichura sp*. and 1 *Strongyloides sp.*  Others**: 3 *Isospora sp*. | | | |

Influence of Age on Malaria, Intestinal Parasites, Anaemia and Moderate Eosinophilia

As expected, children aged 1 through 2 years did not have soil-transmitted IP and had normal eosinophil levels; whereas, 63% of 1-2-year old were infected with malaria and had the highest prevalence of anemia (Table 3). In contrast, in children 9-15 years of age ~80% were slide-positive for malarial parasites, 24%-29% had intestinal parasites, and 10-38% had moderate eosinophilia. Thus, as children living in these villages increased with age, they developed partial immunity to malaria and anemia declined; whereas, the prevalence of IP and eosinophilia increased.

| Table 3: Influence of Age on Malaria, Intestinal Parasites, Anaemia and Percentage of Peripheral Eosinophils | | | | | |
| --- | --- | --- | --- | --- | --- |
| Age (years) | N = | % Mal+ | % IP+ | * % with anemia | **% with eosinophilia |
| 1 - 2 | 27 | 63.0 | 0 | 55.6 | 0 |
| 3 - 4 | 47 | 61.7 | 6.4 | 48.9 | 4.3 |
| 5-6 | 40 | 62.5 | 15.0 | 40.0 | 7.5 |
| 7-8 | 63 | 88.9 | 28.6 | 36.5 | 9.5 |
| 9-10 | 55 | 83.6 | 21.8 | 38.2 | 20.0 |
| 11-12 | 54 | 79.6 | 25.9 | 38.9 | 22.2 |
| 13-15 | 34 | 82.4 | 23.5 | 26.5 | 38.3 |
| *Anemia: Children with Hemoglobin less than 11.5 g/dL. **Moderate eosinophilia: ≥1,500 eosinophils/mm^3^ or ≥18.7% peripheral eosinophils | | | | | |

Antibody Levels to Malaria Merozoite Antigens

With repeated exposure to malaria, Ab prevalence and levels increased with age to the four merozoite antigens (Fig. 1). Among 1- to 2-year-olds, only 25% of the infants had Ab to EBA-175 and MSP3, 30% had Ab to MSP2, but 80% had Ab to MSP1 (Figure 1). However, by age 13-15 years, 60% had acquired Ab to MSP3 and >80% had Ab EBA-175, MSP2 and MSP3 (Fig. 1A). Among Ab-positive participants, Ab levels also increased with age (Fig. 1B-E). Although different amounts of Ab were ultimately obtained to the different antigens, the overall trend was for an increase in median Ab with age. Thus, it was important to take age into consideration when making comparison between children infected with malaria (MAL+,IP-), co-infected with malaria and IP (MAL+,IP+) and those who were not infected (MAL-,IP-) at the time the study was conducted.

Figure 1: Prevalence and amount of Ab in different age groups. A) Prevalence of Ab to the 4 merozoite antigens. The number of participants in each age group is provided in Table 3. Fig1B – E shows Ab levels (MFI) for children who were Ab-positive for each age group. Horizontal bars represent median Ab levels. Kruskal-Wallis test (nonparametric comparison among groups) values were for MSP1 (p=0.067); MSP2 (p<0.001); MSP3 (p=0.086) and EBA (p=0.053). MFI = Median fluorescence intensity; MSP = merozoite surface proteins; EBA= erythrocytes binding antigen

Comparison of Ab Levels in Participants with and without Malaria and IP

Since children below 3 years of age were not infected with IP, they were not included in the comparative studies described below. Given that Ab prevalence and levels increased with age, the study population was divided into 2 groups: children aged 3-10 years, a time period when children were becoming infected with IP (Table 3) and those 11-15 years, mainly children who had been infected repeatedly with malaria and may had lived with IP for a period of time. As predicted, Ab levels were slightly higher in MAL+ children due to current boosting compared to MAL-, but the differences were not significant (all p values >0.05) (Figure 2).

A comparison between Ab levels in children infected with malaria and co-infected with IP was conducted. Children with helminths and cestodes were not included in the analysis because the sample sizes were too small. Ab levels were compared between children aged 3-10 years infected with malaria (n=112) and co-infected with intestinal amoeba (n= 25 children), including *G. lamblia* (n= 15) and *E. histolytica* (n = 10 children) (Fig. 2). Antibody levels did not differ between malaria-infected children with or without intestinal amoeba for MSP1, MSP2 and MSP3; however, there were significantly higher Ab levels to EBA-175 in children co-infected with malaria and intestinal amoeba (p = 0.018) (Fig. 2D). The higher Ab levels were due to *E. histolytica* infections (p=0.0026), and not *G. lamblia* (p=0.3844). No differences were found between children aged 11 to 15 years for any of the antigens between children with malaria (single infection) and co-infected with any of the IP.

[FIGURE 2]

Fig 2. Antibody levels in children ages 3 to 10 for all antibody-positive individuals Distribution of Ab levels in MFI among malaria negative (MAL-) and malaria-positive (MAL+) and those co-infected with malaria plus amoeba (n=25); malaria plus *G. lamblia* (n=15) and malaria plus *E. histolytica* (n=10). The number of datapoints varied because not all participants had Ab to all antigens. Horizontal lines represent medians for the group. MFI = median florescence intensity; MSP = merozoite surface proteins, EBA = erythrocytes binding antigen (EBA)

**Discussion**

Malaria and polyparasitism (cestodes, protozoans, trematodes) are still common conditions throughout Africa(30,31). In the 1-15-year-old children living in the two rural Cameroonian villages surveyed, the prevalence of slide-positive malaria was 75.6% and 19.1% had intestinal parasites, with 16.9% co-infections (Table 1-2). This prevalence is similar to those found in other highly endemic regions of the country(32). This high transmission is related to geo-ecological and climatic conditions at the time of the study which was the transition from the dry to wet season, a period that favors vector breeding and distribution (33).

From Table 3 above, the prevalence of malaria ranged from 61% to 90% in different age groups implying that children in these villages were repeatedly exposed to malaria throughout their lives. Prior studies have established that repeated exposure induces immunity to malaria, with development of anti-disease immunity followed by anti-parasite immunity (34–37). As a result, the highest prevalence of 56% anemia was found in young children (3,38,39) with a decline to 27% in 13 to 15-year-olds (Table 3). On the other hand, Infections with IP only occurred later in life from 3 years onward with a mean infection age of 8.1 years. Increase in Intestinal parasites was associated with an age-related increase in eosinophil counts (40,41), a known innate immune response to helminthic and other soil-transmitted organisms (Table 3). In this study, only 11/320 (3.4%) children were infected with helminths. Although some epidemiological studies have demonstrated an increased risk of infection by P. falciparum in individuals co-infected with helminths, other results are conflicting (42,43). This could be explained in part by the fact that mass community de-worming is done biannually following the national infectious disease guide-line for IP control program. The most prevalent intestinal parasites were the protozoans, *Giardia intestinalis* and *Entamoeba histolytica* (48). These protozoa are commonly found in damp soil and contaminated water with a prevalence of 2-20% in Cameroon(48–51). These results suggest children acquire their intestinal infections after learning to walk and interact with the environment. Thus, children in the study population were exposed to malaria early in life and began developing anti-malaria immunity prior to exposure to intestinal parasites.”

Generally, both Ab prevalence and Ab levels increased with age in 1 to 15-year-olds living in this high transmission area (Fig. 1). Often, the presence of Ab is used as markers of infection. This study compared antibody levels with age in four main groups of children, MAL+IP+, MAL-IP-, MAL-IP + and MAL+,IP- children to four (MSP1, MSP2 MSP3, EBA17) malaria antigens (52–54). Since over 80% of 1-2-year-olds had Ab to MSP1, humoral immunity began to develop early in life and continued to mature as children developed into adolescents (Fig. 1). Often individuals who are MAL+ have higher Ab levels than MAL- individuals due to boosting of the Ab response(34,36,37). In the current study, Ab levels did not differ significantly between MAL+ than MAL- individuals, neither those who were 3-10 years nor 11-15 years-old. This result, however, was not surprising, since 75% of the children were slide-positive for malaria (Table 1). Therefore, it is likely that children who were slide-negative had either been recently infected or had submicroscopic infections. In essence, the immune response in individuals who are repeatedly infection would be similar to that produced during chronic infections.

Prior studies have demonstrated that malaria-helminths co-infections can down regulate malaria and orient the immune response via the Th2 response hence, making patients less sick (21,34,55,56) whereas, others have demonstrated on the contrary that IP and malaria co-infections increase malaria disease (14,54). Unfortunately, the current study could not resolve the controversy because very few children had helminthic infections, due to frequent treatment with albendazole. However, co-infections with malaria and amoeba were relatively common. Ab levels to MSP1, MSP2 and MSP3 were similar in children infected with *P. falciparum* alone and those with amoeba (Fig. 2); however, significantly higher Ab levels to EBA-175 were found in children co-infected with malaria and intestinal amoeba (p = 0.018). The higher Ab levels were due to *E. histolytica* infections (p = 0.0026), and not *G. lamblia* (p = 0.384). This result was unexpected. *E. histolytica* is a gut amoeba that cause both intestinal and extraintestinal infections such as amebic colitis (dysentery) and liver or brain abscess. The protozoa cause a marked down-regulation of macrophage functions rendering the cells incapable of antigen presentation and unresponsive to cytokine stimulation (57).

In addition to a possible immunological interaction, there are at least 2 other explanations as to why *E. histolytica* infections might be associated with higher Ab levels to EBA-175. First, children living in moist or wet environments where mosquitoes breed and *E. histo*lytica are more abundant would have a high risk of acquiring both infections, that would result in frequent boosting of the Ab response. Secondly, since malaria and *E. histolytica* are both amoebae, they might share common antigens, for example, EBA-175 could share homology with an *E. histolytica* antigen. To investigate this possibility, a blast search of the NCIB gene bank was conducted for EBA-175 and the *E. histolytica* genome. However, this search showed no significant similarity between both gene sequences.

Finally, an alternative explanation could be that this result is a spurious observation by chance. Clearly the association between malaria and *E. histolytica* merits further study.

Altogether, a keen observation needs to be repeated with a larger sample size as *E. histolytica* boosting of Ab to EBA-175 – co-infection might not only be limited to EBA-175, but other antigens as well. Children in these villages began to acquire an Ab response to the 4 merozoite antigens early in life, prior to infection with IP. There was no evidence that infection with IP influenced Ab levels or negatively-altered the already established Ab response to the 4 merozoite antigens.

**CONCLUSION**

The prevalence of malaria was high in children 1-2 years old; whereas, intestinal parasite infections occurred in children over 3 years old. Thus, immunity to *P. falciparum* began prior to infection with soil-transmitted parasites. No differences were found in antibody prevalence or levels in malaria-infected and co-infected children, except antibody levels to EBA175 were significantly higher in children co-infected with malaria and *E. histolytica*. This is the first report of an interaction between malaria and *E. histolytica* and antibodies to EBA-175 and merits further evaluation.

**Declarations**

**Ethical consideration**

Ethical clearance used for the study was obtained from the Cameroon National Ethics Committee (IRB approval: N^o^2016/12/845/CE/CNERSH/SP). Administrative authorizations were obtained from authorities of the Ngali II and Mfou health districts.

Informed consents were obtained from parents of all participants. A clinical examination was performed for all eligible participants by a medical doctor.

All participants positive for any *Plasmodium s*pp by RDT at the time of blood collection and those who were found to have PI by stool analysis were treated for free following the protocol recommended by the Cameroonian Ministry of Health. All children with mild anemia were given an iron supplement free of charge.

**Authors’ contributions**

GFLR supervised the study. JDB and WM co-supervised the study. NCM and ELG conceived and designed the work. NCM, EFL, DJC, AEW, MBN carried out experiments. Data was collected and analyzed by NCM. The first draft of this manuscript was written by NCM, critically read and edited by DWT, YML. DWT and GFLR reviewed the final draft.

**Acknowledgements**

We heartily thank all the participants and their parents who let them participate in the study in Ngali II and Mfou. We equally express our gratitude to the community health workers of these areas for their assistance during sample collection.

Anna Babakhanyan, of Hawaii university provided the recombinant proteins (beads) used for the assays and the entire staff of the Biotechnology Centre of University of Yaoundé I, Cameroon for their tremendous work in the field during sample collection and sample processing in the lab.

**Funding Statement:**

**The beats used for the experiments were a gift from A. Babakhanyan (University of Hawaii) and coupled by ELG. The MAGPIX used for the data analysis was MAGPX I3038703, Luminex corporation 12212 technology Blvd Austin. Texas 78727. GFLR provided the funds for the field work and analysis.**

**Competing Interest:**

Authors declare no competing interests.

**Data Availability:**

The authors approve of the availability of all data underlying the findings and without restriction upon reasonable request from the corresponding authors. All-important data are within the paper.

**Consent for publication** : All authors give their consent for publication of this manuscript.

**References:**

1.	World Malaria Report 2016 - 9789241511711-eng.pdf [Internet]. [cited 2016 Dec 17]. Available from: http://apps.who.int/iris/bitstream/10665/252038/1/9789241511711-eng.pdf

2.	WHO | World Malaria Day 2016: End malaria for good [Internet]. WHO. [cited 2016 Nov 26]. Available from: http://www.who.int/campaigns/malaria-day/2016/event/en/

3.	Chen I, Clarke SE, Gosling R, Hamainza B, Killeen G, Magill A, et al. “Asymptomatic” Malaria: A Chronic and Debilitating Infection That Should Be Treated. PLOS Med. 2016 Jan 19;13(1):e1001942.

4.	Pampiglione S, Visconti S, Pezzino G. [Human intestinal parasites in Subsaharan Africa. II. Sao Tomé and Principe]. Parassitologia. 1987 Apr;29(1):15–25.

5.	Fletcher SM, Stark D, Ellis J. Prevalence of gastrointestinal pathogens in Sub-Saharan Africa: systematic review and meta-analysis. J Public Health Afr [Internet]. 2011 Sep 5 [cited 2018 Sep 9];2(2). Available from: https://www.ncbi.nlm.nih.gov/pmc/articles/PMC5345503/

6.	Fusi-Ngwa C, Besong E, Pone JW, Mbida M. A Cross-Sectional Study of Intestinal Parasitic Infections in Children in Ghettoed, Diverse and Affluent Communities in Dschang, West Region, Cameroon. Open Access Libr J. 2014 Dec 1;01:1.

7.	Mwangi TW, Bethony JM, Brooker S. Malaria and helminth interactions in humans: an epidemiological viewpoint. Ann Trop Med Parasitol. 2006 Oct;100(7):551–70.

8.	Zeukeng F, Tchinda VHM, Bigoga JD, Seumen CHT, Ndzi ES, Abonweh G, et al. Co-infections of Malaria and Geohelminthiasis in Two Rural Communities of Nkassomo and Vian in the Mfou Health District, Cameroon. PLoS Negl Trop Dis [Internet]. 2014 Oct 16 [cited 2016 Dec 5];8(10). Available from: http://www.ncbi.nlm.nih.gov/pmc/articles/PMC4199518/

9.	Njunda AL, Fon SG, Assob JCN, Nsagha DS, Kwenti TDB, Kwenti TE. Coinfection with malaria and intestinal parasites, and its association with anaemia in children in Cameroon. Infect Dis Poverty [Internet]. 2015 Oct 6 [cited 2016 Dec 8];4. Available from: http://www.ncbi.nlm.nih.gov/pmc/articles/PMC4595138/

10.	Hopkin J. Immune and genetic aspects of asthma, allergy and parasitic worm infections: evolutionary links. Parasite Immunol. 2009;31(5):267–73.

11.	Nacher M, Gay F, Singhasivanon P, Krudsood S, Treeprasertsuk S, Mazier D, et al. Ascaris lumbricoides infection is associated with protection from cerebral malaria. Parasite Immunol. 2000 Mar 1;22(3):107–13.

12.	Brutus L, Watier L, Hanitrasoamampionona V, Razanatsoarilala H, Cot M. Confirmation of the protective effect of Ascaris lumbricoides on Plasmodium falciparum infection: results of a randomized trial in Madagascar. Am J Trop Med Hyg. 2007 Dec;77(6):1091–5.

13.	Author(s): M. Nacher, P. Singhasivanon, S. Yimsamran, W. Manibunyong, N. Thanyavanich, P., Wuthisen, and S. Looareesuwan, Author(s): M. Nacher, P. Singhasivanon, S. Yimsamran, W. Manibunyong, N. Thanyavanich, P. INTESTINAL HELMINTH INFECTIONS ARE ASSOCIATED WITH INCREASED INCIDENCE OF PLASMODIUM FALCIPARUM MALARIA IN THAILAND [Internet]. [cited 2017 May 1]. Available from: http://sci-hub.cc/http://dx.doi.org/10.1645/0022-3395(2002)088[0055:IHIAAW]2.0.CO;2

14.	Sokhna C, Le Hesran J-Y, Mbaye PA, Akiana J, Camara P, Diop M, et al. Increase of malaria attacks among children presenting concomitant infection by Schistosoma mansoni in Senegal. Malar J. 2004;3:43.

15.	Lo AC, Faye B, Gyan BA, Amoah LE. Plasmodium and intestinal parasite perturbations of the infected host’s inflammatory responses: a systematic review. Parasit Vectors [Internet]. 2018 Jul 3 [cited 2019 Sep 16];11. Available from: https://www.ncbi.nlm.nih.gov/pmc/articles/PMC6031113/

16.	Njua-Yafi C, Nkuo-Akenji T, Anchang-Kimbi J, Apinjoh T, Mugri R, Chi H, et al. The Effect of Helminth Co-Infection on Malaria-Specific Immunoglobulin G Responses. BMJ Glob Health. 2017 Feb 1;2(Suppl 2):A66–A66.

17.	Nacher M. Malaria vaccine trials in a wormy world. Trends Parasitol. 2001 Dec 1;17(12):563–5.

18.	Maizels RM, Pearce EJ, Artis D, Yazdanbakhsh M, Wynn TA. Regulation of pathogenesis and immunity in helminth infections. J Exp Med. 2009 Sep 28;206(10):2059–66.

19.	Ateba-Ngoa U, Jones S, Zinsou JF, Honkpehedji J, Adegnika AA, Agobe J-CD, et al. Associations between Helminth Infections, Plasmodium falciparum Parasite Carriage and Antibody Responses to Sexual and Asexual Stage Malarial Antigens. Am J Trop Med Hyg. 2016 Aug 3;95(2):394–400.

20.	Hartgers FC, Yazdanbakhsh M. Co-infection of helminths and malaria: modulation of the immune responses to malaria. Parasite Immunol. 2006 Oct 1;28(10):497–506.

21.	Sanchez-Arcila JC, Perce-da-Silva D de S, Vasconcelos MPA, Rodrigues-da-Silva RN, Pereira VA, et al. Intestinal Parasites Coinfection Does Not Alter Plasma Cytokines Profile Elicited in Acute Malaria in Subjects from Endemic Area of Brazil. Mediators Inflamm. 2014 Sep 16;2014:e857245.

22.	Kuitcha D, Fouepe A, Ndjama J, Takem G, Awah M, Kamgang V. Chemical and isotopic signal of precipitation in Yaounde-Cameroon. Arch Appl Sci Res. 2012 Oct 15;4:2591–7.

23.	District Laboratory Practice in Tropical Countries Part 1, Second Edition - monica-cheesbrough-district-laboratory-practice-in-tropical-countries-part-1.pdf [Internet]. [cited 2017 Nov 14]. Available from: https://medicallabtechno.weebly.com/uploads/7/5/1/5/7515789/monica-cheesbrough-district-laboratory-practice-in-tropical-countries-part-1.pdf

24.	Fodjo BAY, Atemnkeng N, Esemu L, Yuosembom EK, Quakyi IA, Tchinda VHM, et al. Antibody responses to the full-length VAR2CSA and its DBL domains in Cameroonian children and teenagers. Malar J [Internet]. 2016 [cited 2020 Feb 8];15. Available from: https://www.ncbi.nlm.nih.gov/pmc/articles/PMC5097422/

25.	Babakhanyan A, Fang R, Wey A, Salanti A, Sama G, Efundem C, et al. Comparison of the specificity of antibodies to VAR2CSA in Cameroonian multigravidae with and without placental malaria: a retrospective case–control study. Malar J. 2015 Dec 1;14(1):480.

26.	Lindholz CG, Favero V, Verissimo C de M, Candido RRF, Souza RP de, Santos RR dos, et al. Study of diagnostic accuracy of Helmintex, Kato-Katz, and POC-CCA methods for diagnosing intestinal schistosomiasis in Candeal, a low intensity transmission area in northeastern Brazil. PLoS Negl Trop Dis. 2018 Mar 8;12(3):e0006274.

27.	Yanet F-S, Fidel Angel N-F, Guillermo N, Sergio S-P. Comparison of parasitological techniques for the diagnosis of intestinal parasitic infections in patients with presumptive malabsorption. J Parasit Dis Off Organ Indian Soc Parasitol. 2017 Sep;41(3):718–22.

28.	Haemoglobin_en.doc - haemoglobin WHO .pdf [Internet]. [cited 2017 Aug 21]. Available from: http://www.who.int/vmnis/indicators/haemoglobin.pdf

29.	Hemoglobin Concentration (Hb): Reference Range, Interpretation, Collection and Panels. 2016 Jun 1 [cited 2017 May 18]; Available from: http://emedicine.medscape.com/article/2085614-overview

30.	Degarege A, Veledar E, Degarege D, Erko B, Nacher M, Madhivanan P. Plasmodium falciparum and soil-transmitted helminth co-infections among children in sub-Saharan Africa: a systematic review and meta-analysis. Parasit Vectors [Internet]. 2016 Jun 15 [cited 2020 Feb 11];9. Available from: https://www.ncbi.nlm.nih.gov/pmc/articles/PMC4908807/

31.	Hürlimann E, Yapi RB, Houngbedji CA, Schmidlin T, Kouadio BA, Silué KD, et al. The epidemiology of polyparasitism and implications for morbidity in two rural communities of Côte d’Ivoire. Parasit Vectors. 2014 Feb 25;7:81.

32.	Akinbo FO, Omoregie R, Mordi R, Okaka CE. Prevalence of Malaria and Anemia Among Young Children in a Tertiary Hospital in Benin City, Edo State, Nigeria. Fooyin J Health Sci. 2009 Nov 1;1(2):81–4.

33.	Achonduh-Atijegbe OA, Mfuh KO, Mbange AHE, Chedjou JP, Taylor DW, Nerurkar VR, et al. Prevalence of malaria, typhoid, toxoplasmosis and rubella among febrile children in Cameroon. BMC Infect Dis. 2016 Nov 8;16(1):658.

34.	ARTAVANIS-TSAKONAS K, TONGREN JE, RILEY EM. The war between the malaria parasite and the immune system: immunity, immunoregulation and immunopathology. Clin Exp Immunol. 2003 Aug;133(2):145–52.

35.	Roetynck S, Baratin M, Vivier É, Ugolini S. Cellules natural killer et immunité innée contre le paludisme. médecine/sciences. 2006 Aug 1;22(8–9):739–44.

36.	Griffin JT, Hollingsworth TD, Reyburn H, Drakeley CJ, Riley EM, Ghani AC. Gradual acquisition of immunity to severe malaria with increasing exposure. Proc R Soc B Biol Sci [Internet]. 2015 Feb 22 [cited 2020 Feb 11];282(1801). Available from: https://www.ncbi.nlm.nih.gov/pmc/articles/PMC4309004/

37.	Bediako Y, Adams R, Reid AJ, Valletta JJ, Ndungu FM, Sodenkamp J, et al. Repeated clinical malaria episodes are associated with modification of the immune system in children. BMC Med [Internet]. 2019 Mar 13 [cited 2020 Feb 11];17. Available from: https://www.ncbi.nlm.nih.gov/pmc/articles/PMC6415347/

38.	Haldar K, Mohandas N. Malaria, erythrocytic infection, and anemia. Hematol Am Soc Hematol Educ Program. 2009;87–93.

39.	World Malaria Report 2016 (annexes) - WMR-2016-annexes.pdf [Internet]. [cited 2017 May 1]. Available from: http://www.who.int/malaria/publications/world-malaria-report-2016/WMR-2016-annexes.pdf

40.	Kovalszki A, Weller PF. Eosinophilia. Prim Care. 2016 Dec;43(4):607–17.

41.	O’Connell EM, Nutman TB. Eosinophilia in Infectious Diseases. Immunol Allergy Clin North Am. 2015 Aug;35(3):493–522.

42.	LYKE KE, DICKO A, DABO A, SANGARE L, KONE A, COULIBALY D, et al. ASSOCIATION OF SCHISTOSOMA HAEMATOBIUM INFECTION WITH PROTECTION AGAINST ACUTE PLASMODIUM FALCIPARUM MALARIA IN MALIAN CHILDREN. Am J Trop Med Hyg. 2005 Dec;73(6):1124–30.

43.	Melo GC, Reyes-Lecca RC, Vitor-Silva S, Monteiro WM, Martins M, Benzecry SG, et al. Concurrent Helminthic Infection Protects Schoolchildren with Plasmodium vivax from Anemia. PLOS ONE. 2010 Jun 21;5(6):e11206.

44.	Giardia: Overview and Update [Internet]. [cited 2017 May 16]. Available from: https://oup.silverchair-cdn.com/oup/backfile/Content_public/Journal/cid/25/3/10.1086/513745/2/25-3-545.pdf?

45.	Giardiasis: Background, Pathophysiology, Etiology. 2016 Dec 2 [cited 2017 May 16]; Available from: http://emedicine.medscape.com/article/176718-overview

46.	Entamoeba histolytica - Overview [Internet]. Encyclopedia of Life. [cited 2017 May 16]. Available from: http://eol.org/pages/491174/overview

47.	Amebiasis: Background, Pathophysiology, Etiology. 2017 Jan 6 [cited 2017 May 16]; Available from: http://emedicine.medscape.com/article/212029-overview

48.	Richardson DJ, Callahan KD, Dondji B, Tsekeng P, Richardson KE. Prevalence of Waterborne Protozoan Parasites in Two Rural Villages in the West Province of Cameroon. Comp Parasitol. 2011 Jan;78(1):180–4.

49.	Mbuh JV, Ntonifor NH, Ojong J. The epidemiology of soil-transmitted helminth and protozoan infections in south-west Cameroon. J Helminthol. 2012 Mar;86(1):30–7.

50.	Mbuh JV, Ntonifor HN, Ojong JT. The incidence, intensity and host morbidity of human parasitic protozoan infections in gastrointestinal disorder outpatients in Buea Sub Division, Cameroon. J Infect Dev Ctries. 2009 Dec 28;4(1):38–43.

51.	Kuete T, Yemeli FLS, Mvoa EE, Nkoa T, Somo RM, Ekobo AS. Prevalence and Risk Factors of Intestinal Helminth and Protozoa Infections in an Urban Setting of Cameroon: the Case of Douala. Am J Epidemiol Infect Dis Am J Epidemiol Infect Dis. 2015 Jun 16;3(2):36–44.

52.	Diallo TO, Remoue F, Gaayeb L, Schacht A-M, Charrier N, Clerck DD, et al. Schistosomiasis Coinfection in Children Influences Acquired Immune Response against Plasmodium falciparum Malaria Antigens. PLOS ONE. 2010 Sep 15;5(9):e12764.

53.	Courtin D, Djilali-Saïah A, Milet J, Soulard V, Gaye O, Migot-Nabias F, et al. Schistosoma haematobium infection affects Plasmodium falciparum-specific IgG responses associated with protection against malaria. Parasite Immunol. 2011 Feb 1;33(2):124–31.

54.	Hartgers FC, Obeng BB, Kruize YCM, Dijkhuis A, McCall M, Sauerwein RW, et al. Responses to Malarial Antigens Are Altered in Helminth‐Infected Children. J Infect Dis. 199:1528–35.

55.	Su Z, Segura M, Stevenson MM. Reduced Protective Efficacy of a Blood-Stage Malaria Vaccine by Concurrent Nematode Infection. Infect Immun. 2006 Apr 1;74(4):2138–44.

56.	Lemaitre M, Watier L, Briand V, Garcia A, Hesran JYL, Cot M. Coinfection with Plasmodium falciparum and Schistosoma haematobium: Additional Evidence of the Protective Effect of Schistosomiasis on Malaria in Senegalese Children. Am J Trop Med Hyg. 2014 Feb 5;90(2):329–34.

57.	Entamoeba histolytica: Host Defense and Immune Responses [Internet]. http://www.eurekaselect.com. [cited 2020 Feb 11]. Available from: http://www.eurekaselect.com/54531/chapter
