# Supplementary material for: The immunoglobulin G antibody response to malaria merozoite antigens in asymptomatic children co-infected with malaria and intestinal parasites
Source: PLoS One. 2020 Nov 10;15(11):e0242012. doi: 10.1371/journal.pone.0242012 (PMC7654760; doi:10.1371/journal.pone.0242012)
Supplement: Attachment — Submitted filename: Response to Reviewers.docx [file pone.0242012.s004.docx]

**Reviewer #1**: Dr. Mbe-cho and colleagues sought to determine the prevalence of co-infection of malaria and intestinal parasites and its association with antibody levels to malaria merozoite antigens. The authors report that there was no difference in antibody prevalence or levels in malaria-infected and co-infected children, except antibody levels to EBA-175 were significantly higher in children co-infected with malaria and E. histolytica. Overall, the study is well-designed but these results do not significantly alter or impact our understanding of the association of malaria and helminths on antibody to malaria merozoite antigens.

1. The limitation of the study is that the parasite testing in children was not followed by sensitive diagnostic techniques like PCR, and light infections may have been missed which may have resulted in misclassification of the groups. Light infections may boost the antibody responses while children remain asymptomatic.

*Reply: We understand the concern. When the study was conducted (2017) in the rural villages, the prevalence of slide-positive malaria was 75.6%. In a prior study conducted in the village (Leke et al 2010), an equivalent prevalence was found of P. falciparum (50-85%) in children aged 5-15 years over a 5-year period. The estimated entomological inoculation rate (EIR) was 0.7 infectious bites/person/ nightly thought out the year (~257 IB/P/Y). Based on the more recent malaria prevalence, it appears that the current EIR is similar. Thus, children were most likely being bitten approximately every-other night by an infectious mosquito, since bednets were not routinely used. With this high level of transmission, most of the slide-negative children would be PCR-positive for malaria, i.e., have enough immunity to reduce malaria to submicroscopic levels. Unfortunately, in very high transmission areas like the one reported herein, everyone will have some circulating P. falciparum parasites. So, classifying subjects as slide-positive vs slide-negative may not reflect presence/absence of parasites, but provide information on the immune status of the person. In revising the MS, information from the study by Leke et al. was included as well as a discussion of submicroscopic infections in the revised Discussion.*

2. In this study, only 3.4% children were infected with helminths alone to get any meaningful data for antibody response to malaria in this group.

*Reply: We agree, the sample size of children with helminth infections is too small to provide meaningful information. Accordingly, Ab levels in children with helminth infections were not analyzed. To explain the low prevalence of helminths, information on the Ministry of Heatlth’s policy for biannual treatment of children for worms was provided.*

3. Very few children are positive for E. histolytica.

*Reply: True, the prevalence of Entamoeba in our study was only 5.9%, which is lower than that reported in studies in these areas of ~23% (T. E. Kwenti et al., 2016). In our study, the prevalence was lower, probably due to rigorous mass drug administration (MDA) programs implemented by the Ministry of Health and other regular or seasonal health campaigns*.

4. The data on the children's anthropomorphic measurements are not mentioned. Thus, there is not much point describing how they were collected.

*Reply: This section was removed from the Methods section.*

5. There is no data on hookworm infection in the results.

*Reply: The prevalence of hookworm infections was considered in this study during stool exams and, surprisingly, we did not find hookworms in the samples collected, most likely due to regular deworming and improved hygiene in the area. No invasive methods were used for diagnosis of adult worms. From a paper published by E. Kwenti et al. (2016) the prevalence of hookworm was 7% in south west region Cameroon.*

6. The number of eggs per gram of stool were estimated for the parasites listed. Did the authors look at the responses in children with high or low intensity of the parasites?
*Reply: In this study, after obtaining the prevalence of parasites and comparing with antibody response, no significant difference was observed between the malaria antibodies levels and parasites eggs counts.*

7. Table 2 is not necessary, it can be written as text.

*Reply: Thanks for the comment, but we think Table 2 summarizes the data more clearly and allows readers to easily compare results from different groups than presenting them in the text. Table 2 has been revised.*

8. Page 21, reference # 54, year of publication is missing.

*Reply: Year of publication has been included.*

Please check spelling and typographical errors scattered through the manuscript (page and lines are given from word document):
1. Page 2, line 3, change led to lead in the sentence.
*Reply: The word “led” has been changed to “lead”.*

2. Page 2, line 14, correct the spelling of Rietchi concentration method
*Reply: Spelling has been corrected to “Ritchie”*

3. Page 6, line 21: The bracket has to be closed here: (AB Leo Diagnostics, Helsingborg, Sweden.

*Reply: The bracket has been closed.*

4. Page 7, line 17 and 18: Correct 50ul to 50µl
*Reply: The change has been made.*

5. Page 9 and 10: In the text, the p value for anemia (MAL+,IP-) is p=0.034; p value for the same in Table 1 is p=0.032; it needs to be corrected.
*Reply: P value has been corrected to P=0.032 (correct value) in the text.*

6. Page 10: In Table 1, % sign is missing in column 5 for children with Hb.
*Reply: The % symbol has been included in table 1, column 5.*

7. Page 10, line 3: In the sentence, change major to majority.
*Reply: The word “major” has been changed to “majority”.*

8. Page 14, line 27: In the sentence, MSL- should be MAL-
*Reply: In Line 27 of page 14, MSL- has been changed to MAL-*

9. Page 17, line 15: change beats to beads
*Reply: The spelling of beads has been corrected.*

10. Re-write the following sentences, they are not very clear:

Page 4, line 8:
However, with most children getting infected with several episodes of infections in a short period, this renders them more prone to having clinical symptoms since the immune systems doesn’t fully recover.

*Reply: The sentence has been deleted because the information is not directly relevant to the study.*

Page 4, line 20:
Concomitant infections in humans have suggested that Ascaris lumbricoides infection may protect against cerebral malaria (11,12), while other studies, children infected by S. mansoni were more susceptible to P. falciparum infection and develop acute malaria episodes.

*Reply: The sentence has been revised to read: “Studies on concomitant infections in humans suggest that A. lumbricoides infection may protect against cerebral malaria (11,12), while other studies suggest that children infected by S. mansoni may be more susceptible to* P. falciparum *infections and develop acute malaria episodes (13,14).”*

Page 15, line 3:
In essence, the immune response in individuals who are repeatedly infection would be similar to that produce during chronic infections.

*Reply: To clarify the statement, the text has been revised to read: “Because of high transmission, the children are becoming infected almost daily and are either in the process of eliminating the new infection or reducing it to a submicroscopic level. Because of constant re-exposure, the resulting immune response will be similar to that produced by a chronic infection.*

**Reviewer #2:** The answer to the questions is divided into Major comments, Minor comments. Additionally, I wrote minor observations that, I hope, will help this manuscript to improve readability and consistency.

1. Is the manuscript technically sound, and do the data support the conclusions?
2. Has the statistical analysis been performed appropriately and rigorously?
3. Have the authors made all data underlying the findings in their manuscript fully available?
4. Is the manuscript presented in an intelligible fashion and written in standard English?

Major comments:

• Given that there were no differences in the IgG response between age groups, it would be interesting to join these data, evaluate all the coinfected individuals, and then split the data into Giardia, E. hystolitica.

*Reply: We are confused by this comment, because Fig 1 shows an increase in both Ab prevalence (Fig. 1A) and Ab levels (Fig 1 B-E) with age in Ab-positive children (Kruskal-Wallis test p values were p<0.001 MSP2 and p=0.05-0.086 (borderline) for the other antigens).*

*We believe combining all MAL+,IP+ children into single a group is unwise, since they were infected with a conglomerate of intestinal helminths, cestodes and protozoa (see Table 2). Combining children with such heterogenous infections is unlikely to provide meaningful information.*

• I strongly suggest dividing the age of individuals in 0-5, 5-10, 10-15 years-old to partially solve the "N" problem of the groups.

*Reply: Thanks for the comment. Initially, children were groups into 5-year categories as suggested by the Reviewer, i.e., 0-5, 5-10, 10-15 years old. However, when the data set showed that children aged 1 to 2 did not have intestinal parasites, the results were grouped into 2-year intervals, that allowed us to more closely define the increase in Ab prevalence (Fig. 1A) and Ab levels (Fig 1 -B,C,D,E) with age. The purpose of Fig 1 was to determine if age was a variable that needed to be taken into consideration during data analysis.*

• Because of the absence of molecular Diagnosis and considering that the authors mention the possibility of oh having low parasitemia infections in the MAL- group. It is important to include MAL- individuals in Figure 1.

*Reply: We are sorry if we didn’t make the point clear. ALL children who were Ab-positive are included in Fig 1, including those who are MAL+ and* ***MAL-****. Because malaria transmission is high in the area, all children in the study had been exposed to P. falciparum and many of the MAL- children were Ab-positive.*

• It is necessary to compare parasite data with similar regions in Cameroon. Please compare and cite:
• (Malaria and Helminth Co-Infection in Children Living in a Malaria Endemic Setting of Mount Cameroon and Predictors of Anemia from Theresa K Nkuo-Akenji et al. 2006)
• Malaria, Helminths, Coinfection and Anaemia in a Cohort of Children From Mutengene, South Western Cameroon from Clarisse Njua-Yafi et al. 2016.

*Reply: We thank the Reviewer for pointing out the omission of key references. Information from these studies have been included in the revised Discussion. The text now reads, “…..to those found in other highly [malaria] endemic regions of the country (32), and the prevalence of co-infections was 19.1%, which is similar to the prevalence of co-infections of 18 – 27% reported in other regions of Cameroon (9,44).* The references have been added to the reference section.

• Do the authors have information about malaria and intestinal parasites last treatments? On page 17, it was commented that Albendazole treatment was frequent in these children. Deworming information will help the readers to understand why the prevalence of intestinal parasites was low compared with other studies in Cameroon. Additionally, reinforce in the discussion section that collecting/reporting that information is valuable for coinfection studies.

*Reply: In response to the Reviewer’s suggestion, the following information has been added to the Methods section. “Currently, mass drug administration with albendazole is being performed twice a year by the Ministry of Health, that is usually conduced in schools and symptomatic cases are sent to the local clinic or hospital for follow up treatment.”*

• (Figure 1 B, C, D, E) use the same scale limits for all plots. This is also useful to understand differences in levels of antigenicity between proteins.

*Reply: We understand the comment, but we do not wish to change the Y-axis on Fig 1, since it is risky to make a direct comparison of Ab levels between antigens in serological assays. A number of variables, including parasite strain, the system to produce recombinant proteins, protein purity, the amount of antigen used, number of exposed epitopes, dilution of plasma, etc., influence the overall results. Even when Luminex beads are covalently-coupled with saturating amounts of antigen, it is questionable if direct comparison of MFI can be made between antigens. Although our assays have been optimized and equivalence amounts of antigen used during bead-coupling, comparisons among the antigens may not provide accurate information about immunogenicity. In Figs1 B, C, D, E, the Y-Axis was selected to show the best distribution of the MFI results.*

• (table 3) How could the authors explain increased eosinophilia with low levels of helminth infection? This mainly applies to the age group > 9 years-old.

*Reply: After age 2, children start becoming infected with helminths, resulting in an increase in eosinophil counts. During the biannual drug treatment campaign, helminthic infections are eliminated, but eosinophilia persists for a period of time. With increasing age, more children in the area become i) infected and ii) re-infected, resulting in an increase in prevalence of eosinophilia.*

• (Page 17) The authors argue, "First, children living in moist or wet environments where mosquitoes breed and E. histolytica are more abundant would have a high risk of acquiring both infections, that would result in frequent boosting of the Ab response." This explanation for intestinal parasite influence on antibody production alteration is not viable since Giardia's frequency is higher than E. histolytica in the studied population.

*Reply: The sentence has been deleted from the Discussion.*

• (Page 17) The affirmation "Secondly, since malaria and E. histolytica are both amoebae, they might share common antigens, for example, EBA-175 could share homology with an E. histolytica antigen." is false. Plasmodium falciparum is not an is a protozoan. This group belongs to Apicomplexa organisms. For that reason, the hypothesis about correlating Plasmodium and E. histolytic is wrong.

*Sorry, “amoebae” was a typo. Both Plasmodium falciparum and E. histolytica are protozoans. The Discussion has been revised to read “parasitic protozoa.”*

• How different are the two Villages Ngali II and Mfou in the central region of Cameroon? Does it exist a difference in humidity and soil moist, once the authors claimed that this variable could explain differences of Entamoeba histolytica?

*Reply: The two villages are very similar with no major differences in humidity or soil moisture. The estimated annual average rainfall measures 1600 mm^3^ with an annual average temperature of 23°C for Ngali II and for Mfou. According to the National Meteorology agency, the average humidity for the center regions is 83%. Ngali and Mfou are both in the center region of Cameroon about 60km apart. Note: as mentioned above, the words “humidity and soil moisture” have been deleted from the MS.*

Minor comments:
• What criteria were used to divide the population into seven groups according to age?

*Reply: The fact that Intestinal parasite (IP) infections was only observed in children >2 years, helped guide separation of the children into seven groups.*

• Please specify how anthropometric parameters were used in the study, once they were described but not used in the study. If this information was not used, please remove these sentences.

*Reply: The sentence has been removed.*

• Has the studied region presence of Schistosoma haematobium? If the authors have register if this parasite in the area, Did they examined urine samples to discard infections with this parasite?

*Reply: Detection of S. haematobium was not included in the study design because of low prevalence in the study area. A study conducted in this area (and other regions of Cameroon) by Louis-Albert Tchuem Tchuenté et al., (2012) reported a prevalence of S. haematobium of only 1.72%. Since a large sample size would be required to assess the impact of this pathogen on the Ab response to malaria, S. haematobium was not included in the study.*

• Were the individuals asymptomatic to intestinal parasites infection too? No diarrhea, abdominal pain, etc.? Please clarify.

*Reply: Yes. To make the point clear, the Methods section has been revised and states that all children with clinical cases of malaria or intestinal parasites were not included in the study and referred to the local clinic/hospital by the attending physician for treatment. Thank you for the comment.*

• (Page 6) It was mentioned that Plasmodium parasitemia was quantified. Did the authors observe any correlation between the Plasmodium parasite burden and the levels of IgG responses to the antigens?

*Reply: As expected, there was no correlation between parasitemia and malaria antibody levels*.

• (End of Page 7) Please specify: If the cut-off is MFI+3*SD, how the standard deviation was calculated if the negative controls were pooled? Was this experiment repeated or used replicates? Traditionally, the negative controls are tested simultaneously in different wells of the plate, and the cut-off is calculated from those values.

*Reply: Pooled negative control plasma sample were run in triplicates on the same plates as the test samples in all experiments, as well as the positive controls. The cut-off was obtained by calculating MFI+3 SD of the triplicates on all plates in the experiment.*

• Did the authors analyze the effect of helminth parasite burden (number of eggs/gram of stool) in those individuals with helminths? This valuable information was commented on but never included in the analysis. If not used,e I do not see the necessity of describing in the methods section

*Reply: The information has been deleted from the Methods section.*

• For data analysis:
• Before using ANOVA, did the authors checked for the normality of the variables? If yes, please specify, if not, calculate the normality of the variables and the other ANOVA assumptions.

*Reply: Yes, ANOVA was used to compare difference in age across the 4 groups (Table 2). However, comparisons of Ab MFI, which are not normally distributed, with age (Fig. 1) were performed using the Kruskal-Wallis test. The Methods section (Data analysis) has been revised. Information in Fig. 1 legend was correct.*

• If the authors have not-normal variables, they should use the Kruskal-Wallis non-parametric, and Dunn posthoc tests to verify differences between groups.

*Reply: Sorry for the mistake in the Methods section. The Kruskal-Wallis nonparametric test was performed in Fig 1 and 2. A posthoc test was not performed, as the goal was not to determine when peak Ab levels were obtained, but to determine if age had an influence on Ab levels. Since age was a variable, data for all age groups could not be combined, but rather age was taken into consideration during data analysis.*

• Please check frequencies described in table 1 (MAL+IP- 58.8%) vs. the values reported in the second line page 9. (59.4%).

*Reply: 59.4% is the correct value. The text has been revised.*

• Sum of 58.8%+16.9% = 75.7% not 75.6%.

*Reply: Thank you for catching the error. The values in Table 1 and text have been revised and are now consistent.*

• In table 1, please add a column with P-values to facilitate the interpretation of the differences between groups. Please report statistics of multiple comparisons between groups too.

*Reply: The comparisons requested by the reviewer were originally provided in the Table legend. To comply with the request, the p values have been moved to a column labeled “p values” and the method of analysis was retained in the Table legend.*

• What is the potential hypothesis to explain the increased values of parasitemia in the coinfected group?

*Reply: There is no significant difference in parasitemia between the two groups (p=0.1599). In fact, the higher parasitemia was found in young children who were intestinal parasite-negative (probably because very young children were in this group).*

• Please comment in the text the presence of multi-parasitism in the studied individuals.

*Reply: We thank the reviewer for the comment. The following sentence has been added to the Results section. “Interestingly, all of the children had single parasite infections, and polyparasitism was not found.”*

• (Page 11 table 3). Please include values of anemia and eosinophilia in individuals coinfected. In the current configuration is constructed is hard to determine the coinfection impact in anemia and eosinophilia values.

*Reply: Table 3 was designed to evaluate the influence of* ***age*** *on malaria, IP, anemia and eosinophilia. The number of co-infections are too small to be divided by age. In an attempt to* *address the Reviewer’s comment, a separate Table was designed that compares the influence of no infections, malaria-positive only, and co-infections on percent with anemia and eosinophilia*. *The Table will be up-loaded as supplemental Table 1. It essentially showed that same results as expected, anemia was associated with malaria and eosinophils were associated with co-infections*.

• (Page 11). In the sentence, "Thus, as children living in these villages increased with age, they developed partial immunity to malaria and anemia declined; whereas, the prevalence of IP and eosinophilia increased." In this sentence, it is necessary to specify that "protection" is protection against malaria symptoms. The table clearly shows that the frequency of malaria does not decrease with age, only the anemia.

*Reply: The sentence has been revised to read:* *“Thus, as children living in these villages increased with age, they began developing partial immunity to malaria symptoms and anemia declined; whereas, the prevalence of IP and eosinophilia increased.*

• Please plot Age vs. Antibody levels for each protein to verify the correlation for each protein studied.

*Reply: The figure on the right confirms that Ab levels increase with age. The figure shows a linear regression analysis of Ab levels for MSP1, MSP2, MSP3 and EBA-175 using data from all 320 children, and includes the equation for the regression line, the R^2^ value (all positive), and p value (all significant). Thus, the figure confirms that Ab levels increase with age. We do NOT wish to include this figure in the MS since it is essentially identical to the one shown in Fig 1 B, C, D and E. In fact, we feel that the information in Fig 1B-E is easier for the reader to understand.*

*Note: If the figure is not shown, it is provided in a separate document.*

• As an exploratory analysis, I suggest joining all data and make a boxplot comparing MFI between MAl-PI-, MAL-PI+, MAL+PI-, and MAL+PI+. Mainly for MSP1, MPS2, and MSP3 group age 3-10 and 11-15 to check.

*Reply: We thank the Reviewer Thanks for the suggestion concerning exploratory analysis. A comparison of Ab levels in two of the above groups (MAL-,IP-, and MAL+,IP-) is shown in Fig 2. Unfortunately, the number of children in the MAL-,PI+ group is too small to provide valuable information. As stated above, children in the MAL-,PI+ group (n=54) are infected with a variety of intestinal helminths, cestodes and protozoa (see Table 2). With such a diverse range of pathogens, plotting the data as a boxplot will not provide useful information. In Fig. 2, the distribution of Ab levels in children co-infected with malaria and single intestinal pathogens is provided. We feel this approach is more informative than “dumping all pathogens together.”*

• The sentence "E. histolytica is a gut amoeba that causes both intestinal and extraintestinal infections such as amebic colitis (dysentery) and liver or brain abscess. The protozoa cause a marked down-regulation of macrophage functions rendering the cells incapable of antigen presentation and unresponsive to cytokine stimulation (57)" does not explain the increase of antibody production in E. histolytica infected group. Why could a diminishing antigen presentation generate higher levels of anti-Plasmodium antigens?

*Reply: Very true! Not sure why that statement wasn’t caught. The Discussion has been changed significantly. It now reads, “The decrease in macrophage function does not explain the increase in Ab to EBA-175. One possible explanation is that since malaria and E. histolytica…”*

Other observations/questions:
• In the title, add "IgG" to Antibody response. *Reply: IgG has been added to title* *(although not all of the co-authors agree this is necessary).*

• Check all scientific names of parasite species for correct formatting in italics. (Example Entamoeba histolytica in the Results section in the abstract)

*Reply: The scientific name has been checked and are now in italics.*

• Please, mention in the background the region where the study was performed.

*Reply: This information was included in the background section of the Abstract. It is also included in the Materials section.*

• It is necessary to describe and discuss the role of MSP1, MPS2, MSP3, and EBA-175 as markers in serological studies.

*Reply: This information has been added to the Discussion*.

• Considering that coinfection prevalence is relatively low, I consider that it is important to discriminate with colors or point shapes the individuals MAL-IP-, MAL+IP-, MAL-IP+, MAL+IP+ in Figure 1 B-C-D-E

*Reply: We thank the Reviewer for the suggestion. However, information in Fig 1B-E is designed to address the question, are Ab prevalence and levels influence by age? Whereas, Fig 2 provides comparisons between individuals infected with malaria alone or co-infected with specific intestinal parasites. Thus, colored dots or symbols are not needed in Fig 1 (and could be confusing to the reader).*

• In page 6 subtitle "Laboratory detection, quantification and speciation of malaria parasites.", I will not use speciation here. I suggest "Diagnosis and quantification of Plasmodium sp. parasites.

*Reply: The header has been changed to read: “Laboratory detection of malaria parasites.”*

• (Page 14-15) What type of parasite is "Amoeba"? What is the difference between "Amoeba" and E. histolytica? Traditionally, E. histolytica is considered an amoeba too.

*Reply: The figure has been revised to read Intestinal Protozoa. Thanks for pointing out the mis-classification.*

• In table 1, to facilitate reading, please remove symbols % and /ul located in cells with data and add to the columns describing the variables.

*Reply: The symbols in the data cells have been removed.*

• For consistency, unify parasitemia vs. parasitemia, anemia vs. anemia in the text and plots.

*Reply: The British spelling of parasitaemia, anaemia, and haemoglobin have been used through out the MS.*

• (Page 10) change "The major of helminth parasites" to "The most frequent helminth species detected."

*Reply: The change was made as suggested.*

• (Table 2) Check all the total numbers for the "Total IP+" column. For example, for protozoans, the sum is 29+19+4 = 48, and it was reported 47

*Reply: This has been verified and corrected to 48 in Table 2*

• (Page 13) In plot titles Change Ab (Antibody) to IgG

*Reply: We thank the Reviewer for the comment, but decide not to make the change. Our rationale is that by definition, IgG is a class of immunoglobulin found in the blood; whereas, Ab are plasma proteins that bind specifically with an antigen. What was measured was IgG Ab. Since the serological assay measured IgG Ab that were recorded as MFI (median fluorescence intensity), we think the labels on the Y-Axis (Ab levels -MFI) reflect what was done. The Methods section makes it clear that the Ab were of the IgG class. [Note: Serum IgG levels (which implies mg/ml) were not measured.]*

• (Figure 1E) Add, Change from EBA to EBA-175.

*Reply: Change has been made.*

• Please verify all references formatting (For example, reference 42 is all in capital letters)

*Reply: References have been edited as requested by the reviewer.*

**Review #3**: Comments were in the attachment.

*Reply: In revising the MS, all requested changes were made and additional information provided in the text, including information on the BLAST search. The only request we would not fully address is the prevalence of bednet use in the villages. The only information available is that very few children use bednets. Since the slide-positivity rate of 75.6% for P. falciparum, it is unlikely the bednets are having a major influence on the current study. The following information has been added to the MS in the Results section. “To determine if higher Ab levels in children co-infected with P. falciparum and E. histolytica might be due to cross-reactive epitopes, a BLAST search for sequence homology between EBA-175 and E. histolytica proteins was made. No similarities were found using Metablast, and only one hit was found using discontinuous metablast which had a span of only 38 nucleotides (~12 amino acids). Thus, there does not appear to be shared epitopes between these two pathogens that would explain the increase in Ab to EBA-175 in children with co-infections.”*

**Figure for Reviewer #2 confirming an increase in antibody levels with age.**
